# Stereoselective Synthesis of Benzoylated Gulmirecin A and Disciformycin B

**DOI:** 10.1021/acs.orglett.4c03727

**Published:** 2025-02-12

**Authors:** Klaus-Peter Rühmann, Kaijie Ji, Dirk Trauner

**Affiliations:** Department of Chemistry, New York University, New York, New York 10003, United States

## Abstract

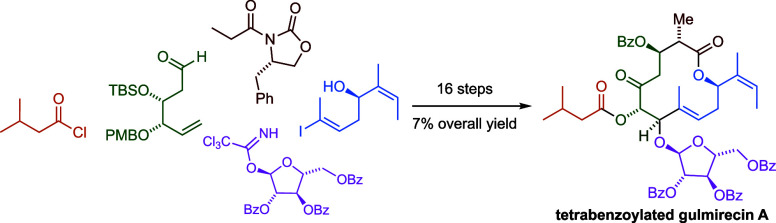

Disciformycins and gulmirecins are potent inhibitors of the bacterial RNA polymerase. They show promising activities against multidrug-resistant pathogens often found in clinical settings, such as methicillin- and vancomycin-resistant *Staphylococcus aureus* (MRSA and VRSA). Our modular, high-yielding, and scalable synthetic approach started with the desymmetrization of divinyl carbinol and provided benzoylated precursors for both natural product classes. The 12-membered macrocycle was assembled by a stereoselective Nozaki–Hiyama–Takai–Kishi macrocyclization.

The World Health Organization (WHO) describes antibiotic resistance as one of the greatest threats to human health, and without continued research, the consequences might be catastrophic.^1^ Despite the enormous economic challenges, the development of novel antibiotics remains an important goal in both academia and industry.^2^ Most clinically used antibiotics target either the ribosome, interfere with bacterial cell wall biosynthesis, or inhibit DNA topoisomerases and gyrases.^3−5^ Very few inhibitors of RNA polymerase (RNAP) are clinically used as antibiotics, although many natural products and synthetic compounds (**1**–**10**) have been identified that inhibit this essential enzyme (Figure 1A).^6^ While in eukaryotes different RNAP subtypes are responsible for the transcription of a distinct subset of RNA, prokaryotes and archaea only contain a single universal DNA-dependent RNA polymerase.^7^ The enormous success of rifamycin and its semisynthetic derivatives for the treatment of various bacterial infections demonstrates the value of bacterial RNAP as a molecular target for antibacterial therapies.^8^ Most inhibitory compounds bind either around the active catalytic center or the switch region, which mediates opening and closing of the multi-subunit complex.^8−13^ Many of these natural products have been synthesized previously; however, only rifamycin has undergone extensive structure–activity relationship (SAR) studies.^14−19^ An innovative, modular, and efficient access to other inhibitors of the bacterial RNAP can initiate further effective SAR studies that could lead to the development of novel clinically useful antibiotics. We decided to focus on the disciformycin and gulmirecin natural product family as our lead compounds, which have been isolated around the same time from two myxobacterial strains, *Pyxidicoccus fallax* AndGT8 and HKI 727, respectively (Figure 1B).^20,21^ They show potent activities against various Gram-positive bacteria, including methicillin- and vancomycin-resistant *Staphylococcus aureus* (MRSA and VRSA; Figure 1C). Mutation studies indicate that these natural products target RNA polymerase without exhibiting a cross-resistance with rifampicin.^22^ The exact binding site of these 12-membered macrolactones has not yet been disclosed.

**Figure 1:**
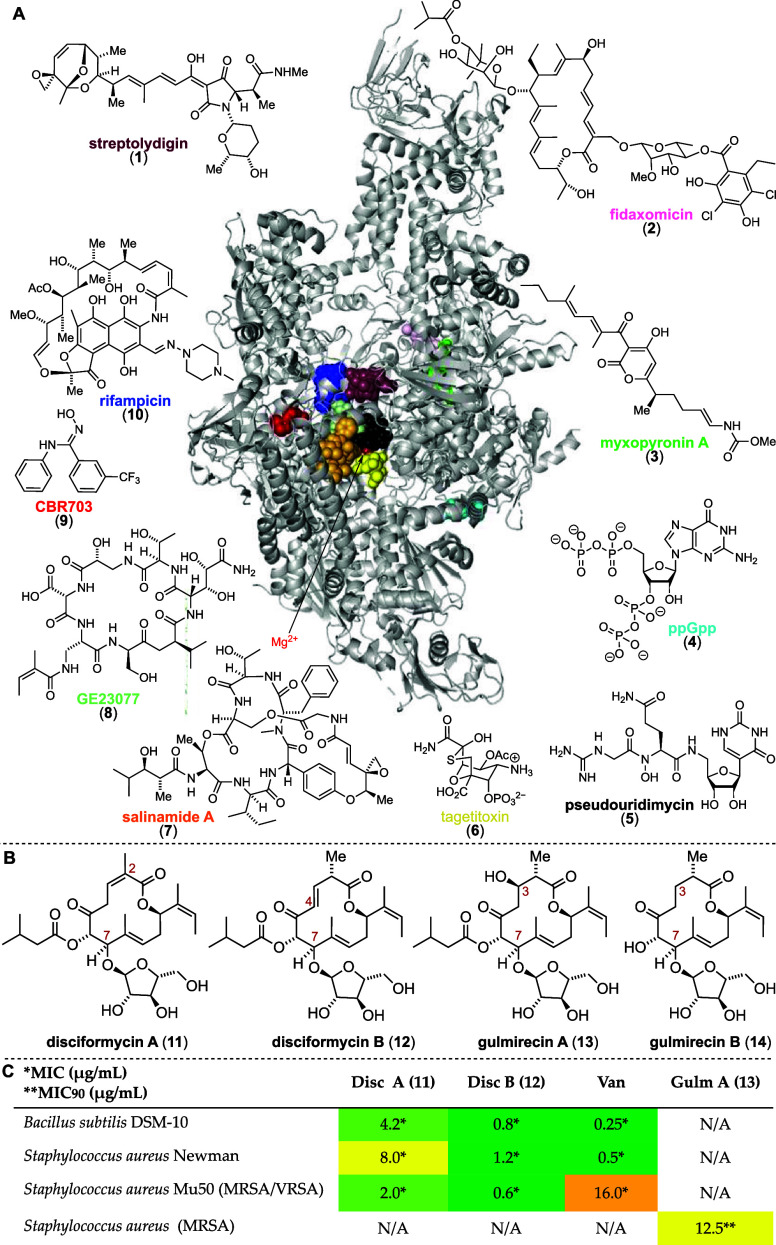
(A) Binding map of 10 bacterial RNA polymerase inhibitor classes. Only rifamycins and fidaxomicin are used in clinical settings. (B) Chemical structures of the discifomycins and gulmirecins. (C) Minimum inhibitory concentration (MIC) values for disciformycins (Disc A, **11**; Disc B, **12**),^21^ gulmirecin A (Gulm A, **13**),^20^ and vancomycin (Van)^21^ for comparison.

The four members of this natural product family strongly resemble each other. A 12-membered macrolactone, containing one or two endocyclic olefins and three to five stereogenic centers, is decorated with α-d-arabinofuranose and isovaleryl ester. The two disciformycins differ in the position of one of their endocyclic olefins being in conjugation with either of two carbonyl functionalities. Unsaturated ester in disciformycin A (**11**) and enone in disciformycin B (**12**) are in equilibrium and interconvertible.^23^ Gulmirecins lack this unsaturation and either possess an (*R*)-hydroxy group at C3 (gulmirecin A, **13**) or are fully reduced (gulmirecin B, **14**). Gulmirecin B further lacks isovaleryl ester, resulting in a significant loss of activity.^20^ To date, two total syntheses of disciformycin B have been completed by the groups of Fürstner and Altmann; three further synthetic studies toward this natural product class were conducted by the groups of Kirschning, Maier, and Ichikawa.^23−27^ An analysis of the previous approaches provided us with the following insights: (i) Many reaction conditions initiate isomerization of the C3–C4 endocyclic olefin when targeting disciformycins.^23^ (ii) The key to success will be a careful orchestration of the protecting groups that avoids acidic deprotection conditions of advanced compounds.^26^ (iii) Ring-closing metatheses using alkenes^27^ or alkynes^23^ and macrolactonization^24^ strategies successfully established the 12-membered core, while a reductive coupling between C7/C8 provided the macrolactone with the wrong stereochemistry of the C7 alcohol.^26^ A metalate fragment coupling failed to create the same C7/C8 bond.^25^

Our retrosynthetic analysis is outlined in Scheme 1. We reasoned that an approach toward gulmirecins would avoid the problems occurring from isomerization of the C3/C4 enone, while an elimination of the C3-hydroxy group would still allow for a rapid conversion toward disciformycins. We envisioned the assembly of the orthogonally protected macrolactone via a Nozaki–Hiyama–Takai–Kishi (NHTK) macrocyclization, an esterification, and an Evans *syn*-aldol reaction. We hoped that the proposed intramolecular NHTK reaction would be diastereoselective due to favorable pre-organization of the precursor but also considered the use of chiral ligands for the intermediary chromium complex.^28^ Our dissection strategy provided us with four key building blocks: aldehyde **21**, Evans oxazolidinone **16**, vinyl iodide **19**, and glycosyl donor **22**. Aldehyde **21** was traced back to divinyl carbinol **20**, and vinyl iodide **19** was derived from angelic methyl ester **17**.

**Scheme 1:**
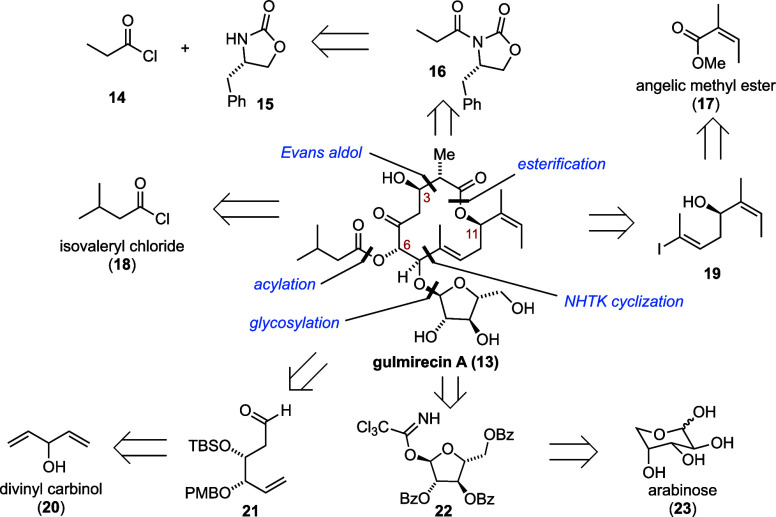
Retrosynthetic Dissection of Gulmirecin A

Our synthesis started with an optimized desymmetrization and benzylation protocol to prepare epoxide **24** from divinyl carbinol **20** (see the Supporting Information).^29^ The epoxide in compound **24** was opened by heating with potassium cyanide, affording cyanohydrin **25** (Scheme 2A). *tert*-Butyl(dimethyl)silyl (TBS) protection was followed by diisobutylaluminium hydride (DIBAL) reduction to obtain aldehyde **21**. This five-step reaction sequence proved to be robust, practical, and highly scalable (multigram scale).

**Scheme 2:**
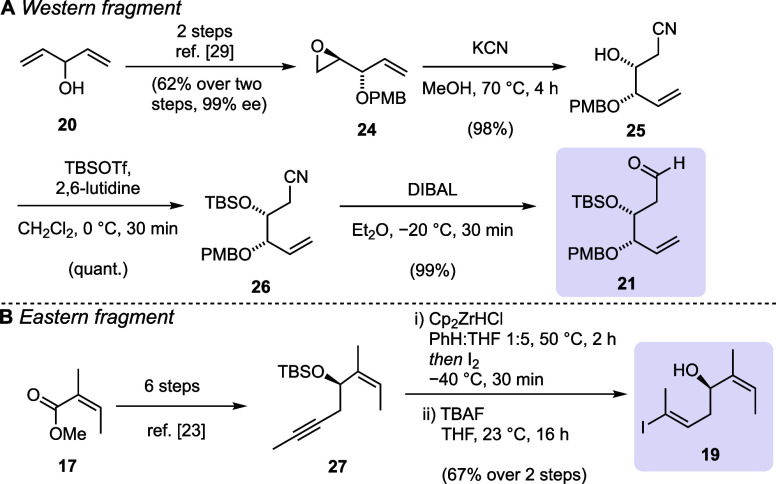
Synthesis of Building Blocks **19** and **21**

Vinyl iodide building block **19** was prepared in eight steps from angelic methyl ester **17** (Scheme 2B). Following a procedure by Fürstner and co-workers, alkyne **27** was prepared in six steps.^23^ A hydrozirconation/iodination sequence then afforded two vinyl iodides with a 9:1 regioselectivity, favoring the desired vinyl iodide (not shown; see the Supporting Information). Both regioisomers were inseparable at this stage, but they could be isolated in pure form after desilylation using tetrabutylammonium fluoride (TBAF) solution. The overall eight-step sequence was scalable, although special care had to be taken concerning the volatility of all intermediates. The synthesis of the remaining two building blocks, oxazolidinone **16** and arabinose donor **22**, was accomplished following modified literature procedures.^30−33^

Evans aldol reaction of oxazolidinone **16** and aldehyde **21** afforded *syn*-aldol product **28** with excellent diastereoselectivity and in almost quantitative yield (Scheme 3). The addition of stoichiometric amounts of *N*-methylpyrrolidinone (NMP) was crucial for the success of this reaction, as it improved the yield of this transformation from moderate (48–62%) to excellent (96–99%).^34^ Benzoylation of aldol product **28** required high temperatures and excess benzoyl chloride. Steric hindrance likely prevented silyl migration to occur under these conditions. Benzoate **29** was subjected to lithium-hydroperoxide-mediated cleavage of the oxazolidinone auxiliary, which afforded carboxylic acid **30**. The timing for quenching this reaction was stringent, because prolonged exposure to the reaction conditions led to debenzoylation and other side products. Ozonolysis smoothly converted terminal alkene **30** to aldehyde **31**.

**Scheme 3:**
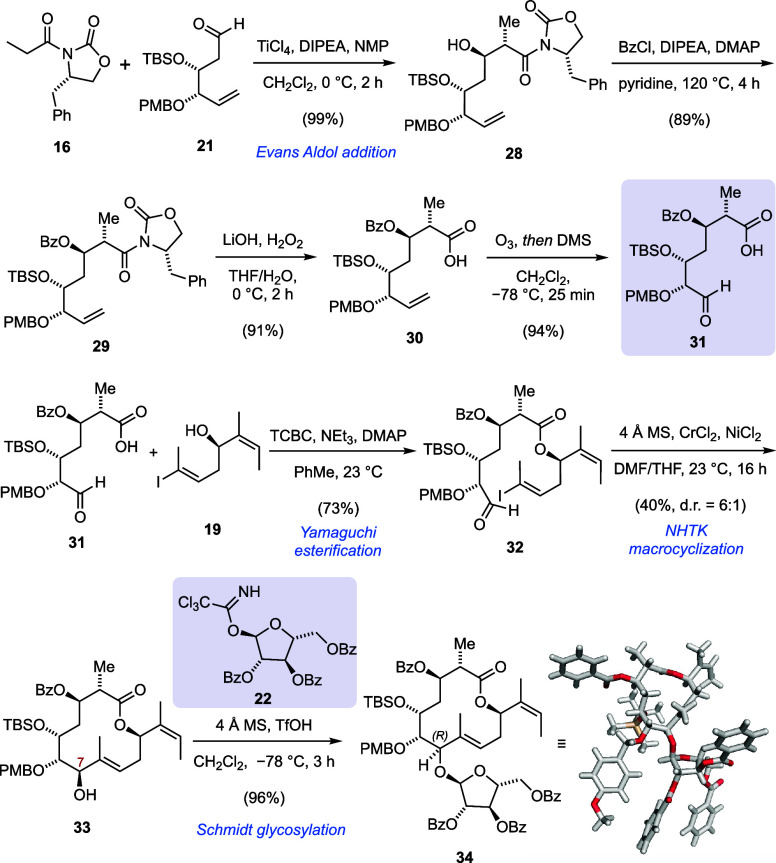
Merging the Building Blocks toward Orthogonally Protected Macrolactone **34**

Our next task was the formation of the macrocycle. Initially, we considered Ni(0)-mediated reductive coupling conditions to assemble the macrolactone, similar to the approach by Ichikawa and co-workers.^26,35^ As an alternative strategy, we envisioned the macrocyclization to occur via the use of a Nozaki–Hiyama–Takai–Kishi (NHTK) reaction. To this end, we coupled carboxylic acid **31** with alcohol **19** using Yamaguchi’s conditions.^36^ Treatment of the obtained macrocyclization precursor **32** with excess chromium(II) chloride (20 equiv) alongside catalytic amounts of nickel(II) chloride afforded a separable mixture of C7 diastereomers in a 6:1 ratio.^37^ Absolute identification of the established C7 stereocenter proved challenging at this stage, especially because both isomers were isolated as mixtures of macrocyclic conformers, and high-temperature nuclear magnetic resonance (NMR) experiments were necessary to converge the ^1^H signals. Instead, we continued our synthesis and installed the arabinose glycoside for our major NHTK product **33**. Standard Schmidt glycosylation conditions using trichloroacetimidate **22** afforded macrocycle **34** as a single diastereomer and in excellent yield due to anchimeric assistance. X-ray analysis of the obtained crystalline intermediate confirmed the desired C7 (*R*) configuration, which was set by NHTK macrocyclization. The observed product corresponds to an anti-Felkin–Ahn addition and stands in contrast to the typically observed selectivity of organochromium(III) additions to α-alkoxy aldehydes.^38,39^ Instead, we believe that the conformational bias of our substrate dictates its pre-organization and the resulting selectivity of the addition.^40^

With macrolactone **34** in hand, we explored conditions that would lead to the gulmirecin and disciformycin natural products (Scheme 4). A 2,3-dichloro-5,6-dicyano-1,4-benzoquinone (DDQ)-mediated *p*-methoxybenzyl (PMB) deprotection was successful when a neutral phosphate buffer was added as a co-solvent, yielding secondary alcohol **35**. The subsequent acylation with isovaleryl chloride required a large excess (50 equiv) of the acid chloride and prolonged heating conditions but ultimately afforded isovaleryl ester **36** in excellent yield. For the deprotection of silyl ether in compound **36**, extensive optimizations were necessary because both basic (TBAF) and acidic (HCl, HF, and TASF) conditions resulted in an acyl migration of the isovaleryl ester and formation of other unidentified side products. The solution to this problem was an *in situ* preparation of pyridine hydrofluoride by equimolar addition of the base and an aqueous solution of hydrofluoric acid to a solution of silyl ether **36** in acetonitrile. These conditions afforded 75% of the desired deprotected alcohol **37a** alongside small amounts of an acyl migration product (12%, **37b**, shown in the Supporting Information). It is worth noting that the commercially available reagent led to decomposition of the substrate. Dess–Martin periodinane (DMP) oxidation afforded the 4-fold benzoylated natural product **38**. The β-keto benzoate in product **38** was unstable under silica or high-performance liquid chromatography (HPLC) purification conditions. A short plug of silica was sufficient in removing residual iodinane. The presence of acidic buffer during HPLC purification led to a selective elimination of C3 benzoate, which, in fact, would enable synthetic access toward disciformycin B (**12**), if complete debenzoylation of precursor **39** was successful.

**Scheme 4:**
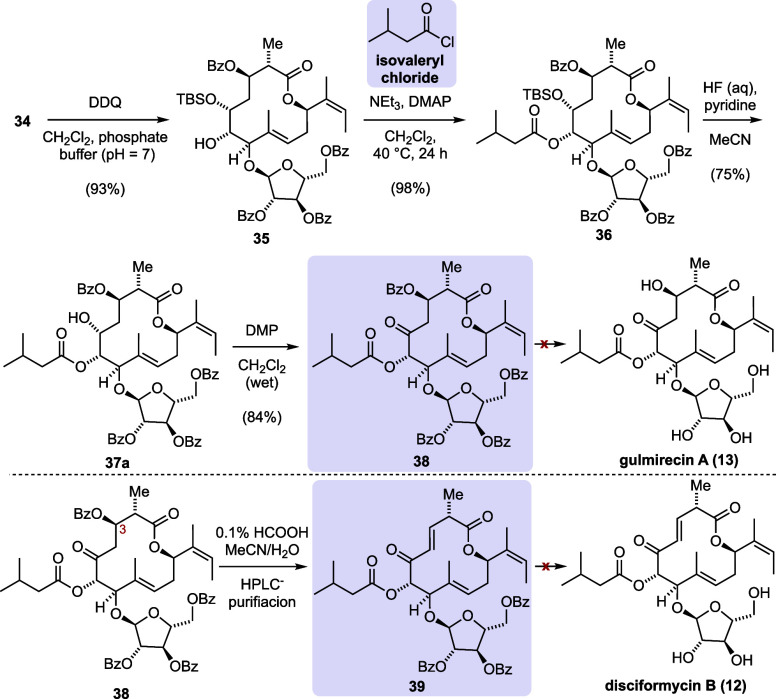
Synthesis of Benzoylated Gulmirecin A and Disciformycin B Precursors **38** and **39**

The final global debenzoylation step of precursor **38**, however, evolved into a major obstacle in our synthesis. Although the literature reported many mild debenzoylation methods compatible with macrolactones, none of our attempts achieved complete deprotection (Table 1).^41−47^ Our conditions were either too mild and did not lead to any reaction (methanolysis, entry 1) or only initiated a slow and partial hydrolysis (mildly basic conditions, entries 3 and 4; Me_3_SnOH in methanol, entry 5; mild Lewis acids, entries 9–11; mild nucleophiles, entries 13 and 14; and transesterification using Otera’s catalyst, entry 18). Harsher reaction conditions resulted in decomposition and the formation of complex unidentifiable mixtures (strongly basic, entries 2 and 3; strongly acidic, entries 7 and 8; strong nucleophiles, entry 12; and electrochemical conditions, entry 15). Enzymatic attempts were equally unsuccessful (entries 16 and 17).

**Table 1:** Selection of Global Debenzoylation Attempts

entry	conditions	outcome
1	MeOH, 60 °C, 60 days	no reaction observed
2	K_2_CO_3_, MeOH, 60 °C	macrolactone cleaved, many side products
3	NaOH, MeOH	partial debenzoylation and elimination of C3 OBz
4	DBU, MeOH	partial deprotection, mixture of products
5	Me_3_SnOH, MeOH	partial deprotection of primary benzoate
6	Mg, MeOH	partial transesterification
7	HCl, MeOH	decomposition
8	Sc(OTf)_3_, MeOH, H_2_O	decomposition
9	Yb(OTf)_3_, MeOH, H_2_O	partial deprotection, mixture of products
10	Ho(OTf)_3_, MeOH, H_2_O	partial deprotection, mixture of products
11	Dy(OTf)_3_, MeOH, H_2_O	partial deprotection, mixture of products
12	EtSH, K_2_CO_3_, 60 °C, 2 days	decomposition
13	PhSH, K_2_CO_3_, MeOH	slow conversion, deprotection of primary benzoate
14	PhNH_2_, MeOH	slow conversion, deprotection of primary benzoate
15	electrolysis: 2.05 V	decomposition
16	lipase from *Mucor miehei*	partial deprotection, mixture of products
17	lipase B *Candida antarctica*	partial deprotection, mixture of products
18	Oteras’ catalyst, BnOH	partial deprotection, mixture of products

In summary, this study outlines a synthetic approach toward the antibacterial natural product family of disciformycins and gulmirecins. We have established and optimized a versatile synthetic route that afforded a 4-fold benzoylated precursor to gulmirecin A over 16 steps in the longest linear sequence and an overall yield of 7%. The 3-fold benzoylated precursor to disciformycin B was prepared in one additional step. Key steps of our approach are the enantiotopos- and diastereoface-selective Sharpless epoxidation of divinyl carbinol,^29^ a *syn*-Evans aldol reaction, and an unusual, selective Nozaki–Hiyama–Takai–Kishi macrocyclization. Unfortunately, even after extensive experimentation, none of our global debenzoylation attempts afforded either of the natural products. We are currently investigating an alternative protecting group scheme that utilizes our synthetic insights and will allow for completion of the synthesis. Many synthetic steps have already been optimized on large scales, which will benefit our planned late-stage derivatization program to systematically investigate structure–activity relationships of disciformycins and gulmirecins.

## Data Availability

The data underlying this study are available in the published article and its Supporting Information.

## Supporting Information Available

Detailed experimental procedures with characterization data for new compounds and crystallographic data for compound **34** (PDF)
