# Supplementary material for: Stereoselective Synthesis of Benzoylated Gulmirecin A and Disciformycin B
Source: Org Lett. 2025 Feb 12;27(7):1584–9. doi: 10.1021/acs.orglett.4c03727 (PMC11852209; doi:10.1021/acs.orglett.4c03727)
Supplement: Supplementary file 1 — ol4c03727_si_001.pdf [file ol4c03727_si_001.pdf]

Supporting Information for

Stereoselective Synthesis of Benzoylated Gulmirecin A and Disciformycin B

Klaus-Peter Rühmann[1,2], Kaijie Ji[1,3], Dirk Trauner[1,4,*]

[1] Department of Chemistry, New York University, New York, New York 10003, United States

[2] Present address: Institute for Systems Genetics at Innolabs, NYU Langone Health, 45-18 Ct Square W, Long Island City, NY 11101.

[3] Present address: Departments of Chemical Development and Material and Analytical Sciences, Boehringer Ingelheim Pharmaceuticals, Inc., Ridgefield, Connecticut 06877, United States

[4] Present address: Department of Chemistry and Department of Systems Pharmacology and Translational Therapeutics, University of Pennsylvania, Philadelphia, Pennsylvania 19104, United States

*E-mail: dtrauner@upenn.com

TABLE OF CONTENTS

1 EXPERIMENTAL SECTION **3**

1.1 MATERIAL AND METHODS 3

1.1.1 Equipment and Instruments *3*

1.1.2 Methods *5*

1.1.3 Chemicals *6*

1.2 EXPERIMENTAL PROCEDURES 8

1.3 CRYSTALLOGRAPHIC DATA 125

1.4 REFERENCES 127

1 Experimental section

1.1 Material and Methods

1.1.1 Equipment and Instruments

Nuclear magnetic resonance (NMR) spectroscopy:

NMR-spectra were acquired with the following spectrometers: Bruker Avance III HD 400 equipped with a CryoProbe™ (400 MHz for ^{1}H and 101 MHz for ^{13}C spectroscopy), Bruker Avance 400 equipped with a 5 mm multinuclear broadband observe (BBO) probe for Variable Temperature (VT) NMR experiments (400 MHz for ^{1}H and 101 MHz for ^{13}C spectroscopy), Bruker Avance NEO 500 equipped with a CryoProbe™ (500 MHz for ^{1}H and 126 MHz for ^{13}C spectroscopy), Bruker Avance III 600 equipped with a CryoProbe™ (600 MHz for ^{1}H and 151 MHz for ^{13}C spectroscopy), or Bruker Avance NEO 800 equipped with a Cryoprobe™ (800 MHz for ^{1}H, 201 MHz for ^{13}C spectroscopy). Chemical shifts (δ) are reported in parts per million (ppm) relative to tetramethylsilane (TMS). The residual solvent signals of deuterated solvents $CDCl_3$, C_6D_6, toluene -d_8, were used as internal references. Spin multiplicities are described as follows: s (singlet), d (doublet), t (triplet), q (quartet), m (multiplet), br (broad) or a combination thereof. Structural analysis was conducted by interpretation of ^{1}H- and ^{13}C-NMR spectra with the aid of additional 2D spectra (COSY, HMBC, HSQC, NOESY) using the software MestReNova v.10.0.1-14719.

Mass spectrometry (MS):

Liquid chromatography mass spectrometry (LC-MS) analysis was performed on an *LCMS 1260 Infinity II Agilent Technologies* system (Windows 10, OpenLabs CDS Chemstation Software, *6120 Quadrupole LC/MS G7111B* quaternary pump, *G7129A Infinity II* vial sampler, *G7117C 1260 diode array detector*) equipped with an *LC Kinetex column* 2.6 µm C18 (50 x 3 mm) at room temperature. Runs were performed at a flowrate of

1 mL/min with a gradient of 5 to 100% acetonitrile in water over 5 min. Mobile phase gradients of acetonitrile and water contained each 0.1% formic acid. High-resolution MS (HRMS) spectra were recorded on Agilent 6224 Accurate-Mass LC/TOF spectrometer using APCI (atmospheric pressure chemical ionization) or ESI (electrospray ionization). Prior to injection, samples were filtered through a syringe filter (VWR® PTFE syringe filters, 13 mm diameter, pore size 0.2 μm).

Infrared spectroscopy (IR):

IR spectra were recorded on a Thermo Nicolet AVATAR Fourier Transform Infrared Spectrometer (FTIR) using an attenuated total reflection (ATR) measuring unit. For measurements, the neat substances were directly applied as a thin film on the ATR unit. The measured wavenumbers are reported with their relative intensities which were classified as: s (strong), m (medium), w (weak), br (broad) or combinations thereof.

Optical rotation ($[\propto]_D^T$):

Optical rotation values were measured on a Jasco P-2000 polarimeter. The specific rotation ($[\propto]_D^T$) values are reported in deg·dm^{-1}·mL·g^{-1} and calculated by the formula:

$$[\propto]_D^T = \frac{\alpha \cdot 100}{l \cdot c}$$

T represents the ambient temperature (°C), *D* represents the wavelength (in all cases, D is the sodium line: 589 nm), l represents the length of the cuvette (dm), c represents the concentration of the solution (g/100 mL), α represents the measured rotation in degrees. The appropriate solvent and concentrations are reported in brackets.

High Performance Liquid Chromatography (HPLC):

A 1260 Infinity Agilent Technologies system (Windows 10, OpenLabs CDS Chemstation Software, two G1361A preparative pumps, G2260A autosampler with 2400 μL maximum injection volume, G1170A

column switching valve, G7115A diode array detector equipped with a 0.3 mm preparative flow cell, G1364B fraction collector) was used for preparative high performance liquid chromatography using a semipreparative column (Phenomenex, Gemini 5 µm C18 110 Å, 150 x 10 mm, product #OOF-4435-N0). Mobile phase gradients of acetonitrile and water contained 0.1% formic acid each. Samples were filtered through a syringe filter (VWR® PTFE syringe filters, 13 mm diameter, pore size 0.2 µm).

X-Ray Diffractometer

A Bruker AXS SMART APEXII Single Crystal Diffractometer was used to obtain the single crystal X-ray data for compound **34**. The measurement and analysis was conducted by Dr. Tony Hu.

1.1.2 Methods

Unless otherwise noted, all reactions were magnetically stirred under inert gas (N_2) atmosphere using standard Schlenk techniques. Glassware was evacuated and either flame dried with a propane torch. Drying over $MgSO_4$ or Na_2SO_4 implies stirring with an appropriate amount of anhydrous salt for several minutes followed by filtration through a glass frit and rinsing of the filter cake with additional solvent. Electric heating plates with either heating mantles or oil baths were used for reactions at elevated temperatures. For reactions below room temperature, the reaction vessel was cooled using a mixture of ice and water (0 °C), a slurry of sodium chloride and ice (−20 °C), acetonitrile and dry ice (−40 °C), acetone and dry ice (−78 °C), acetone and liquid N_2 (−94 °C) or using a JULABO FT902-Cryostat. Stated reaction temperatures refer to the external bath temperature. Cannulas and syringes were used for the transfer of reagents and solvents, which were flooded with inert gas (3×) before use. Purification by column chromatography was performed using either manual air pressure or a *Teledyne Isco Combiflash R_f+ UV* purification system on Geduran© Si60 silica gel (40–63 µm) from Merck KGaA. Silica gel F_{254} TLC plates from Merck KGaA were used for monitoring reactions, analyzing fractions of column chromatography,

and measuring R_f values. To visualize the analytes, TLC plates were irradiated with UV light and/or treated with appropriate staining solutions followed by subsequent heating. Freeze-drying refers to freezing of the respective sample in liquid nitrogen followed by evacuating the containing flask with high vacuum (< 1 mbar) and slow thawing to rt. Reaction yields refer to spectroscopically pure isolated amounts of compounds.

1.1.3 Chemicals

All chemicals were purchased from *Acros Organics, Alfa Aesar, Cayman Chemical, Combi-Blocks, Fisher Scientific*, *Oakwood Chemical, Santa Cruz Biotechnology (SCBT), Sigma Aldrich*, *Strem Chemicals, or Tokyo Chemical Industry (TCI).* HPLC- and ACS grade solvents were purchased from *Fisher Scientific* and used as received without further purification. The expression "hexanes" refers to a mixture of hexane isomers with a boiling point between 40-80 °C. Unless otherwise noted, reactions were performed using dry solvents that were dried by passage through the Pure Solv-MD Standard Design Solvent Purification System (acetonitrile, dichloromethane, diethyl ether, methanol, tetrahydrofuran, triethylamine, toluene) before use. Other dry solvents were purchased from commercial sources (*Acros Organics*, *Fisher Scientific*) and were stored under an inert gas atmosphere and over molecular sieves. All other reagents with a purity of >95% were purchased from commercial sources and used without further purification, unless otherwise noted.

Staining solutions were prepared as follows:

$KMnO_4$ stain: Dissolve 7.5 g of KMnO4, 50 g K_2CO_3, and 6.25 mL 10% NaOH (or 8 mL 1M) in 1000 mL distilled water. A typical lifetime for this stain is approximately 3 months.

CAM stain: To 940 mL of distilled water was added 48 g of ammonium molybdate ($(NH_4)_6Mo_7O_{24}$ • 4 H_2O), 2 g of ceric ammonium sulfate ($Ce(NH_4)_4(SO_4)_4$), and 60 mL of concentrated sulfuric acid. This stain has a long shelf-life as long as solvent evaporation is limited.

Vanillin stain: Prepare a solution of 60 g vanillin in 990 mL ethanol and 10 mL concentrated sulfuric acid.

Ninhydrin stain: Dissolve 13.5 g of ninhydrin in 900 mL of *n*-butanol, then add 27 mL acetic acid.

Dinitrophenylhydrazine (DNP): Dissolve 36g of 2,4-dinitrophenylhydrazine, 180mL of conc. sulfuric acid, and 240mL of water in 600mL of 95% ethanol.

***p*-Anisaldehyde (PAA):** To 750 mL of absolute ethanol was added 35 mL of concentrated sulfuric acid, 10.5 mL of glacial acetic acid and 26 mL of p-anisaldehyde. The solution is then stirred vigorously to ensure homogeneity.

1.2 Experimental Procedures

Preparation of S01: Benzoylation of D-Arabinose[48]

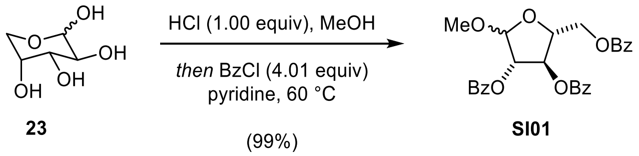

23 **SI01**

To D-arabinose (**23**) (10.0 g, 66.6 mmol, 1.00 equiv) in anhydrous methanol (200 ml) was added a solution of hydrogen chloride (3 M in methanol, 22.2 mL, 66.6 mmol, 1.00 equiv) and the mixture was stirred at room temperature until solution was complete. After 3.5 h dry pyridine (60 mL) was added and methanol was removed under reduced pressure. The residual syrup was dissolved in pyridine (75 mL, 932 mmol, 14.0 equiv), benzoyl chloride (32.0 mL, 275 mmol, 4.01 eq) was added and the reaction mixture was heated to 60 °C for 30 min using a heating mantle. Water (200 mL) and ethyl acetate (200 mL) were added and the phases were separated. The organic solution was washed successively with water (200 mL), 1.5 M sulfuric acid (150 mL) and aqueous saturated sodium bicarbonate solution (200 mL), was further dried over magnesium sulfate, filtered, and concentrated under reduced pressure affording a 4:6 mixture of α- and β-anomers of the desired tribenzoate **SI01** (31.6 g, 66.3 mmol, 99%) as a colorless viscous oil which was used without any further purification.

<u>**Characterization Data for SI01:**</u>

The recorded spectroscopic data matched the reported ones.[48]

R*f* (33% EtOAc in hexanes) = 0.71 (UV, $KMnO_4$).

^{1}H-NMR (400 MHz, $CDCl_3$): δ = 8.11 – 7.97 (m, 6H), 7.61 – 7.28 (m, 9H), 6.01 – 5.17 (m, 3H), 4.88 – 4.42 (m, 3H), 3.44 (2 x s, 3H) ppm.

Preparation of 22: Formation of Arabinose Donor 22[33]

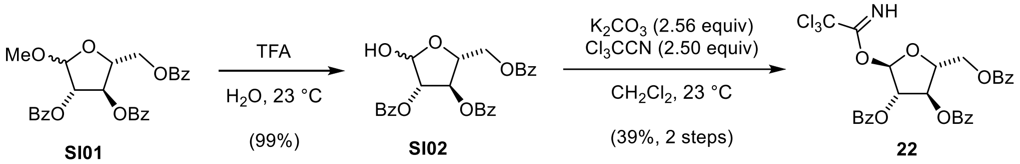

The 4:6 mixture of α- and β-methylarabinose-anomers **SI01** (1.00 g, 2.10 mmol, 1.00 equiv) was dissolved in a mixture of acetone (0.5 mL) and 90% aqueous trifluoracetic acid (3 mL, 35.3 mmol, 16.8 equiv). The resulting white heterogeneous slurry was stirred at room temperature for 6 h at the end of which TLC analysis (33% EtOAc in hexanes) indicated that the reaction was complete. Ethyl acetate (20 mL) and water (10 mL) were added and the aqueous phase was extracted three times. The combined organic phases were dried over anhydrous sodium sulfate, filtered, and concentrated under reduced pressure. The obtained crude product (959 mg, 2.07 mmol, 99%) was used for the subsequent transformation into glycosyl donor **22** without any further purification.

To a solution of the crude product **SI02** (951 mg, 2.06 mmol, 1.00 equiv) in anhydrous dichloromethane (20 mL) were added trichloroacetonitrile (600 μL, 5.39 mmol, 2.62 equiv) followed by potassium carbonate (711 mg, 5.14 mmol, 2.50 equiv). The reaction mixture was stirred at ambient temperature for 18 h upon which TLC analysis indicated complete consumption of starting the arabinose hemiacetals. The reaction mixture was filtered using a fritted Büchner funnel, and the filtrate was concentrated. The obtained viscous oil was purified by column chromatography over SiO_2 using a 10 to 25% EtOAc in hexanes gradient to afford trichloroacetamide **22** (489 mg, 0.806 mmol, 39%) as a viscous colorless liquid which solidified to a colorless amorphous solid upon storage. NMR analysis revealed residual impurities of ethyl acetate which were present even after extensive exposure to high vacuum.

Characterization Data for SI02:

R$_f$ (33% EtOAc in hexanes) = 0.54 (UV, $KMnO_4$).

Characterization Data for 22:

The recorded spectroscopic data matched the reported ones.[33]

R$_f$ (33% EtOAc in hexanes) = 0.35 (UV, $KMnO_4$).

^{1}H-NMR (400 MHz, $CDCl_3$): δ = 8.73 (s, 1H), 8.12 (dt, J = 8.4, 1.5 Hz, 2H), 8.03 (dq, J = 8.4, 1.6 Hz, 4H), 7.61 (tdt, J = 6.8, 3.0, 1.5 Hz, 2H), 7.52 – 7.39 (m, 5H), 7.30 – 7.25 (m, 6H), 6.67 (s, 1H), 5.81 (s, 1H), 5.68 (d, J = 3.2 Hz, 1H), 4.86 – 4.79 (m, 2H), 4.79 – 4.71 (m, 1H) ppm.

^{13}C-NMR (101 MHz, $CDCl_3$): δ = 166.3, 165.7, 165.3, 160.7, 133.94, 133.88, 133.3, 130.22, 130.15, 130.0, 129.7, 129.0. 128.79, 128.76, 128.65, 128.5, 103.3, 91.1, 84.5, 80.9, 77.4, 63.6 ppm.

Preparation of 16: Acylation of Oxazolidinone 15[30]

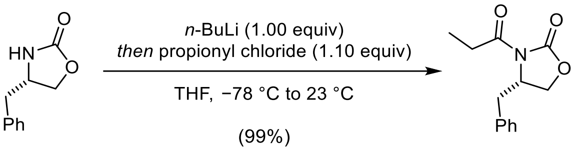

15 **16**

To a solution of oxazolidinone **15** (Combi-Blocks, Catalog No. OR-0265, 10.0 g, 56.4 mmol, 1.00 equiv) in tetrahydrofuran (225 mL) was added *n*-butyl lithium (2.3 M in hexanes, 24.5 mL, 56.4 mmol, 1.00 equiv) at −78 °C. After 20 min propionyl chloride (5.4 mL, 62.2 mmol, 1.10 equiv) was added dropwise. The reaction mixture was slowly warmed to room temperature over the course of 2.5 h. TLC analysis indicated complete consumption of the oxazolidinone **15**, and the reaction was quenched by addition of aqueous saturated ammonium chloride solution (150 mL). Most of the tetrahydrofuran was removed under reduced pressure and dichloromethane (150 mL) was added. After separation of the phases the organic layer was washed with aqueous sodium hydroxide solution (1 M, 100 mL). The aqueous layer was backextracted with dichloromethane (200 mL) and the combined organic layers were washed with brine, dried over sodium sulfate, filtered, and concentrated under reduced pressure. A short silica plug (10% EtOAc in hexanes) was sufficient to purify the product and the desired acylated product **16** (13.1 g, 56.2 mmol, 99%) was obtained as a colorless oil.

Characterization Data for 16:

The recorded spectroscopic data matched the reported ones.[30]

^{1}H-NMR (400 MHz, $CDCl_3$): δ = 7.34 (td, *J* = 7.6, 6.9, 1.3 Hz, 2H), 7.31 – 7.27 (m, 1H), 7.23 – 7.19 (m, 2H), 4.71 – 4.64 (m, 1H), 4.24 – 4.15 (m, 2H), 3.31 (dd, *J* = 13.4, 3.3 Hz, 1H), 3.05 – 2.87 (m, 2H), 2.77 (dd, *J* = 13.3, 9.6 Hz, 1H), 1.21 (t, *J* = 7.3 Hz, 3H) ppm.

^{13}C-NMR (101 MHz, $CDCl_3$): δ = 174.2, 153.7, 135.5, 129.6, 129.1, 127.5, 66.4, 55.3, 38.1, 29.4, 8.5 ppm.

Preparation of SI03: Formation of Weinreb amide SI03[24]

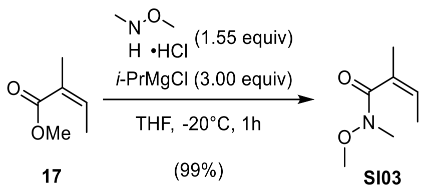

To a cooled (–20 °C) suspension of *N,O*-dimethylhydroxylamine hydrochloride (10.2 g, 105 mmol, 1.55 equiv) in tetrahydrofuran (150 mL) was added neat methyl angelate (**17**) (8.1 mL, 7.70 g, 67.5 mmol, 1.00 equiv). After 30 min of stirring, isopropyl magnesium chloride (2 M in THF, 65.0 mL, 130 mmol, 3 equiv) was added dropwise to the cloudy suspension and the mixture was stirred 30 min at –20 °C. TLC analysis (20% ethyl acetate in hexanes, $KMnO_4$) indicated complete conversion, and excess Grignard reagent was quenched by the addition of aqueous saturated ammonium chloride solution (150 mL). The precipitates were filtered and washed with ethyl acetate (50 mL). The phases were separated, and the aqueous phase extracted with ethyl acetate (2 x 100 mL). The combined organic phases were dried over magnesium sulfate, filtered, and concentrated under reduced pressure. The residue was purified by MPLC Combi-Flash column chromatography (80 g SiO_2, gradual elution: 5 to 20% EtOAc in hexanes over 35 min) affording the desired Weinreb amide **SI03** (9.61 g, 67.1 mmol, 99%) as a pale-yellow oil.

<u>Characterization Data for SI03:</u>

The recorded spectroscopic data matched the reported ones.[24]

^{1}H-NMR (400 MHz, $CDCl_3$): δ = 5.48 (tdt, *J* = 7.0, 5.4, 1.5 Hz, 1H), 3.83 – 3.52 (m, 3H), 3.24 (s, 3H), 1.90 – 1.86 (m, 3H), 1.65 (dq, *J* = 7.0, 1.6 Hz, 3H) ppm.

¹H-NMR (400 MHz, $CDCl_3$):

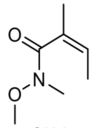

SI03

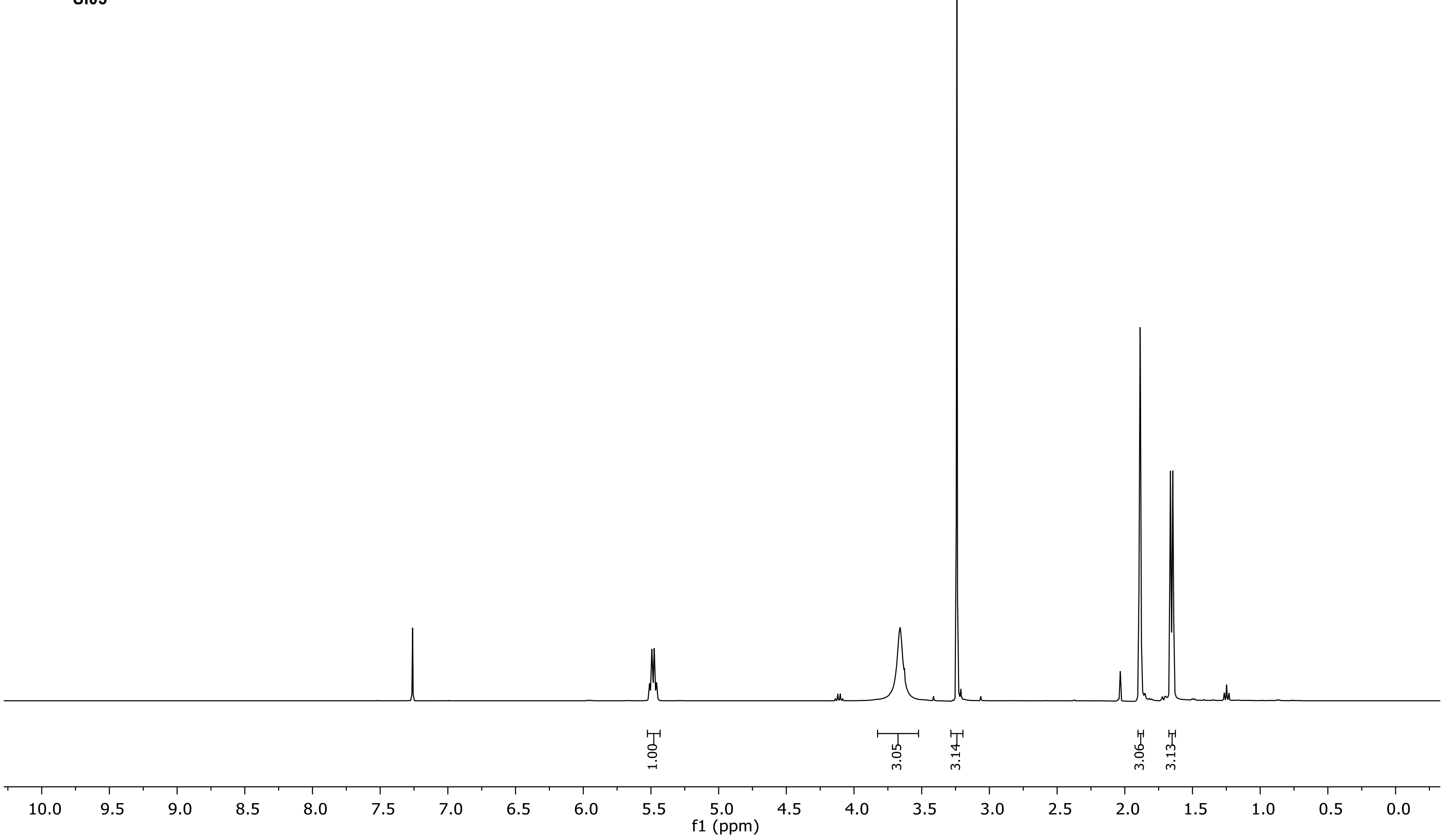

Preparation of SI05: Propargyl Grignard Addition to Weinreb Amide SI03 and CBS Reduction[24]

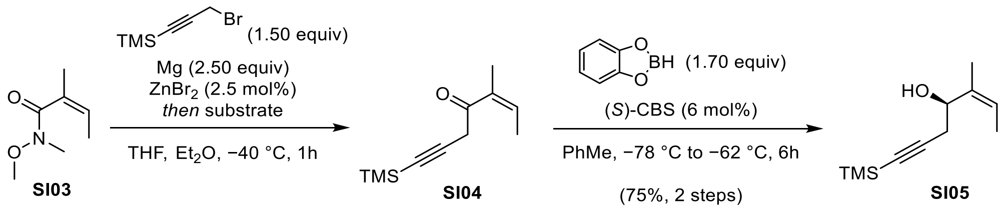

A 100 mL round bottom flask charged with a stir bar, zinc bromide (250 mg, 1.11 mmol, 2.5 mol%), and magnesium powder (2.66 g, 109 mmol, 2.50 equiv) was flame dried under vacuum. After it cooled to ambient temperature, it was flushed with argon and diethyl ether (30 mL) was added. A solution of TMS propargyl bromide (10.7 mL, 65.5 mmol, 1.50 equiv) in diethyl ether (30 mL) was added dropwise under stirring which initiated an exothermic reaction. The heterogeneous mixture was cooled to 0 °C before the addition was continued. After complete addition the mixture was stirred a further 2 h at 0 °C, resulting in a pale-yellow supernatant and pulverized Mg precipitates. This freshly prepared Grignard solution was cannulated to a solution of Weinreb amide **SI03** (6.26 g, 43.7 mmol, 1.00 equiv) in THF (190 mL) at −40 °C. The resulting reaction mixture was kept at this temperature for 1.5 h, until TLC-analysis indicated complete consumption of the Weinreb amide. Excess propargylic Grignard was quenched by the careful addition of aqueous saturated ammonium chloride solution (100 mL). After warming the resulting biphasic mixture to 23 °C, the aqueous layer was separated and extracted further with methyl *tert*-butyl ether (2 x 150 mL). The combined organics were dried over sodium sulfate, filtered, and concentrated under reduced pressure (200 mbar, 40 °C) affording the volatile enone **SI04** (8.52 g, 43.8 mmol, 99%) as a colorless liquid which was used within the subsequent reduction without further purification.

To a cooled (−78 °C, using a cryocooler) solution of enone **SI04** (8.50 g, 43.7 mmol, 1.00 equiv) in toluene (200 mL) were added sequentially the (*S*)-CBS-catalyst (1 M in toluene, 2.61 mL 2.61 mmol, 6 mol%) and catecholborane (8.2 mL, 74.3 mmol, 1.70 equiv). After 20 min the cryocooler was set to −62 °C and stirring was continued for a total reaction time of 6 h. At this point TLC-analysis (5% ethyl acetate in

hexanes, $KMnO_4$) indicated complete consumption of the enone. Methanol (50 mL) and potassium hydroxide (1g, 17.8 mmol, 0.53 eq) were added and the resulting solution was slowly warmed to ambient temperature over 2 h. Aqueous saturated ammonium chloride solution (100 mL) and water (100 mL) were added, the phases were separated, and the aqueous phase was extracted with methyl *tert*-butyl ether (2 x 120 mL). The combined organic phases were washed with water (150 mL) and aqueous saturated sodium chloride solution (150 mL), were dried over magnesium sulfate, filtered, and concentrated under reduced pressure (50 mbar, 40 °C water bath). MPLC Combi-Flash column chromatography (80 g SiO_2, gradual elution: 5 to 10% ethyl acetate in hexanes over 33 min) afforded the desired alcohol **SI05** (6.42 g, 32.7 mmol, 75%) as a pale-orange oil.

Characterization Data for SI04:

The recorded spectroscopic data matched the reported ones.[24]

^{1}H-NMR (400 MHz, $CDCl_3$): δ = 5.91 (qq, *J* = 7.2, 1.5 Hz, 1H), 3.46 (s, 2H), 1.97 (q, *J* = 1.5 Hz, 3H), 1.90 (dq, *J* = 7.2, 1.5 Hz, 3H), 0.17 (s, 9H) ppm.

Characterization Data for SI05:

The recorded spectroscopic data matched the reported ones.[24]

^{1}H-NMR (400 MHz, $CDCl_3$): δ = 5.44 – 5.29 (m, 1H), 4.80 – 4.68 (m, 1H), 2.54 (dd, *J* = 16.7, 8.0 Hz, 1H), 2.39 (dd, *J* = 16.7, 5.6 Hz, 1H), 1.95 (s, 1H), 1.70 (q, *J* = 1.5 Hz, 3H), 1.64 (dq, *J* = 7.1, 1.6 Hz, 3H), 0.15 (s, 9H) ppm.

^{13}C-NMR (101 MHz, $CDCl_3$): δ = 135.8, 122.8, 103.4, 87.4, 67.9, 27.0, 17.4, 13.2, 0.2 ppm.

¹H-NMR (400 MHz, $CDCl_3$):

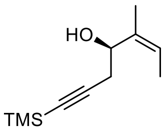

SI05

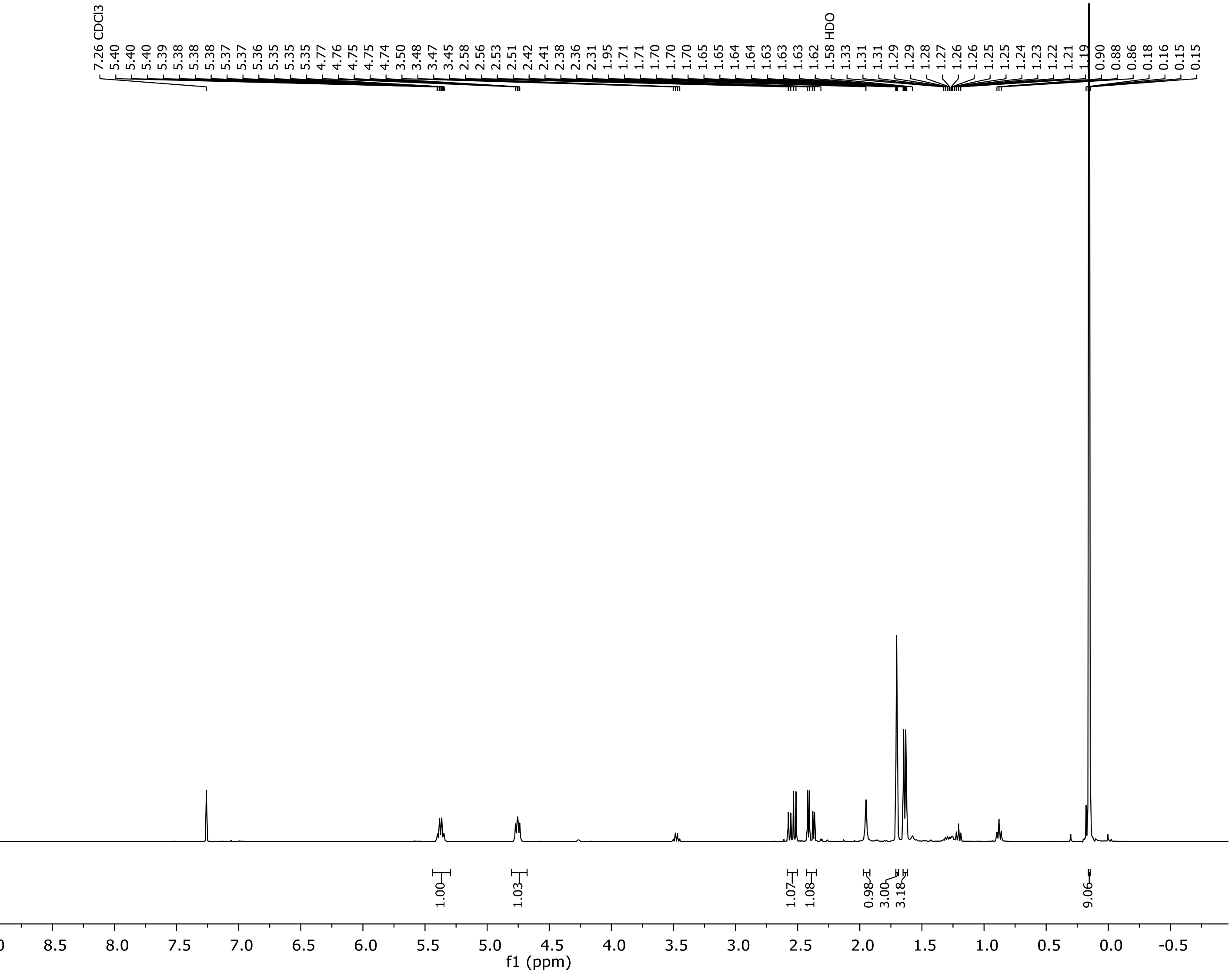

^{13}C-NMR (101 MHz, $CDCl_3$):

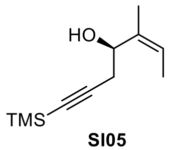

SI05

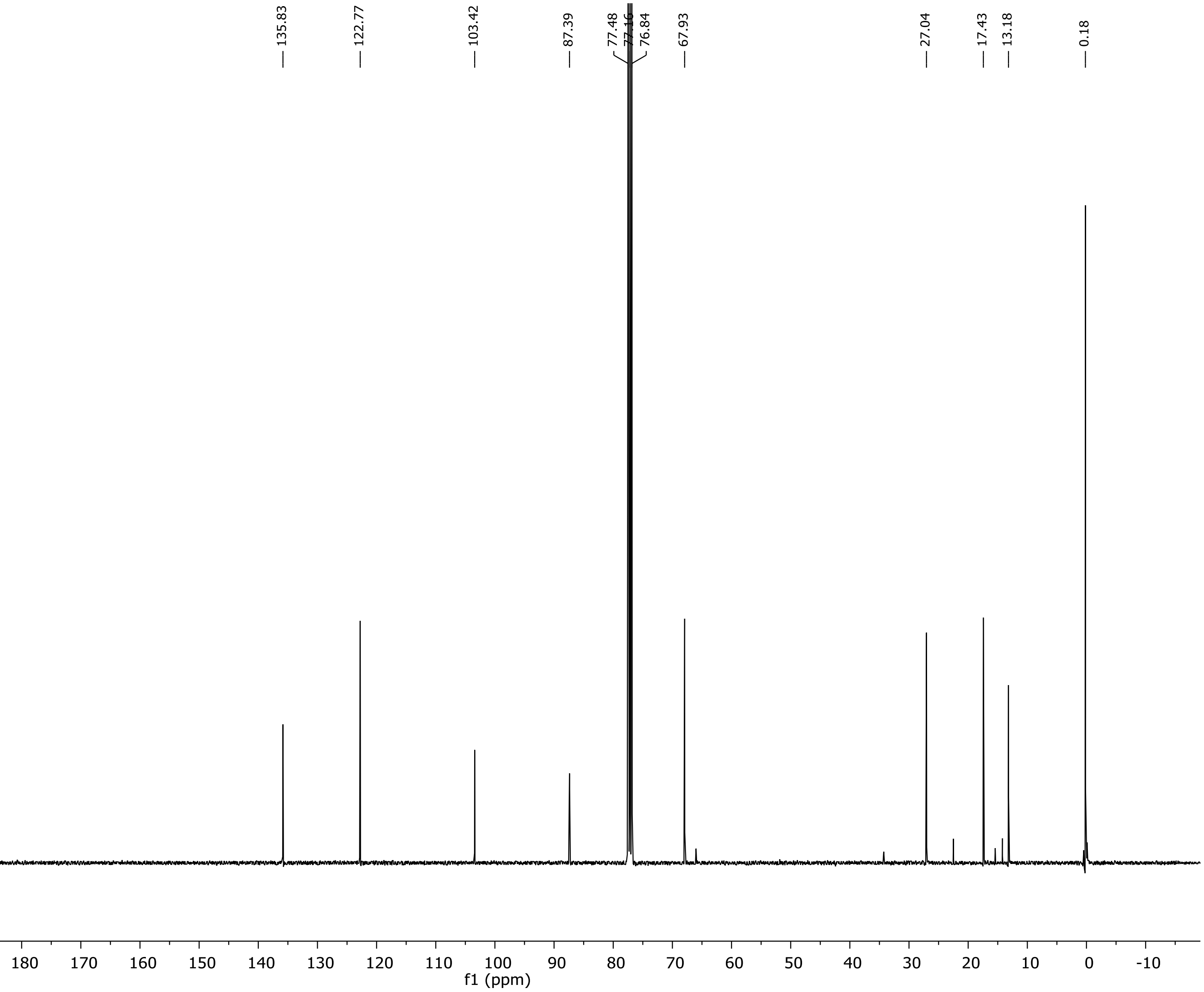

Preparation of SI07: Formation of Mosher's Ester SI07

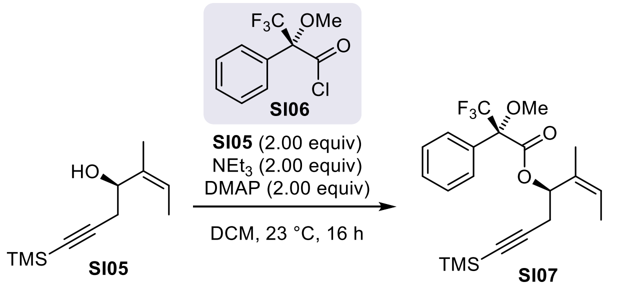

To a cooled (0 °C) solution of alcohol **SI05** (5.0 mg, 25.5 µmol, 1.00 equiv) in dichloromethane (250 µL) was added acid chloride **SI06** (10 µL, 53.4 µmol, 2.10 equiv), triethylamine (7 µL, 50.3 µmol, 1.98 equiv) and DMAP (6.2 mg, 50.7 µmol, 1.99 equiv), sequentially. After complete addition, the cooling bath was removed and stirring was continued until TLC analysis indicated full consumption of the alcohol starting material (typically 10 h to 16 h). Aqueous saturated sodium bicarbonate solution (2 mL) and ethyl acetate (2 mL) were added, the phases were separated, and the aqueous phase was extracted with ethyl acetate (2 x 2 mL). The combined organics were washed with brine (2 mL), dried over anhydrous sodium sulfate, filtered, and concentrated to afford the crude ester **SI07** as a colorless, amorphous solid. NMR analysis of the crude product measured a diastereomeric ratio of 95:5, indicating an ee of 90% for the Corey–Itsuno reduction.

NOTE: The crude product was not purified further but the obtained mixture of diastereomers was used as obtained to measure the diastereomeric ratio of products.

[1]**H-NMR** (400 MHz, $CDCl_3$):

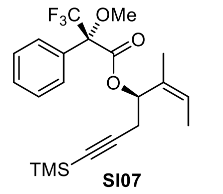

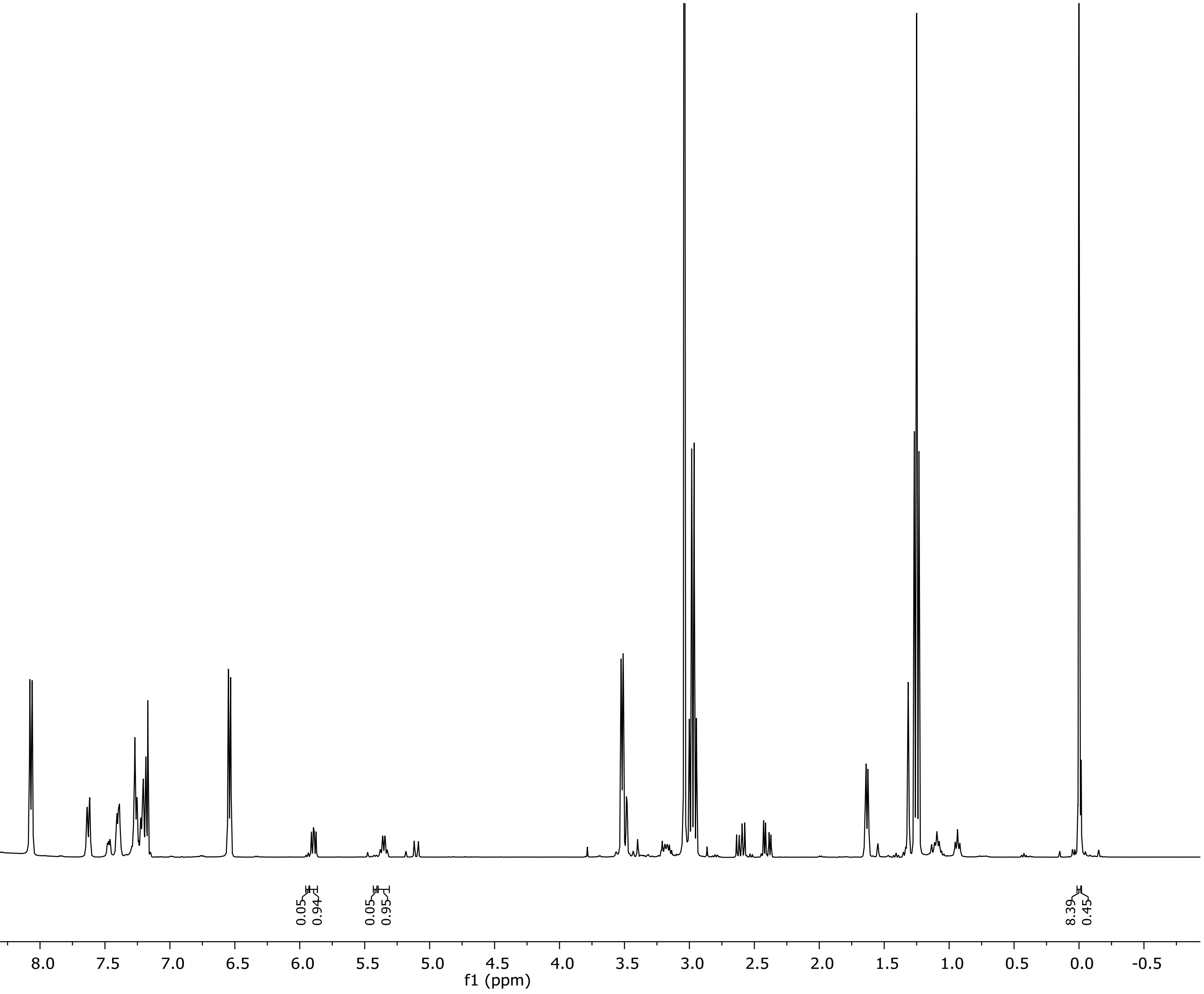

Preparation of SI08: Desilylation of TMS-alkyne SI07[24]

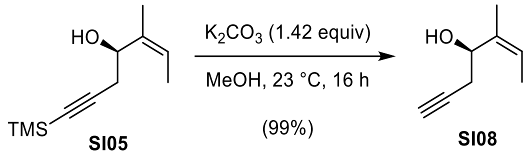

Potassium carbonate (7.70 g, 55.7 mmol, 1.42 equiv) was added to a solution of TMS-alkyne **SI05** (7.70 g, 39.2 mmol, 1.00 equiv) in methanol (150 mL) at ambient temperature. TLC-analysis (20% ethyl acetate in hexanes, $KMnO_4$) indicated complete consumption of the starting material after 16 h of reaction time. The mixture was diluted with water (50 mL) and most of the methanol was removed under reduced pressure. The aqueous phase was extracted with diethyl ether (3 x 80 mL), the combined organic phases were washed with aqueous saturated sodium chloride solution, dried over sodium sulfate, filtrated, and concentrated (200 mbar, 40 °C). The crude product was purified over SiO_2 using MPLC Combi-Flash column chromatography (80 g silica, gradual elution, 0 to 20% diethyl ether in pentanes over 30 min) affording the volatile terminal alkyne **SI08** (4.85 g, 39.1 mmol, 99%) as a colorless liquid.

__Characterization Data for SI08:__

The recorded spectroscopic data matched the reported ones.[24]

^{1}H-NMR (400 MHz, $CDCl_3$): δ = 5.40 (q, *J* = 7.0 Hz, 1H), 4.79 (ddd, *J* = 8.4, 5.5, 3.3 Hz, 1H), 2.53 (ddd, *J* = 16.6, 8.4, 2.7 Hz, 1H), 2.36 (ddd, *J* = 16.7, 5.6, 2.7 Hz, 1H), 2.04 (t, *J* = 2.7 Hz, 1H), 1.84 (d, *J* = 3.3 Hz, 1H), 1.72 (s, 3H), 1.65 (d, *J* = 7.0, 3H) ppm.

^{13}C-NMR (101 MHz, $CDCl_3$): δ = 135.6, 123.0, 81.0, 70.3, 67.8, 25.3, 17.2, 13.0 ppm.

^{1}H-NMR (400 MHz, $CDCl_3$):

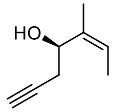

SI08

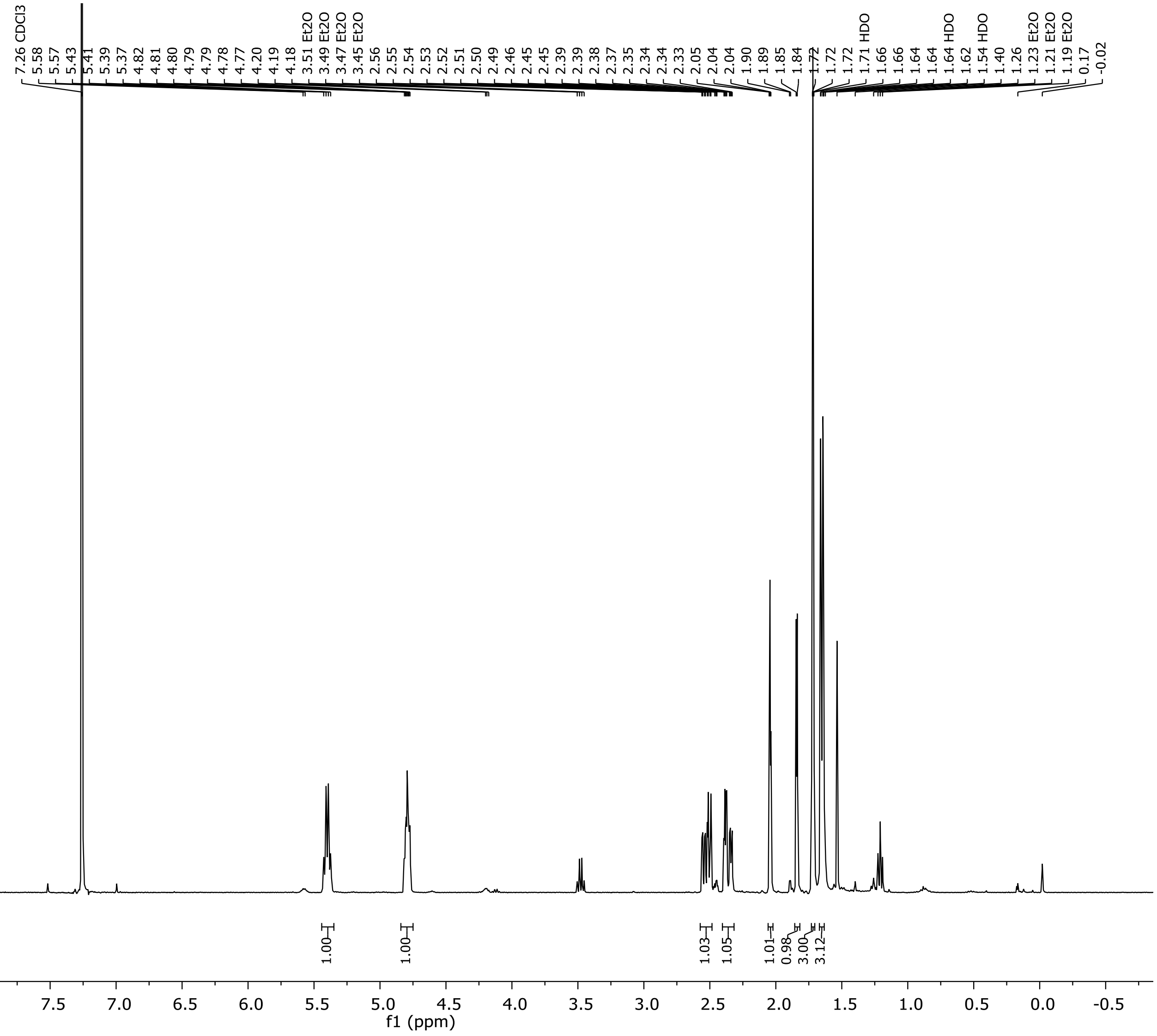

^{13}C-NMR (101 MHz, $CDCl_3$):

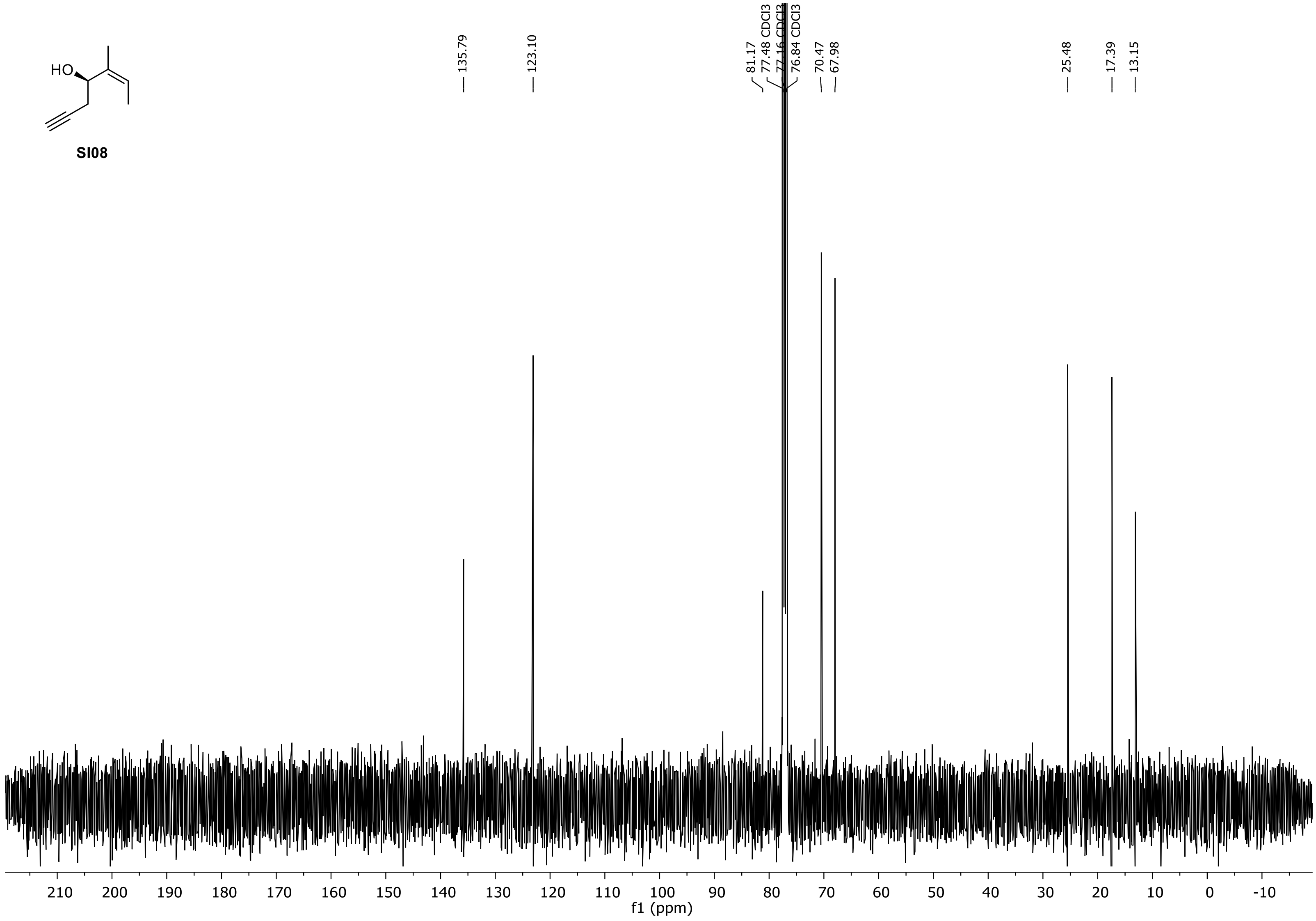

SI08

Preparation of SI09: TBS-Protection of Alcohol SI08[24]

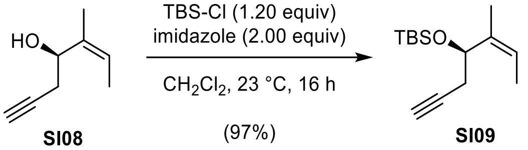

tert-Butyldimethylsilyl chloride (4.85 g, 32.2 mmol, 1.20 equiv) was added to a solution of alcohol **SI08** (3.32 g, 26.8 mmol, 1.00 equiv) and imidazole (3.65 g, 53.6 mmol, 2.00 equiv) in dichloromethane (100 ml) at 23 °C. After stirring for 12 h, TLC analysis (20 % ethyl acetate in hexanes) indicated complete conversion and aqueous saturated sodium bicarbonate solution (100 mL) was added. The phases were separated, and the aqueous phase was further extracted with dichloromethane (2 x 100 mL). The combined organic layers were washed with aqueous saturated sodium chloride solution, were further dried over anhydrous magnesium sulfate, filtered, and concentrated under reduced pressure (250 mbar, 40 °C water bath). The crude product was purified over SiO_2 using MPLC Combi-Flash column chromatography (80 g silica, gradual elution, 0 to 15% diethyl ether in pentanes over 33 min) to afford TBS ether **SI09** (6.20 g, 26.0 mmol, 97%) as a colorless volatile liquid.

<u>**Characterization Data for SI09:**</u>

The recorded spectroscopic data matched the reported ones.[24]

^{1}H-NMR (400 MHz, $CDCl_3$): δ = 5.30 (q, *J* = 6.8 Hz, 1H), 4.75 (t, *J* = 6.9 Hz, 1H), 2.44 (ddd, *J* = 16.6, 6.9f, 2.7 Hz, 1H), 2.32 (ddd, *J* = 16.6, 6.9, 2.7 Hz, 1H), 1.93 (t, *J* = 2.7 Hz, 1H), 1.67 – 1.62 (m, 6H), 0.89 (s, 9H), 0.08 (s, 3H), 0.03 (s, 3H) ppm.

^{13}C-NMR (101 MHz, $CDCl_3$): δ = 137.1, 121.0, 81.9, 69.4, 68.8, 26.3, 25.9, 18.4, 17.3, 13.3, −4.8 ppm.

¹H-NMR (400 MHz, $CDCl_3$):

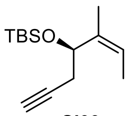

SI09

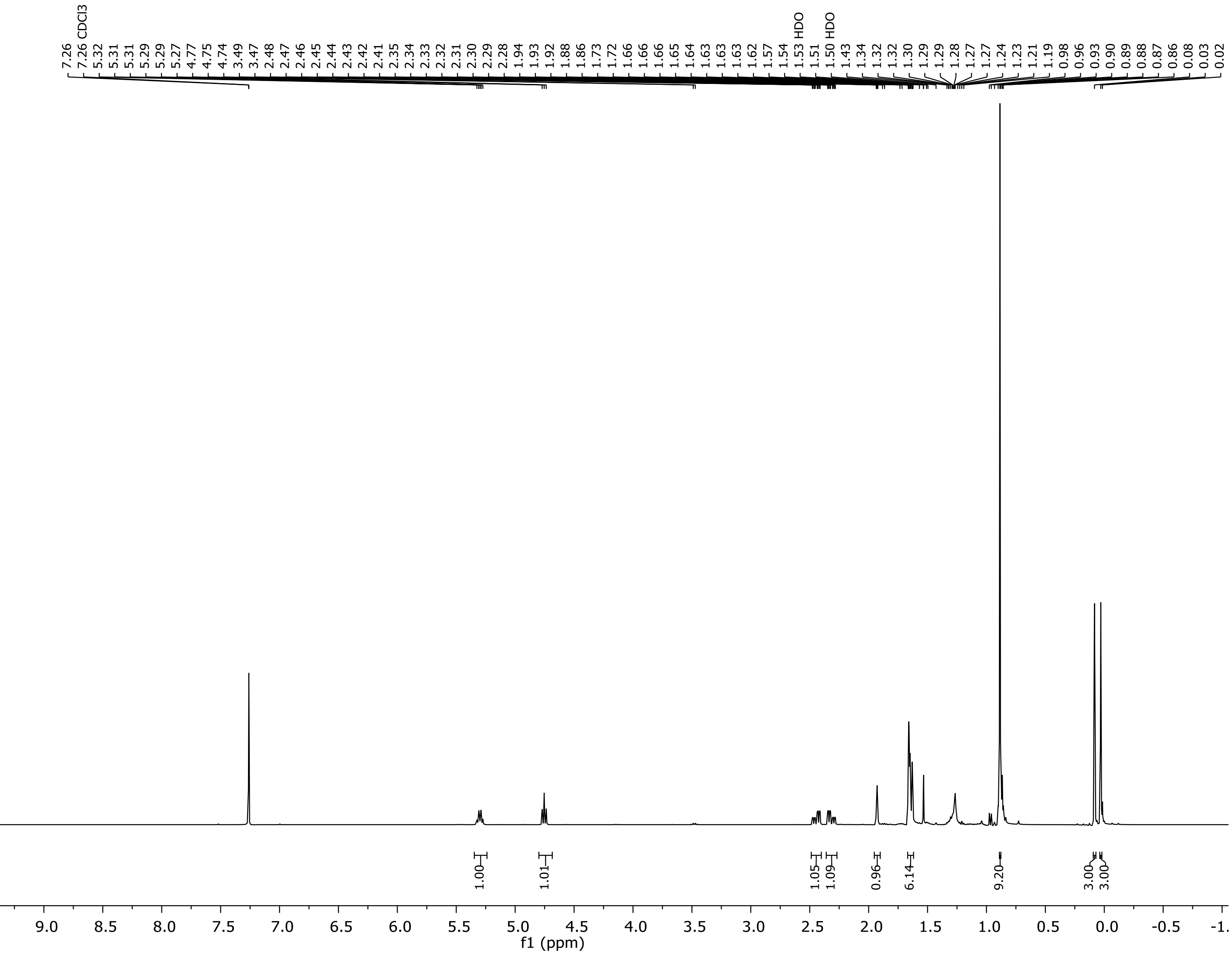

¹³C-NMR (101 MHz, $CDCl_3$):

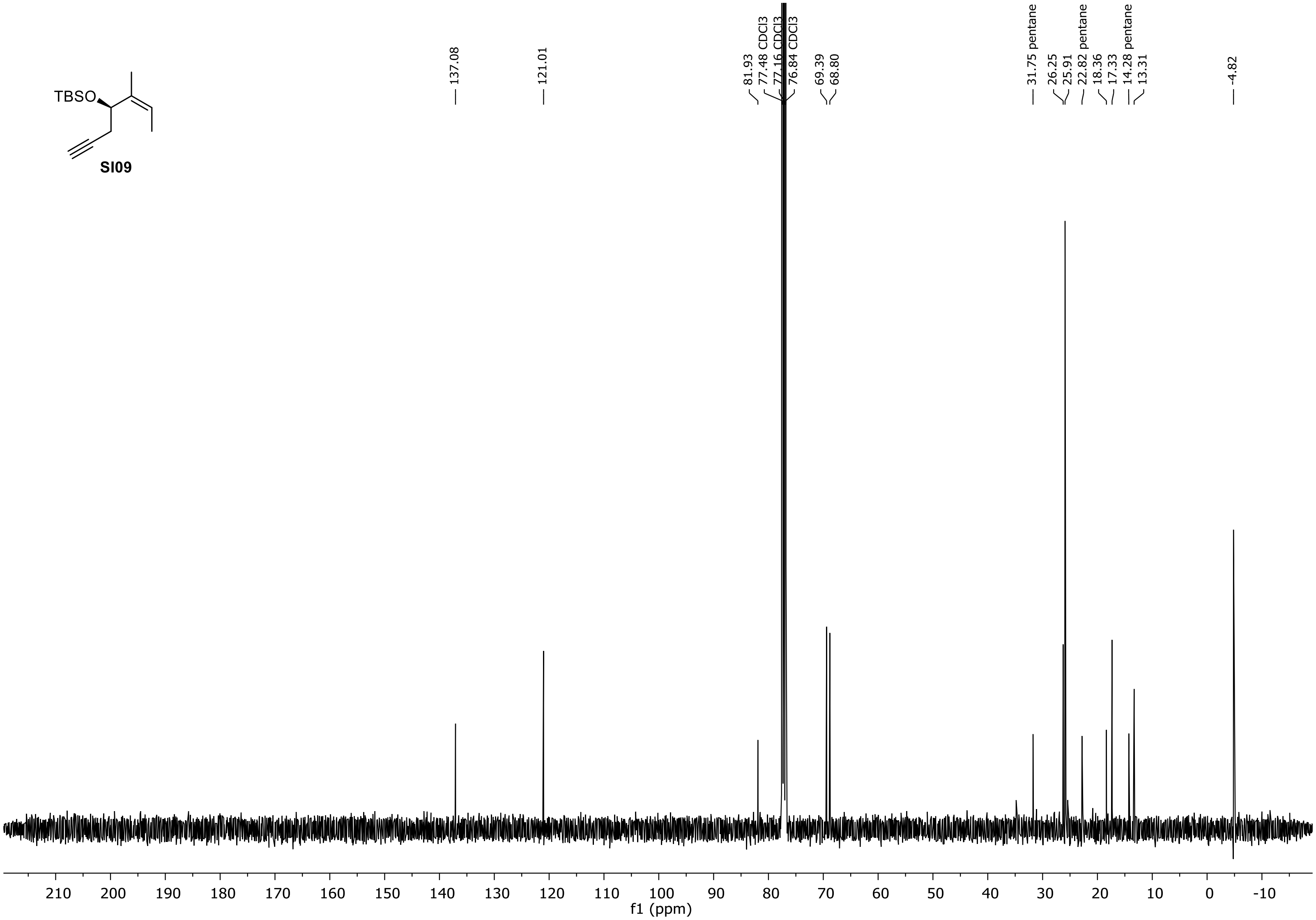

Preparation of 27: Methylation of Terminal Alkyne SI09[24]

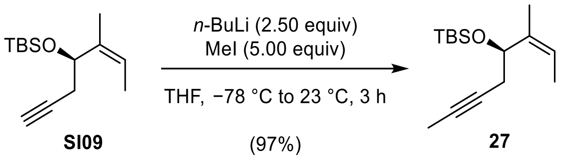

To a solution of the alkyne **SI09** (6.20 g, 26.0 mmol, 1.00 equiv) in THF (180 mL) was added *n*-butyllithium (2.0 M in cyclohexane/hexane,32.5 mL, 65.0 mmol, 2.5 equiv) dropwise at −78 °C. After 1 h methyl iodide (8.10 mL, 130 mmol, 5.00 equiv) was added and the solution was slowly warmed to ambient temperature (23 °C) at which it was stirred further 3 h until TLC-analysis (10% ethyl acetate in hexanes, CAM) indicated complete conversion. Excess *n*-BuLi was quenched by the addition of aqueous saturated ammonium chloride solution (150 mL). The phases were separated, and the aqueous phase was extracted with pentanes (2 x 100 mL). The combined organic layers were washed with aqueous saturated sodium chloride solution, dried over magnesium sulfate, filtered, and concentrated under reduced pressure (200 mbar, 40 °C water bath). The afforded crude product was purified over silica gel by MPLC Combi-Flash column chromatography (24 g SiO_2, 0 to 20% diethyl ether in pentane) affording the desired methylated product **27** (6.34 g, 25.1 mmol, 97%) as a colorless liquid.

Characterization Data for 27:

The recorded spectroscopic data matched the reported ones.[24]

^{1}H-NMR (400 MHz, $CDCl_3$): δ = 5.30 – 5.23 (m, 1H), 4.72 – 4.66 (m, 1H), 2.38 (ddq, *J* = 16.4, 7.5, 2.6 Hz, 1H), 2.22 (ddq, *J* = 16.4, 6.7, 2.6 Hz, 1H), 1.76 (t, *J* = 2.6 Hz, 3H), 1.65 – 1.61 (m, 6H), 0.89 (s, 9H), 0.08 (s, 3H), 0.03 (s, 3H) ppm.

^{13}C-NMR (101 MHz, $CDCl_3$): δ = 137.4, 120.4, 76.60, 76.58, 69.3, 26.4, 25.8, 18.3, 17.3, 13.1, 3.5, −4.97, −5.00 ppm.

¹H-NMR (400 MHz, $CDCl_3$):

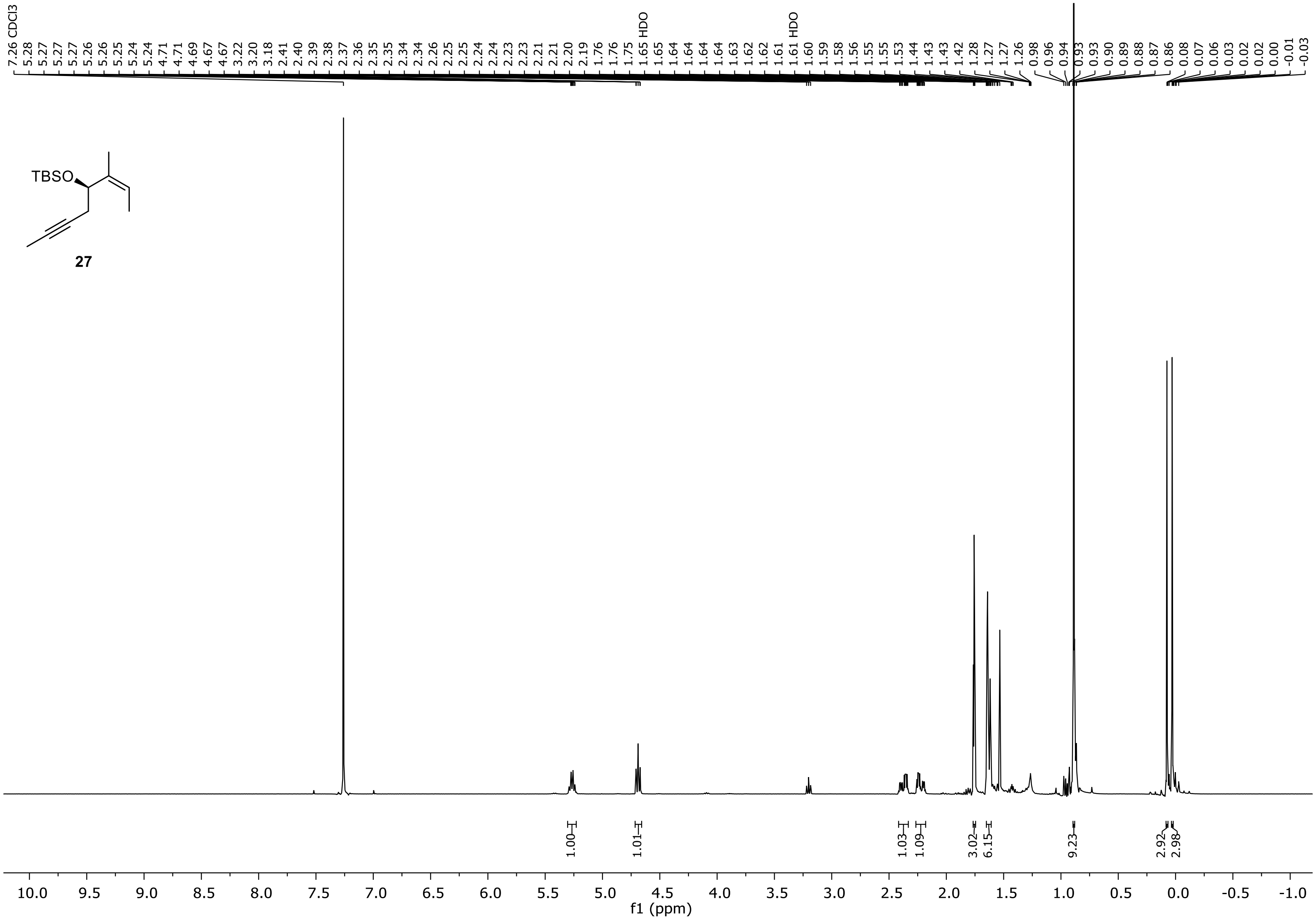

^{13}C-NMR (101 MHz, $CDCl_3$):

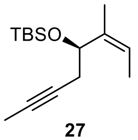

27

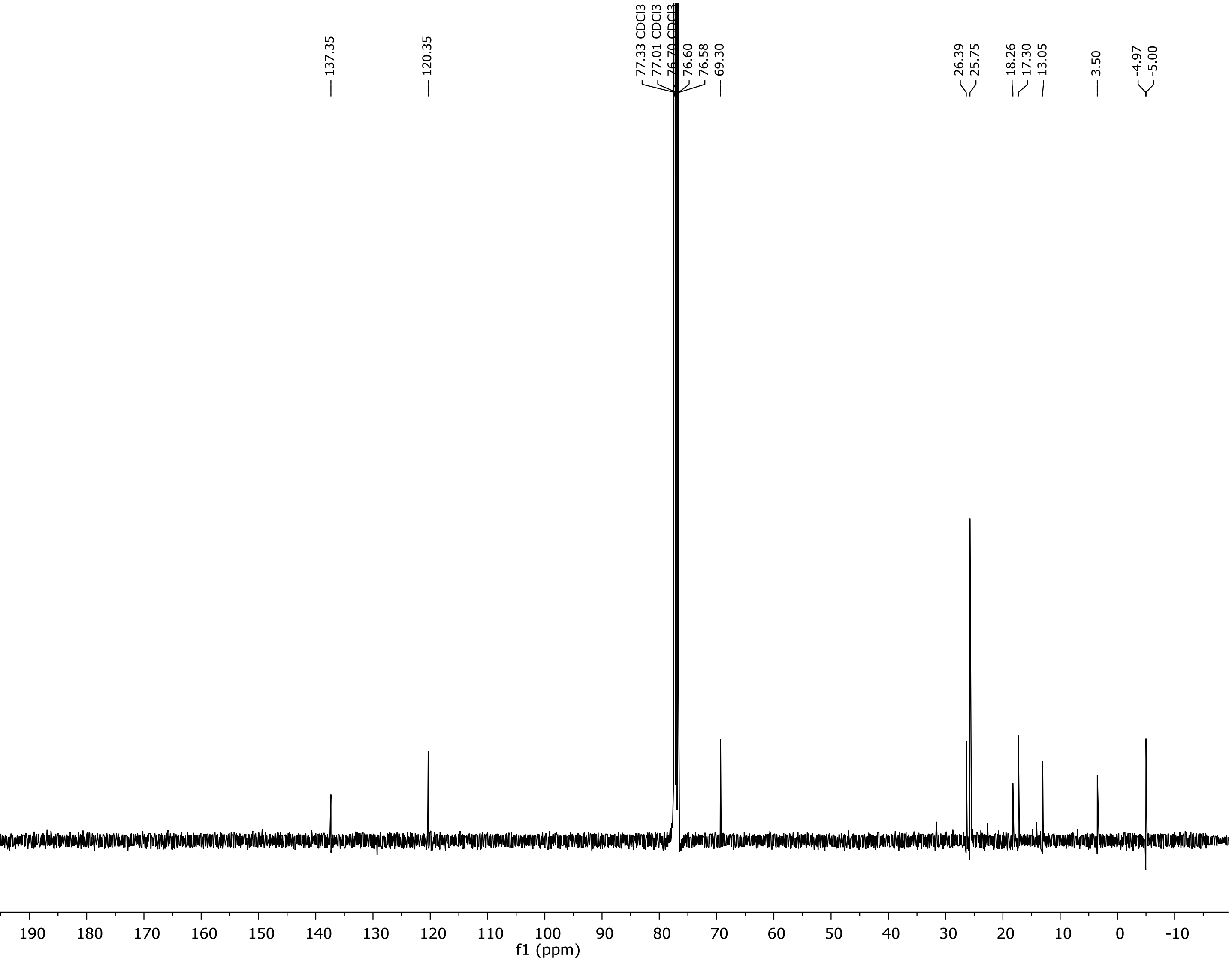

Preparation of 19: Hydrozirconation, Iodination and Desilylation of Alkyne 27

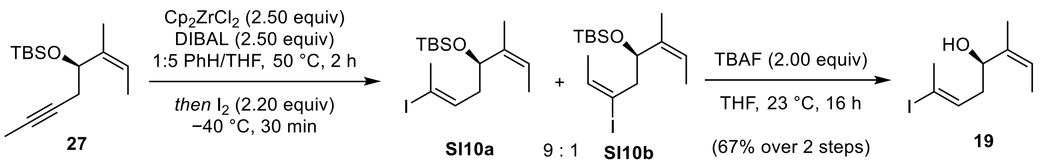

In a 20 mL Schlenck tube, a mixture of zirconocene dichloride (470 mg, 1.61 mmol, 2.51 equiv) in THF (1.5 mL) was cooled to 0 °C and a solution of DIBAL (1 M in toluene, 1.6 mL, 1.60 mmol, 2.49 equiv) was added under an argon atmosphere. The resulting suspension was stirred for 30 min at 0 °C, before a solution of alkyne **27** (162 mg, 0.642 mmol, 1.00 equiv) in THF (1 mL) and benzene (0.5 mL) was added. The mixture was warmed to room temperature, then heated to 50 °C using a heating mantle, and stirring was continued for 1 h. It was then cooled to −40 °C, and a solution of iodine (358 mg, 1.41 mmol, 2.20 equiv) in THF (2.5 mL) was added. The reaction mixture was allowed to warm to room temperature (23 °C), diluted with pentane (10 mL), and filtered through a short pad of silica. The product containing fractions were concentrated and the vinyl iodides **SI10a** and **SI10b** (244 mg, 0.641 mmol, 99%) were obtained as a 9:1 mixture of regioisomers which were inseparable and used for the subsequent desilylation step without further purification.

To a solution of silyl ethers **SI10a** and **SI10b** (244 mg, 0.641 mmol, 1.00 equiv) in THF (6 mL) was added a TBAF-solution (1 M in THF, 2.6 mL, 2.60 mmol, 4.05 equiv) at 23 °C. The reaction mixture was stirred for 16 h until TLC analysis (5% ethyl acetate in hexanes, CAM) indicated complete conversion. Aqueous saturated sodium bicarbonate solution (10 mL) and diethyl ether (10 mL) were added, and the phases were separated. The aqueous phase was extracted further using diethyl ether (2 x 10 mL), the combined organic phases were washed with brine, dried over anhydrous sodium sulfate, filtered, and concentrated (150 mbar, 40 °C water bath). The crude product was purified over silica gel by MPLC Combi-

Flash column chromatography (24 g silica, gradual elution, 0 to 15% diethyl ether in pentanes over 25 min) which afforded allylic alcohol **19** (115 mg, 0.432 mmol, 67%) (NOTE 1) as a colorless volatile liquid.

NOTE 1: The product was kept in an amber glass vial, or a glass vial wrapped in aluminum foil for storage within the freezer in order to prevent photoisomerization or deiodination.

Characterization Data for SI10a+SI10b:

R*f* (100% hexanes) = 0.59 (CAM).

^{1}H-NMR (400 MHz, $CDCl_3$): δ = 6.16 – 6.09 (m, 1H), 5.27 – 5.19 (m, 1H), 4.53 (ddd, *J* = 7.9, 5.9, 0.8 Hz, 1H), 2.38 (dt, *J* = 1.6, 0.8 Hz, 3H), 2.33 – 2.26 (m, 1H), 2.10 – 2.02 (m, 1H), 1.65 (q, *J* = 1.5 Hz, 3H), 1.60 – 1.57 (m, 3H), 0.88 (d, *J* = 0.7 Hz, 9H), 0.05 (s, 3H), 0.00 (s, 3H) ppm.

Characterization Data for 19:

R*f* (10% EtOAc in hexanes) = 0.28 (UV, CAM).

$[\boldsymbol{\alpha}]_{\mathbf{D}}^{\mathbf{26}}$ = +13.0° (c = 2, $CHCl_3$).

^{1}H-NMR (400 MHz, $CDCl_3$): δ = 6.19 – 6.12 (m, 1H), 5.37 (qd, *J* = 7.2, 1.5 Hz, 1H), 4.68 – 4.59 (m, 1H), 2.44 – 2.35 (m, 4H), 2.16 (dtt, *J* = 14.4, 7.2, 1.0 Hz, 1H), 1.70 (t, *J* = 1.5 Hz, 3H), 1.63 – 1.60 (m, 3H) ppm.

^{13}C-NMR (101 MHz, $CDCl_3$): δ = 137.1, 136.4, 122.5, 96.1, 68.5, 36.1, 27.9, 17.5, 13.1 ppm.

IR (Diamond-ATR, neat): $\tilde{\nu}$ = 3360 (br, m), 2957 (s), 2919 (s), 1637 (w), 1453 (m), 1433 (m), 1376 (m), 1260 (w), 1054 (s), 1022 (s), 825 (w) cm^{-1}.

HRMS (ESI-TOF) m/z: $[M+H]^+$ calcd. for $C_9H_{16}IO^+$: 267.0240; found: 267.0261.

¹H-NMR (400 MHz, $CDCl_3$):

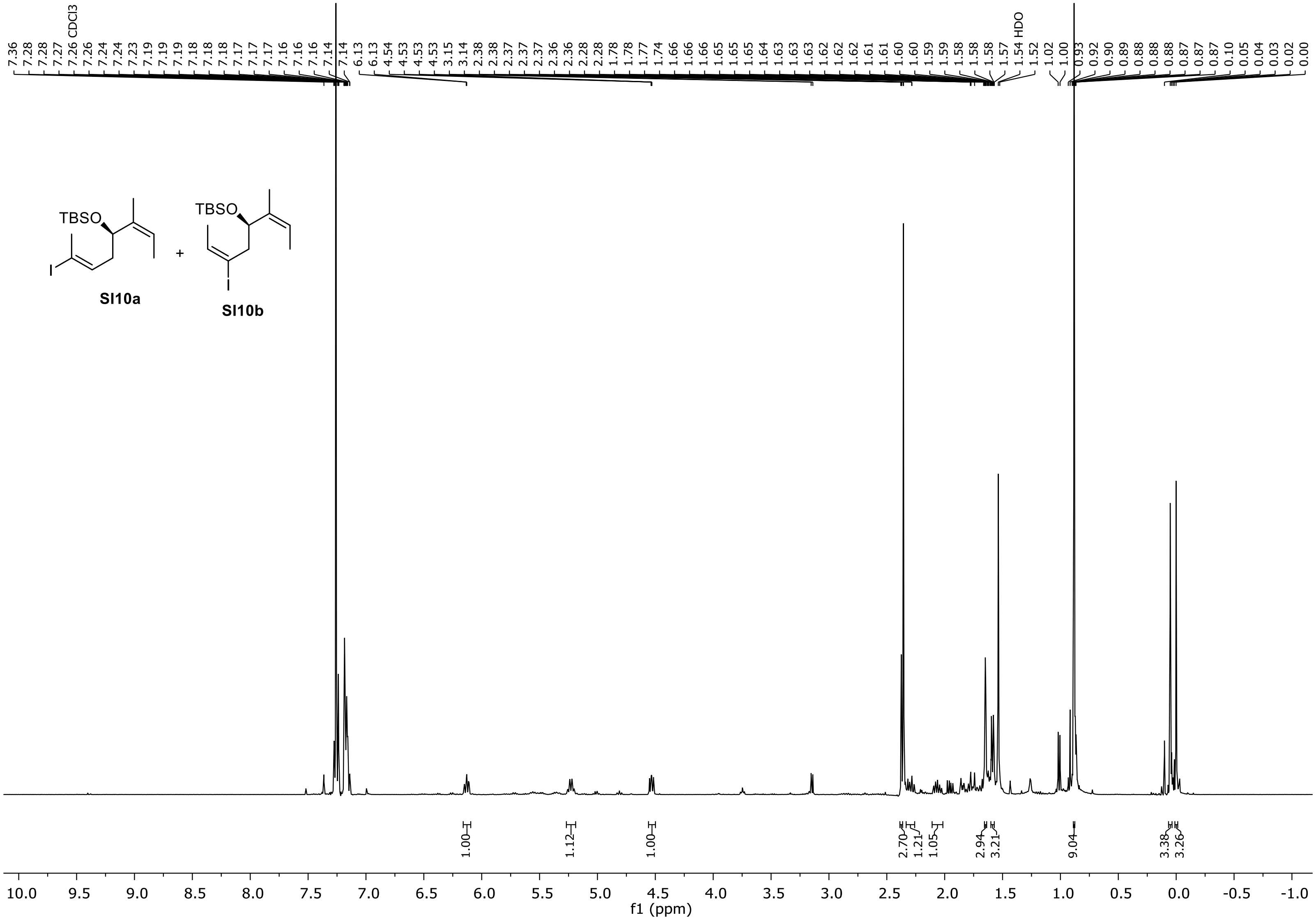

^{1}H-NMR (400 MHz, $CDCl_3$):

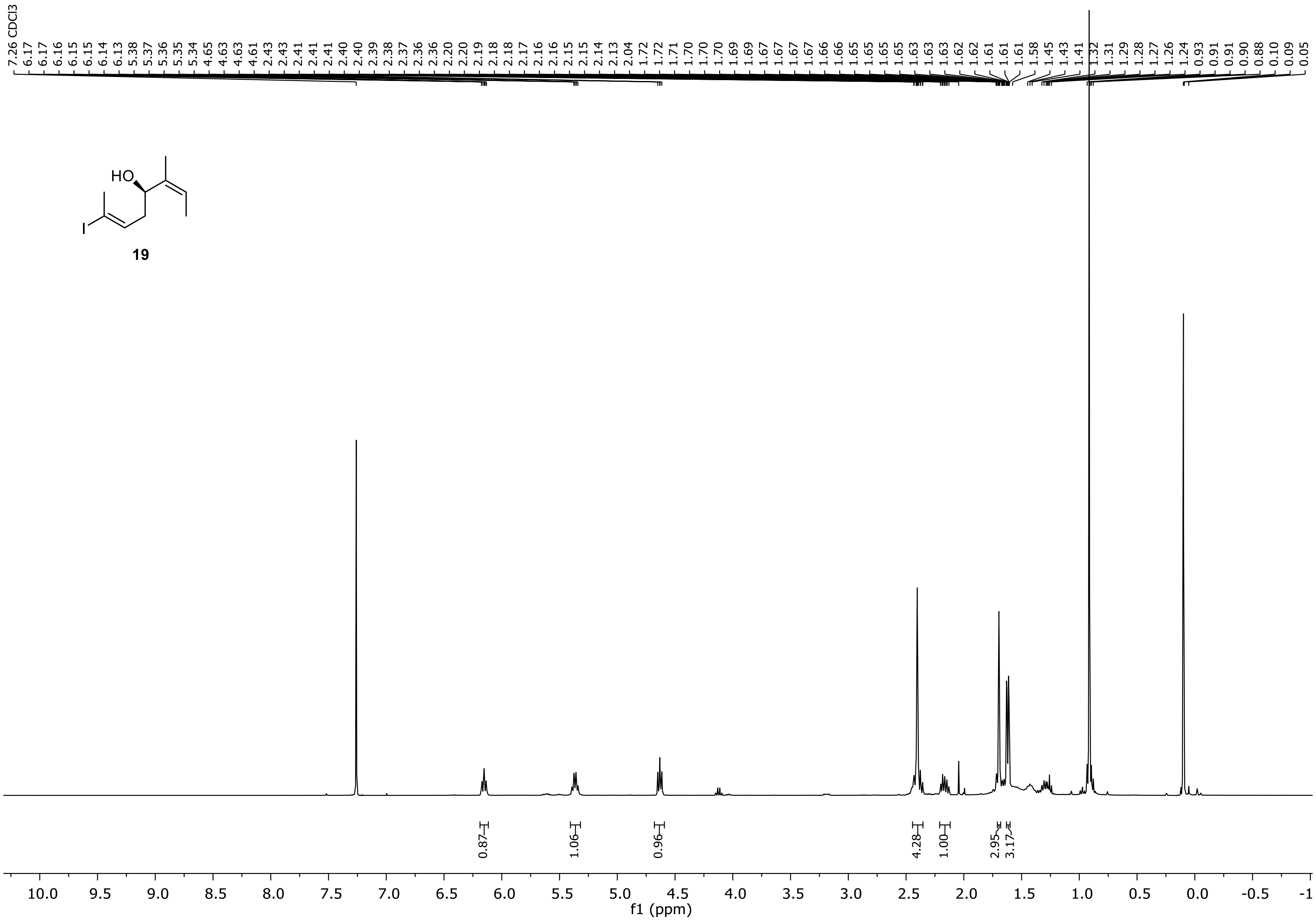

^{13}C-NMR (101 MHz, $CDCl_3$):

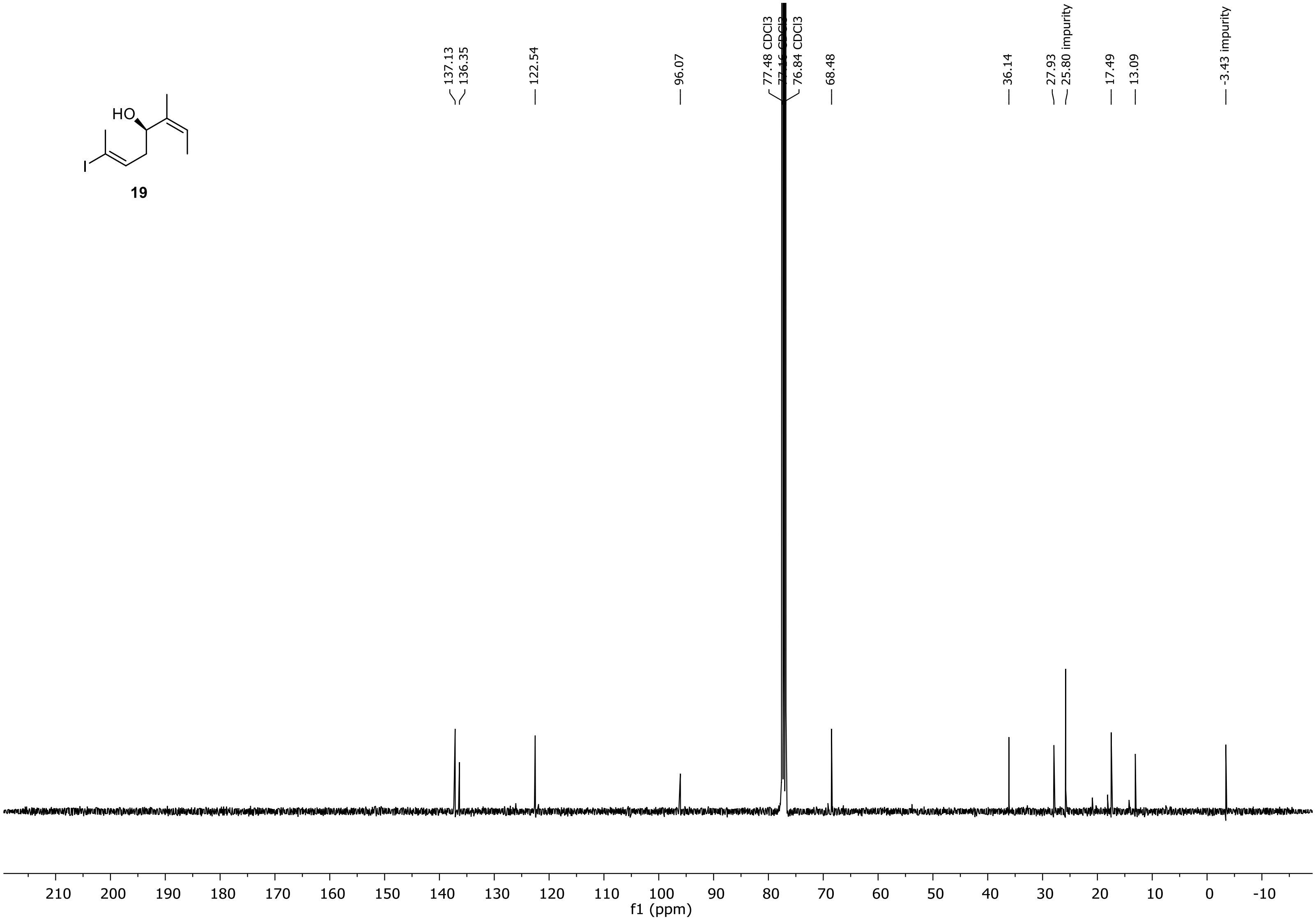

Preparation of SI11: Sharpless Epoxidation of Divinyl Carbinol 20[29]

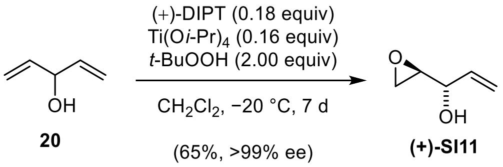

A 1 L single necked round bottom flask (24/40 joint) is charged with a 4 cm Teflon-coated egg-shaped stir bar and powdered 4 Å molecular sieves (10.0 g). The flask is placed together with a 250 mL graduated cylinder containing a 24/40 joint in a 160 °C oven and left over night. After 12 h the flask and graduate cylinder are taken out of the oven and are sealed immediately with a rubber septum. Both systems are connected to high vacuum (≤0.1 mbar) by use of a needle adapter, connected to a Schlenk line and are allowed to cool to ambient temperature before refilling with an inert nitrogen atmosphere. Using the graduate cylinder dichloromethane (240 mL, 0.5 M) is cannulated to the reaction flask. The round bottom flask containing the resulting white suspension is placed in an ice/salt bath (1:1, 800 g ice + 800 g salt) with an external temperature reading of the resulting slurry of −15 °C to −16 °C. While stirring (300 rpm) (+)-diisopropyl L-tartrate (4.5 mL, 21.4 mmol, 0.18 equiv) is added to the reaction flask. Then freshly distilled titanium tetraisopropoxide (5.7 mL, 19.0 mmol, 0.16 equiv) is added, followed by the dropwise addition of a *tert*-butyl hydroperoxide solution (5.5 M in decane, 43.2 mL, 238 mmol, 2.00 equiv) over the course of 5 minutes. The reaction mixture is stirred for 15 min before the neat divinyl carbinol (**20**) (11.6 mL, 119 mmol, 1.00 equiv) is added over the course of 3 min, turning the before colorless suspension deep orange. The needle adapter is removed from the reaction flask, the septum is sealed with electrical tape and the flask is placed in a −20 °C freezer for 1 week. After 7 days the reaction flask is taken out of the freezer and the electrical tape and septum is removed. TLC analysis indicated complete consumption of starting material. Acetone (200 mL), water (20 mL) and citric acid (2.52 g) are added, and the mixture is stirred for 1 h (350 rpm) while warming to ambient temperature (21 °C). The reaction mixture is filtered over celite using

a 9 cm diameter fritted Büchner funnel into a 2 L round bottom flask. The reaction flask and celite pad are washed with dichloromethane (3 x 60 mL) and the filtrate is concentrated under reduced pressure using a rotary evaporator (100 mbar, 40 °C water bath) (NOTE 1) affording a clear bronze colored crude oil. The crude product was purified by silica gel column chromatography (7cm diameter column, 200 g of SiO_2, 1 L of 10% diethyl ether in hexanes prerun, collected In 2 500 mL Erlenmeyer flasks,, then 2 L of 50% diethyl ether in hexanes, collected in 50 mL test tubes (38 fractions), TLC analysis (40% ethyl acetate in hexanes, stained with potassium permanganate stain) indicated product in fractions 13–35) (NOTE 2) affording 14.2 g of the desired product, contaminated with residual, copolar diisopropyl tatrate as a clear colorless oil. Further purification can be achieved by fractional distillation. For this, the product obtained from column chromatography is transferred into a 50 mL round bottom flask. A 2 cm Teflon-coated, egg-shaped stir bar was added to the round bottom flask and a short-path distillation set-up was assembled. An oil bath was used as the heating source. The product is distilled under vacuum and collected in one fraction (20 mbar, 70-72 °C), affording the pure epoxide **(+)-SI11** as a clear, colorless liquid (7.54 g, 75.3 mmol, 65%). The enantiomeric excess (ee) was determined by chiral GC analysis and assessed to be >99.9% (NOTE 3).

NOTE 1: The product is highly volatile. Despite measuring a boiling point of 72 °C at 20 mbar, significant amounts of products were detected in the receiving flask of the rotary evaporator if lower pressures than 100 mbar or higher water bath temperatures than 40 °C were applied. The evaporation time should be kept minimal.

NOTE 2: This initial silica plug was necessary to separate the *tert*-butanol side product from the crude mixture as it formed azeotropic mixtures with the product in the subsequent distillation step. If *tert*-butanol impurities do not impact subsequent transformations, the column chromatography step can be omitted. Attempts of separating the diisopropyl tartrate by column chromatography using larger amounts of silica and slower gradients of the eluent were unsuccessful.

NOTE 3: The enantiomeric excess was determined by chiral gas chromatography (GC) using a Shimadzu GC-2010 Plus, equipped with a chiral BETA DEXTM 325 fused silica capillary column (30 m x 0.25 mm x 0.25 μm film thickness). The temperature gradient used was as follows: 3 minutes of equilibration at 25 °C, followed by linear increase to 70 °C over 90 minutes (0.5 °C/min). In the scalemic mixture prepared (3.2 mg of **(+)-SI11** and 2.8 mg of **(−)-SI11**), separation was observed when this temperature gradient was applied, unfortunately the method resulted in significant tailing of both peaks and created an overlap. This makes a quantitative analysis of the ee for the (+)-enantiomer impossible. The (−)-enantiomer, however, can be assumed to have an ee of >99.9% as a corresponding peak for **(+)-SI11** was below the detection limit of the instrument. Based on chemical logic, the (+)-enantiomer should have the same enantiomeric purity.

Characterization Data for (+)-SI11:

R$_f$ (40% EtOAc in haxanes) = 0.26 ($KMnO_4$).

$[\alpha]_D^{22}$ = +60.1° (c = 1, $CHCl_3$).

bp = 70–72 °C at 20 mbar.

^{1}H-NMR (400 MHz, $CDCl_3$): δ = 5.92 – 5.80 (m, 1H), 5.41 (dq, *J* = 17.3, 1.3 Hz, 1H), 5.28 (dt, *J* = 10.4, 1.3 Hz, 1H), 4.38 – 4.34 (m, 1H), 3.14 – 3.09 (m, 1H), 2.82 (dd, *J* = 5.0, 2.8 Hz, 1H), 2.76 (ddd, *J* = 5.0, 3.9, 0.9 Hz, 1H), 1.88 (d, *J* = 2.7 Hz, 1H) ppm.

^{13}C-NMR (101 MHz, $CDCl_3$): δ = 135.6, 117.9, 70.3, 54.0, 43.6 ppm.

IR (Diamond-ATR, neat): ṽ = 3376, 3001, 2926, 1635, 1430, 1260, 1130, 1105, 1071, 995, 934, 886, 792 cm^{-1}.

HRMS (ESI-TOF) m/z: $[M+H]^+$ calcd. for $C_5H_9O_2^+$: 101.0597; found: 101.0603.

¹H-NMR (400 MHz, $CDCl_3$), after distillation:

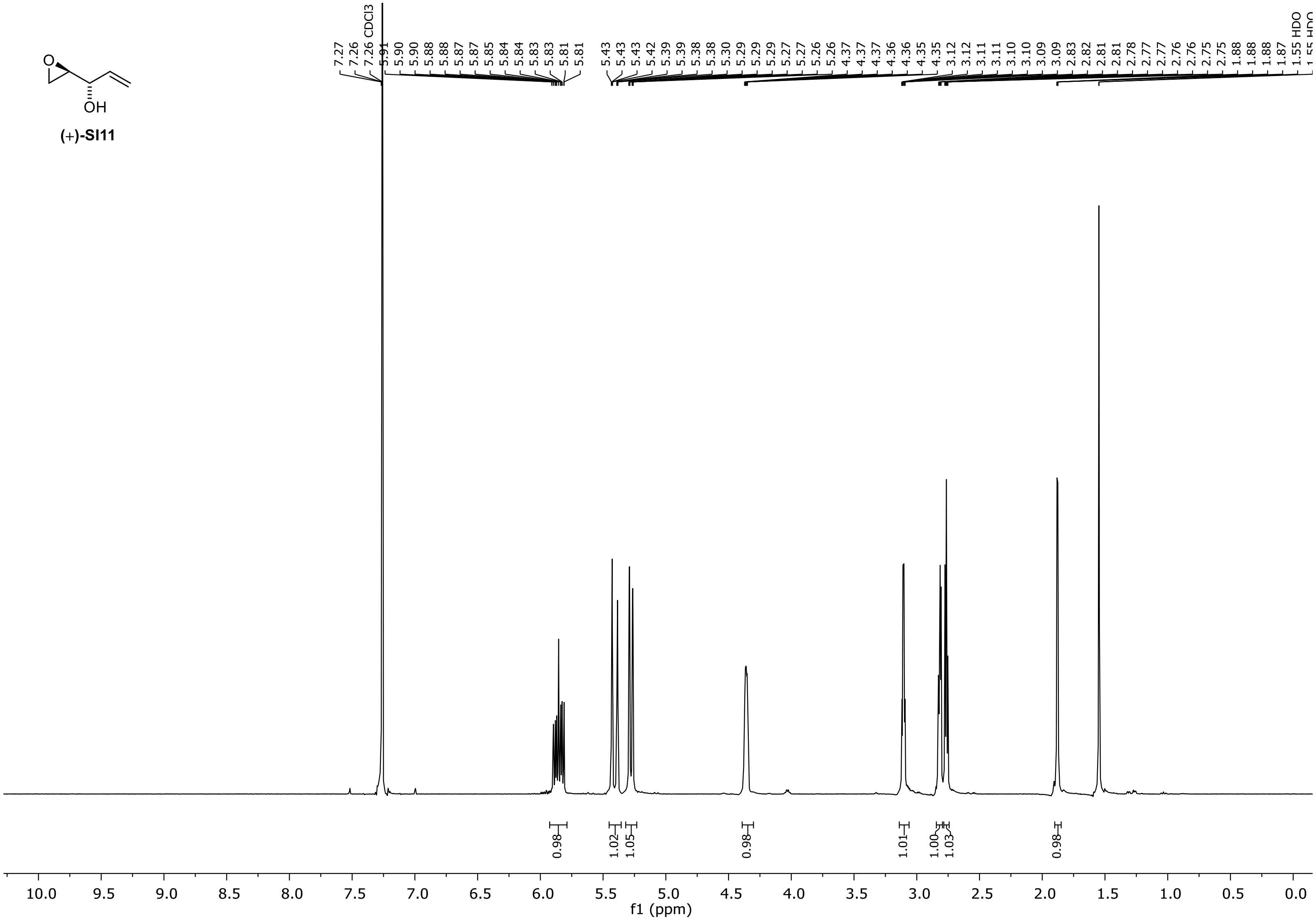

^{13}C-NMR (101 MHz, $CDCl_3$), after distillation:

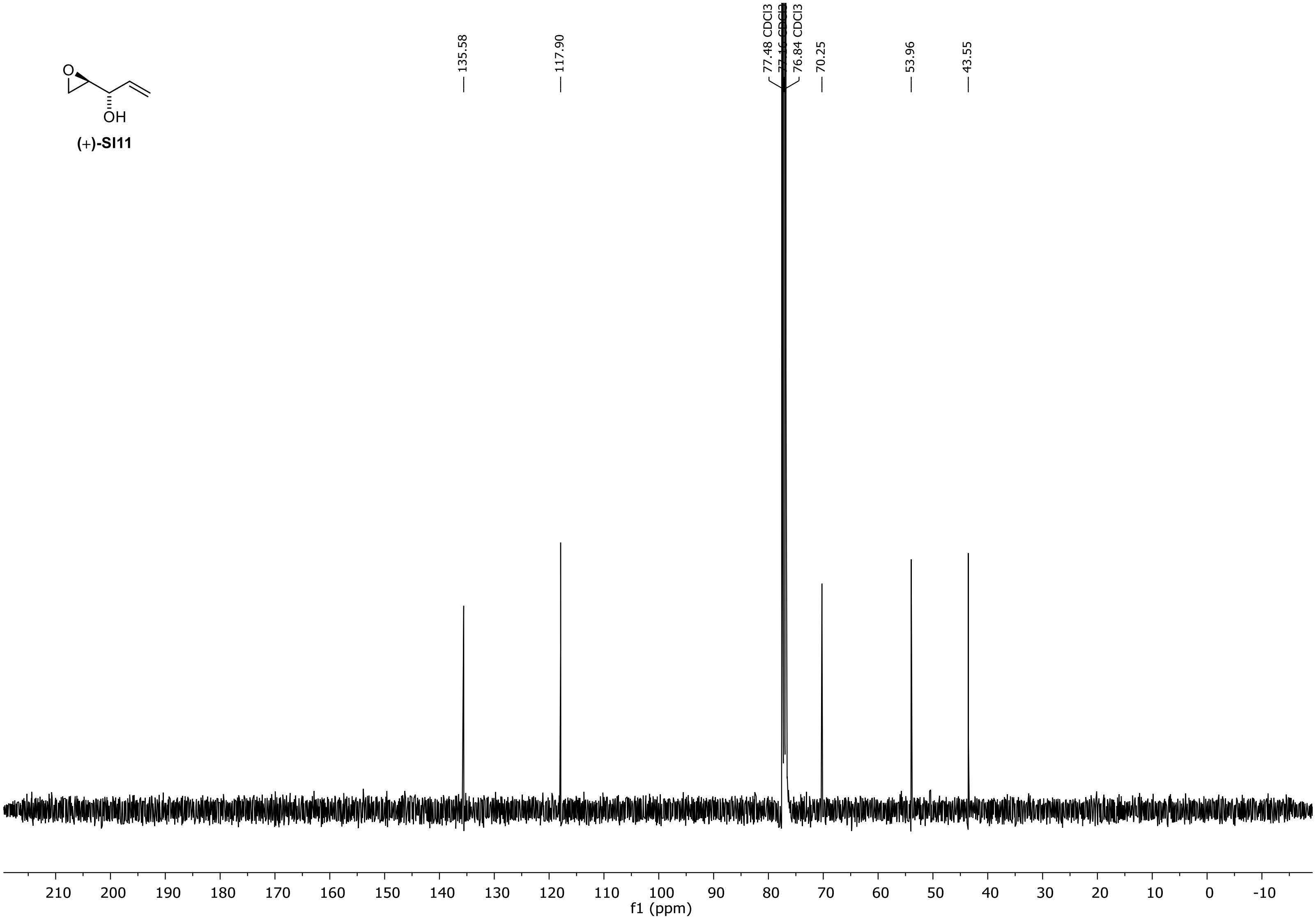

Preparation of *p*-Anisyl Bromide (SI13)[49]

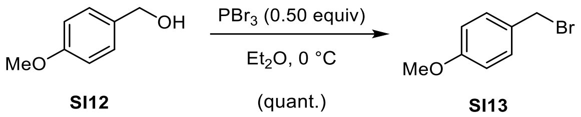

To the colorless solution of *p*-anisyl alcohol (**SI12**) (12.4 g, 90.0 mmol, 1.00 equiv) in anhydrous diethyl ether (100 mL) was added phosphorous tribromide (4.3 mL, 45.3 mmol, 0.50 equiv) at 0 °C. After 2 h of reaction time at this temperature, the mixture was poured into a cooled (0 °C) solution of aqueous saturated sodium bicarbonate (200 mL). Additional diethyl ether (100 mL) was added, the layers were separated, and the organic phase was washed sequentially with aqueous saturated sodium bicarbonate solution (2 x 100 mL) and brine (100 mL). The organic layer was dried over anhydrous magnesium sulfate, filtered, and concentrated under reduced pressure (200 mbar, 40 °C). The *p*-anisyl bromide (18.4 g, 91.5 mmol, quant.) was obtained as a clear oil which was used immediately without further purification for the protection of allylic alcohol **SI11**.

Characterization Data for SI13:

The recorded spectroscopic data matched the reported ones.[49]

^{1}H-NMR (400 MHz, $CDCl_3$): δ = 7.35 – 7.30 (m, 2H), 6.90 – 6.83 (m, 2H), 4.51 (s, 2H), 3.81 (s, 3H) ppm.

^{13}C-NMR (101 MHz, $CDCl_3$): δ = 159.8, 130.6, 130.1, 114.4, 66.0, 55.5, 34.1 ppm.

^{1}H-NMR (400 MHz, $CDCl_3$):

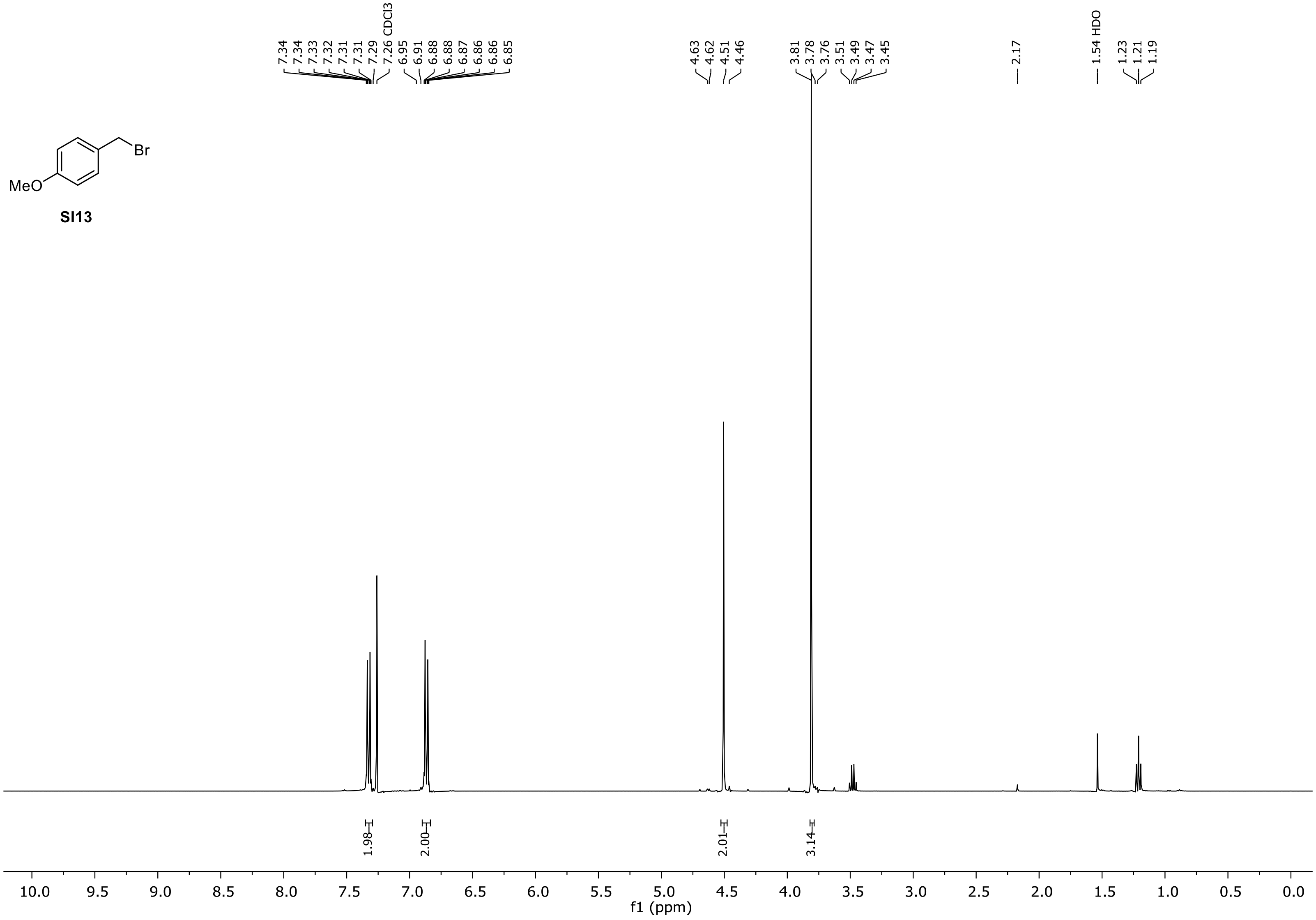

SI13

¹³C-NMR (101 MHz, $CDCl_3$):

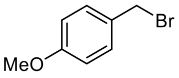

SI13

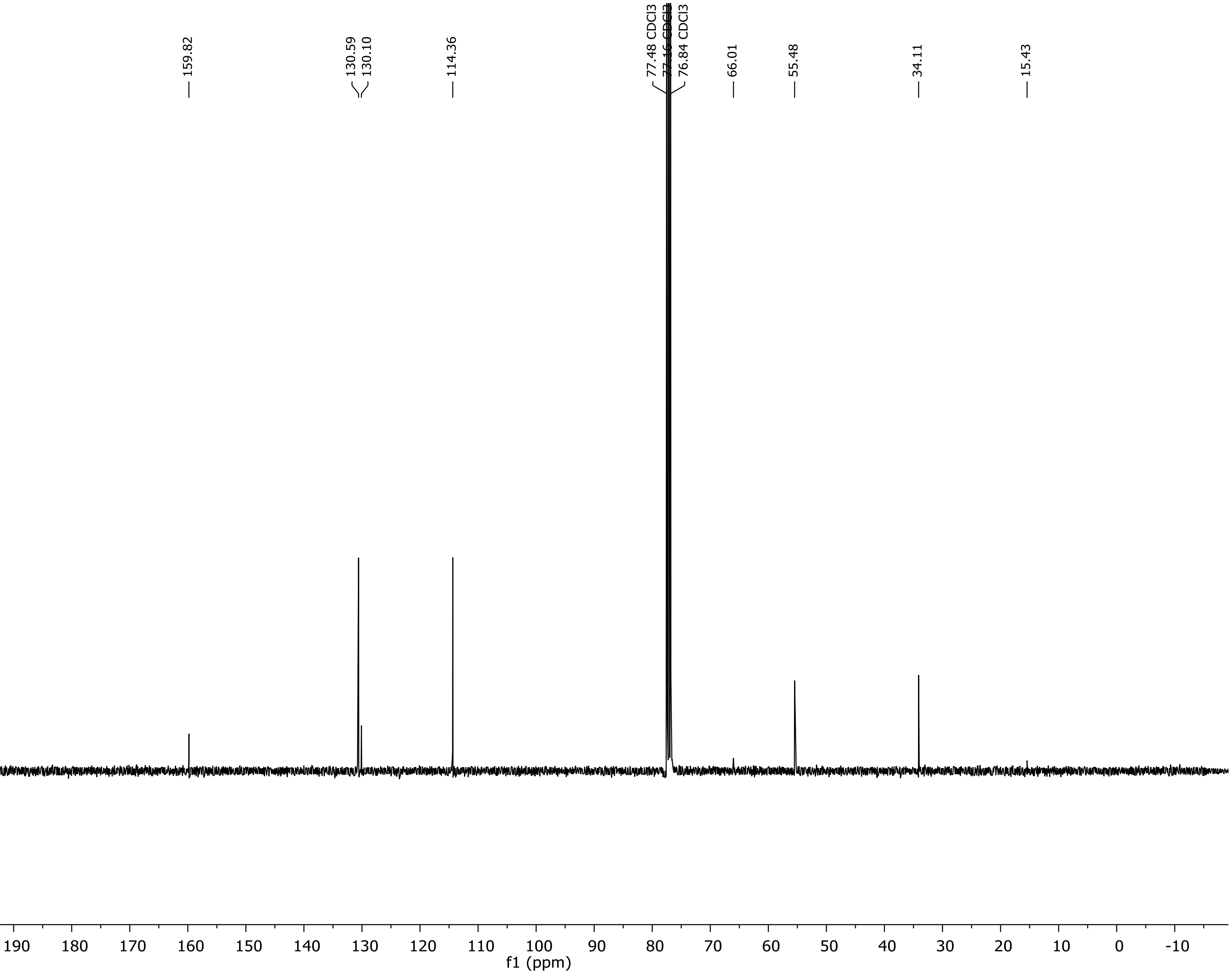

Preparation of 24: PMB-protection of allylic alcohol (+)-SI11[49]

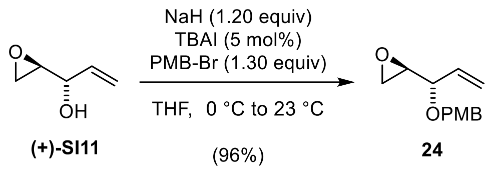

To a cooled (0 °C) suspension of sodium hydride (60% in mineral oil, 3.39 g, 84.9 mmol, 1.20 equiv) and tetrabutylammonium iodide (1.31 g, 3.54 mmol, 5 mol%) in tetrahydrofurane (120 mL) was added a solution of freshly prepared *p*-anisyl bromide (18.4 g, 91.5 mmol, 1.30 equiv) and allylic alcohol **SI11** (7.08 g, 70.7 mmol, 1.00 eq) in tetrahydrofurane (30 mL) over the course of 1 h. After complete addition, the reaction mixture was allowed to warm to ambient temperature and stirring was continued for another 12 h at 23 °C before TLC analysis (20% ethyl acetate in hexanes, CAM) indicated complete consumption of the allylic alcohol **SI11**. The reaction mixture was partitioned between water (100 mL) and diethyl ether (100 mL), the phases were separated, and the aqueous layer was extracted with diethyl ether (2 x 100 mL). The combined organic phases were washed with aqueous saturated sodium chloride solution (100 mL), dried over anhydrous sodium sulfate, filtered, and concentrated under reduced pressure (200 mbar, 40 °C). Flash column chromatography (5 to 20% diethyl ether in hexanes) over silica gel afforded the desired protected allylic ether **24** (14.9 g, 67.6 mmol, 96%) as a colorless volatile liquid.

Characterization Data for 24:

The recorded spectroscopic data matched the reported ones.[49]

^{1}H-NMR (400 MHz, $CDCl_3$): δ =7.26 (d, *J* = 8.5 Hz, 2H), 6.88 (dd, *J* = 8.8, 3.2 Hz, 2H), 5.87 – 5.76 (m, 1H), 5.38 – 5.29 (m, 2H), 4.57 (d, *J* = 11.5 Hz, 1H), 4.40 (d, *J* = 11.5 Hz, 1H), 3.81 – 3.76 (m, 4H), 3.09 – 3.02 (m, 1H), 2.77 (dd, *J* = 5.2, 4.0 Hz, 1H), 2.66 (dd, *J* = 5.3, 2.6 Hz, 1H) ppm.

^{13}C-NMR (101 MHz, $CDCl_3$): δ = 159.4, 134.7, 130.3, 129.5, 119.6, 113.9, 79.2, 70.5, 55.4, 53.4, 45.0 ppm.

¹H-NMR (400 MHz, $CDCl_3$):

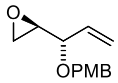

24

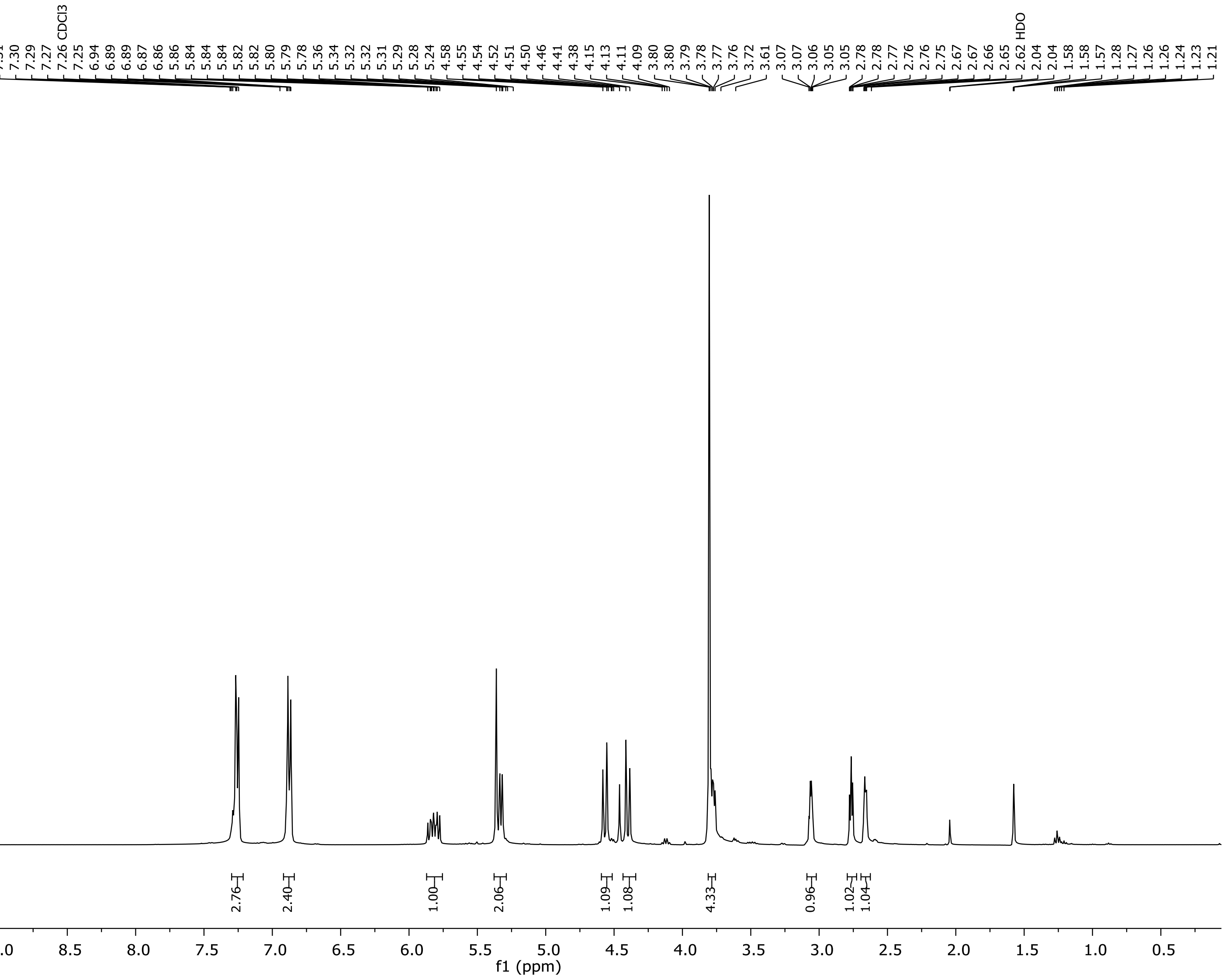

^{13}**C-NMR** (101 MHz, $CDCl_3$):

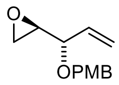

24

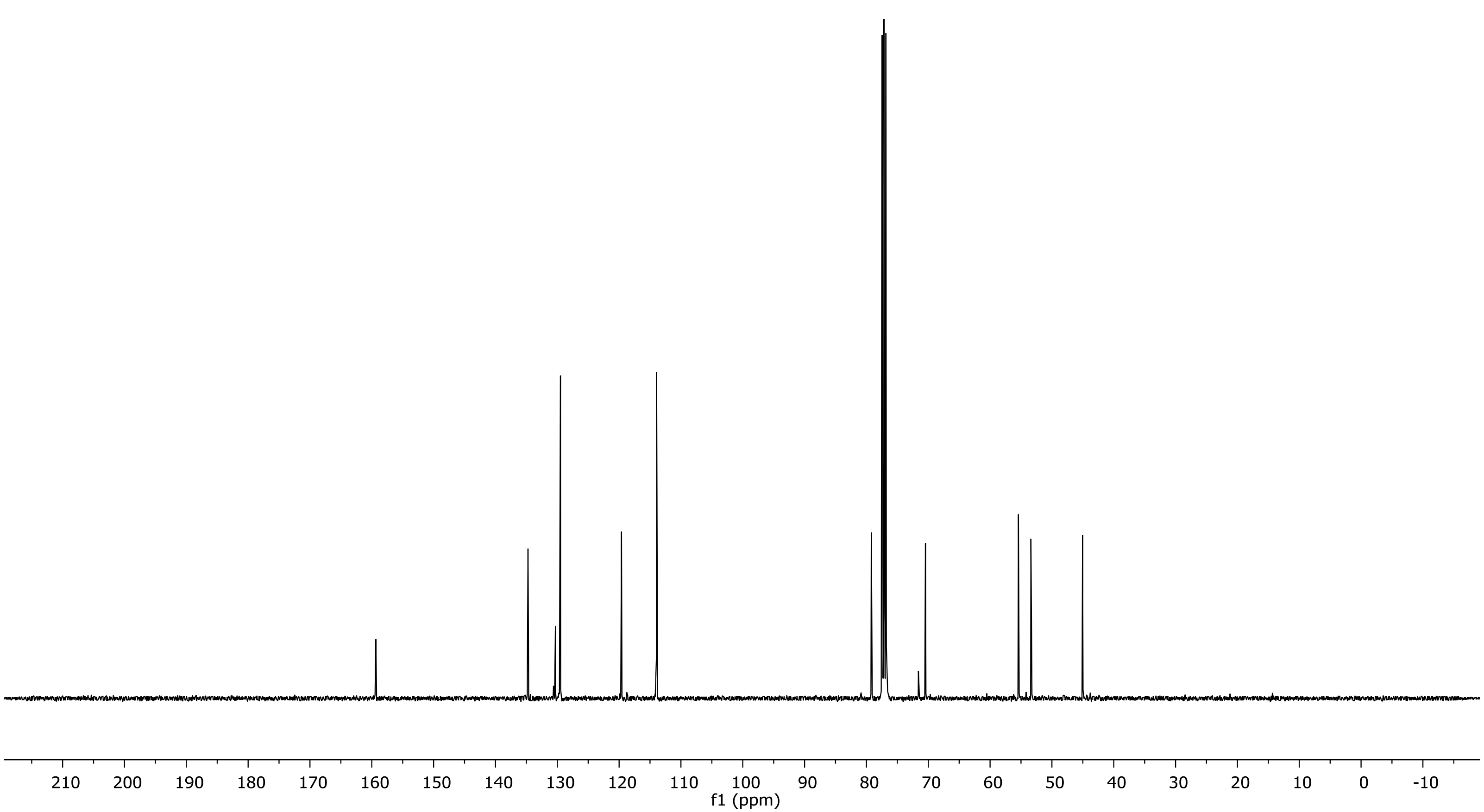

Preparation of 25: Opening of Epoxide 24 with Potassium Cyanide

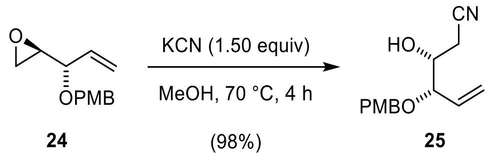

24 25

To the colorless solution of epoxide **24** (13.3 g, 60.4 mmol, 1.00 equiv) in anhydrous methanol (200 mL) was added potassium cyanide (5.90 g, 90.6 mmol, 1.50 equiv) under a nitrogen atmosphere. The mixture was heated at reflux (heating mantle, 70 °C) for 4 h resulting in the reaction mixture turning deep yellow. TLC analysis indicated the absence of epoxide **24** and the reaction mixture was partitioned between water (250 mL). and diethyl ether (250 mL). The phases were separated, and the aqueous layer was extracted with diethyl ether (2 x 200 mL). The combined organic phases were washed with aqueous saturated sodium chloride solution (200 mL), dried over anhydrous magnesium sulfate, and filtered. Concentration of the filtrate afforded the crude product which was purified over silica gel using flash column chromatography (4 to 20% diethyl ether in hexanes) yielding the desired cyanohydrin **25** (14.6 g, 58.9 mmol, 98%) as a colorless volatile liquid.

Characterization Data for 25:

R_f (10% EtOAc in hexanes) = 0.32 (UV, CAM, anisaldehyde).

$[\alpha]_D^{26}$ = +34.2° (c = 2, $CHCl_3$).

^{1}H-NMR (400 MHz, $CDCl_3$): δ = 7.25 – 7.21 (m, 2H), 6.91 – 6.87 (m, 2H), 5.79 (ddd, *J* = 17.3, 10.4, 7.8 Hz, 1H), 5.54 – 5.42 (m, 2H), 4.58 (d, *J* = 11.2 Hz, 1H), 4.32 (d, *J* = 11.2 Hz, 1H), 3.96 – 3.88 (m, 1H), 3.86 – 3.82 (m, 1H), 3.81 (s, 3H), 2.59 (d, *J* = 6.0 Hz, 2H), 2.30 (t, *J* = 5.2 Hz, 1H) ppm.

^{13}C-NMR (101 MHz, $CDCl_3$): δ = 159.6, 134.0, 129.8, 129.6, 122.2, 117.6, 114.1, 81.8, 70.7, 69.4, 55.5, 21.7 ppm.

IR (Diamond-ATR, neat): ṽ = 3465 (br, w), 2868 (w), 1612 (m), 1586 (w), 1513 (s), 1465 (w), 1421 (w), 1302 (m), 1246 (s), 1174 (m), 1058 (s), 1033 (s), 997 (m), 935 (m), 820 (m), 759 (w), 668 (w) cm^{-1}.

HRMS (APCI-TOF) m/z: $[M+H]^+$ calcd. for $C_{14}H_{18}NO_3^+$: 248.1280; found: 248.1281.

¹H-NMR (400 MHz, $CDCl_3$):

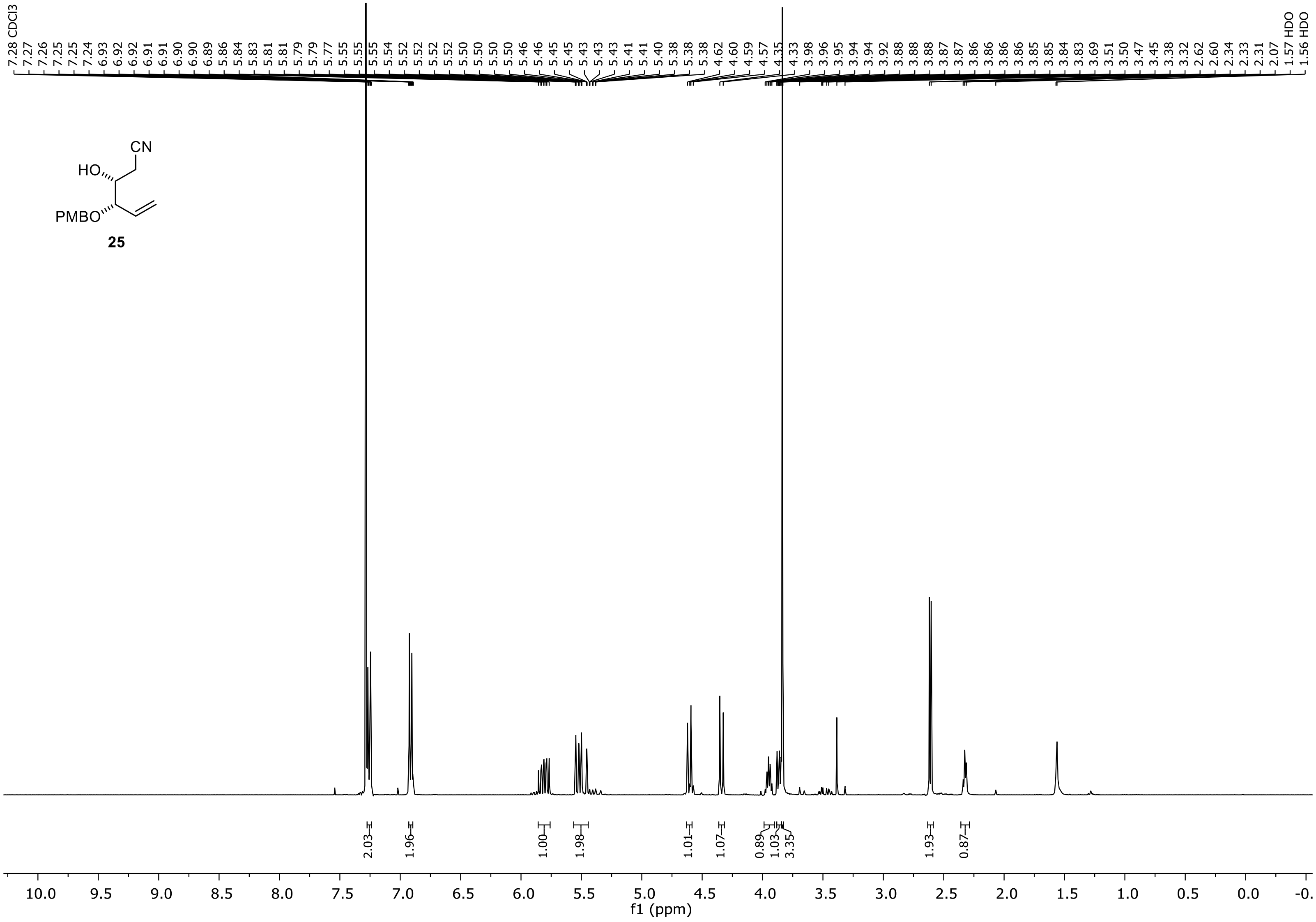

^{13}C-NMR (101 MHz, $CDCl_3$):

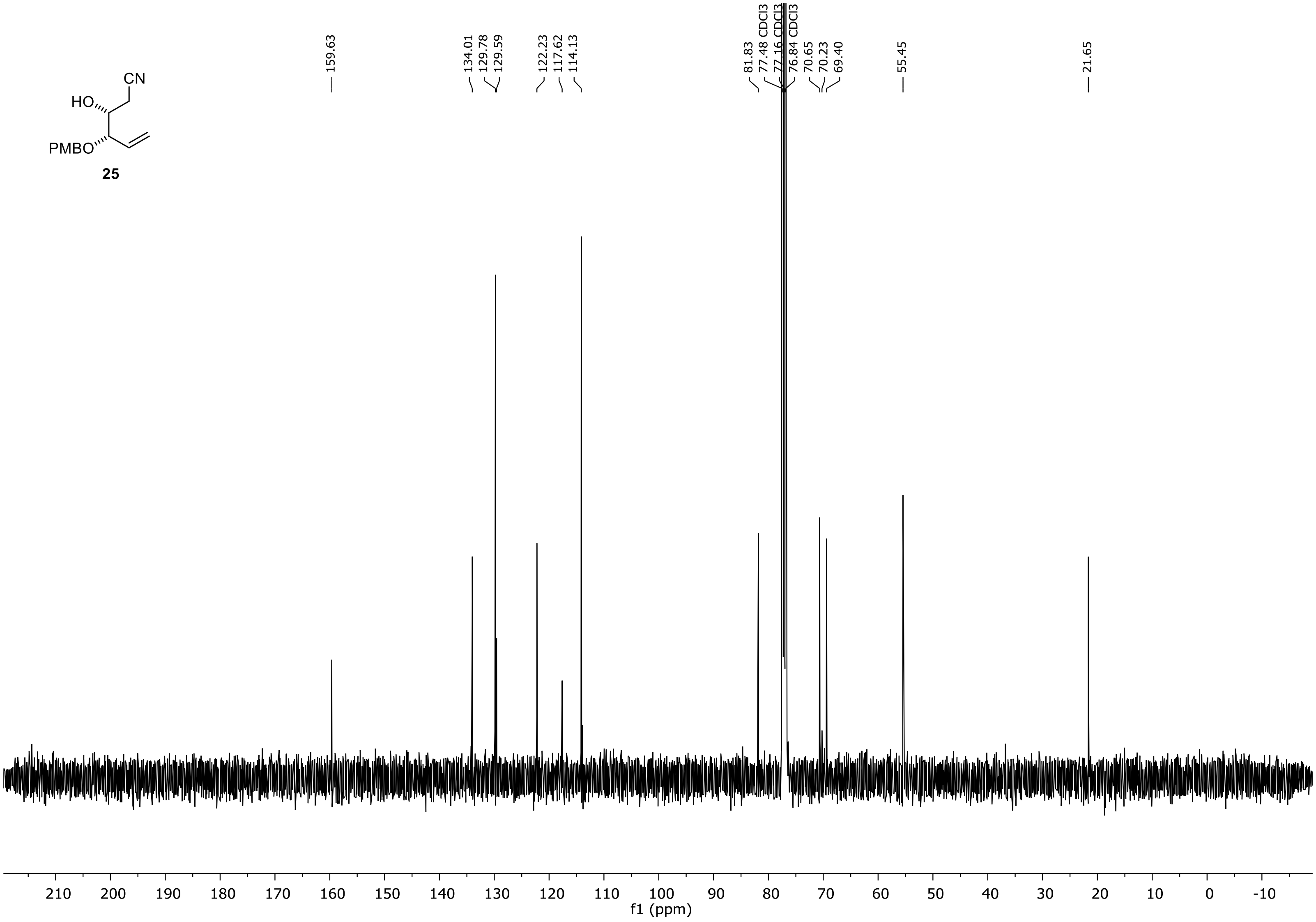

Preparation of 26: TBS-Protection of Homoallylic Alcohol 25

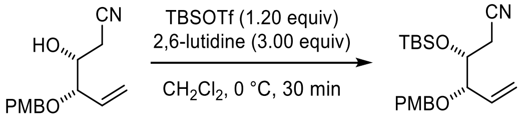

25 **26**

To a solution of homoallylic alcohol **25** (12.2 g, 49.3 mmol, 1.00 equiv) in dichloromethane (170 mL) was added 2,6-lutidine (17.1 mL, 148 mmol, 3.00 equiv) and *tert*-butyldimethylsilyl triflate (13.6 mL, 59.1 mmol, 1.20 equiv) at 0 °C (NOTE 1). The resulting slightly cloudy mixture was stirred for 30 min until TLC analysis (5% ethyl acetate in hexanes, UV, CAM) indicated complete consumption of alcohol **1.103**. Aqueous saturated sodium bicarbonate solution (150 mL) was added, and the aqueous phase was extracted with diethyl ether (3 x 150 mL). The combined organic phases were washed with aqueous saturated sodium chloride solution (200 mL), dried over sodium sulfate, filtered, and concentrated (150 mbar, 40 °C water bath) affording the crude product. Silica gel flash column chromatography (0 to 10% diethyl ether in hexanes) yielded the desired silyl ether **26** (17.9 g, 49.5 mmol, quant.) as a colorless volatile liquid.

NOTE 1: Protection could also be achieved using TBS-Cl (1.20 equiv) and imidazole (3.00 equiv) in CH_2Cl_2, however, the yields were significantly lower (78–86%) when these alternative conditions were used.

.

Characterization Data for 26:

R_f (30% EtOAc in hexanes) = 0.56 (UV, CAM).

$[\alpha]_D^{25}$ = +11.3° (c = 2, $CHCl_3$).

^{1}H-NMR (400 MHz, $CDCl_3$): δ = 7.25 – 7.22 (m, 2H), 6.90 – 6.85 (m, 2H), 5.74 (ddd, *J* = 17.1, 10.5, 7.4 Hz, 1H), 5.42 – 5.33 (m, 2H), 4.54 (d, *J* = 11.1 Hz, 1H), 4.33 (d, *J* = 11.1 Hz, 1H), 3.88 (ddd, *J* = 6.1, 5.5, 4.4 Hz, 1H), 3.81 (s, 3H), 3.78 (dd, *J* = 7.1, 6.0 Hz, 1H), 2.70 (dd, *J* = 16.7, 5.5 Hz, 1H), 2.49 (dd, *J* = 16.7, 4.4 Hz, 1H), 0.89 (s, 9H), 0.11 (s, 3H), 0.05 (s, 3H) ppm.

^{13}C-NMR (101 MHz, $CDCl_3$): δ = 159.4, 135.3, 130.1, 129.7, 120.6, 118.0, 114.0, 82.4, 70.9, 70.8, 55.4, 25.9, 23.3, 18.1, -4.2, -4.6 ppm.

IR (Diamond-ATR, neat): ṽ = 2954 (w), 2929 (w), 2886 (w), 2856 (w), 1613 (w), 1514 (m), 1464 (w), 1420 (w), 1389 (w), 1362 (w), 1302 (w), 1247 (s), 1173 (w), 1109 (m), 1034 (m), 1005 (w), 923 (m), 825 (s), 809 (s), 777 (s), 683 (w), 668 (w) cm^{-1}.

HRMS (APCI-TOF) m/z: $[M+H]^+$ calcd. for $C_{20}H_{32}NO_3Si^+$: 362.2146; found 362.2144.

^{1}H-NMR (400 MHz, $CDCl_3$):

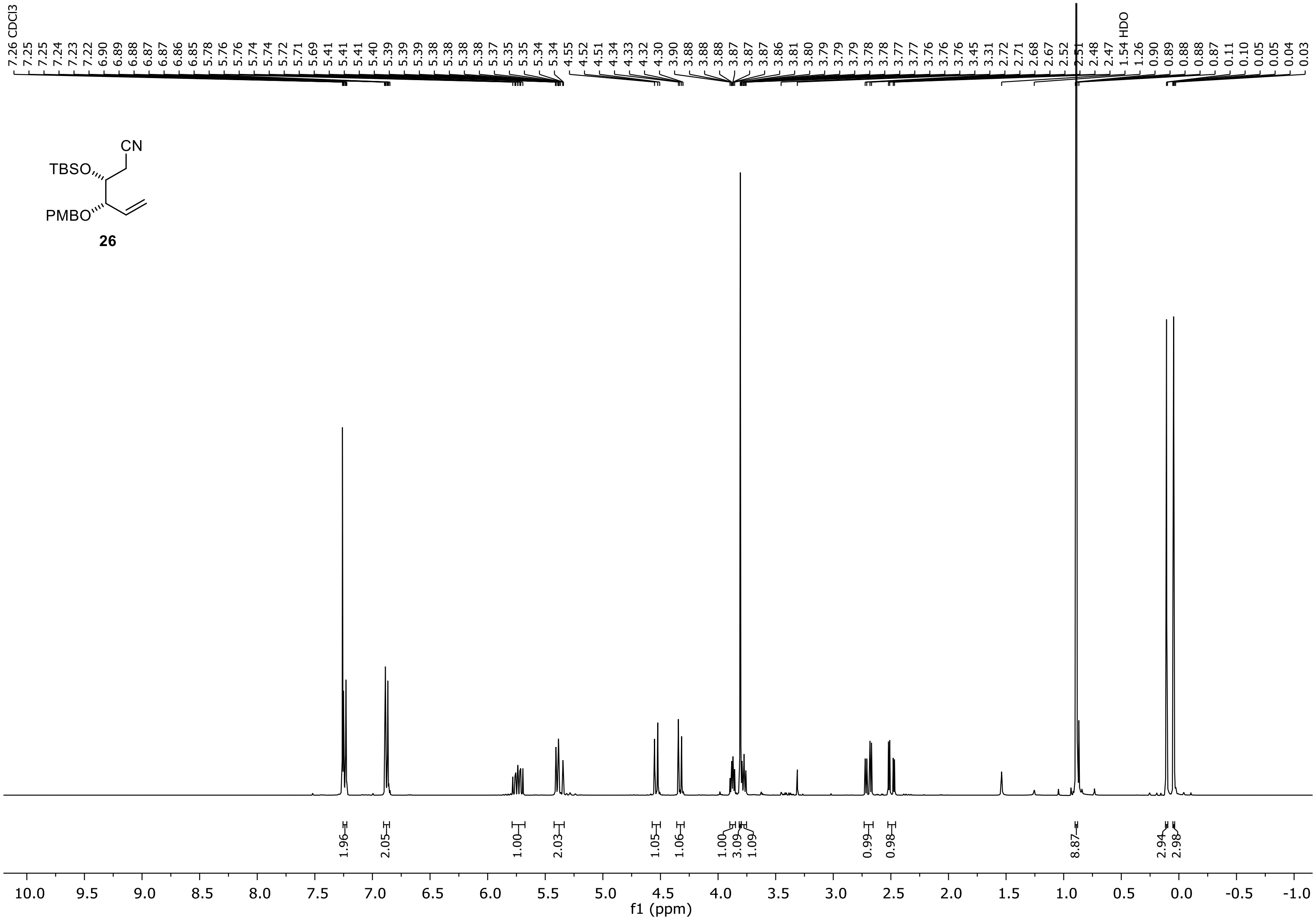

^{13}C-NMR (101 MHz, $CDCl_3$):

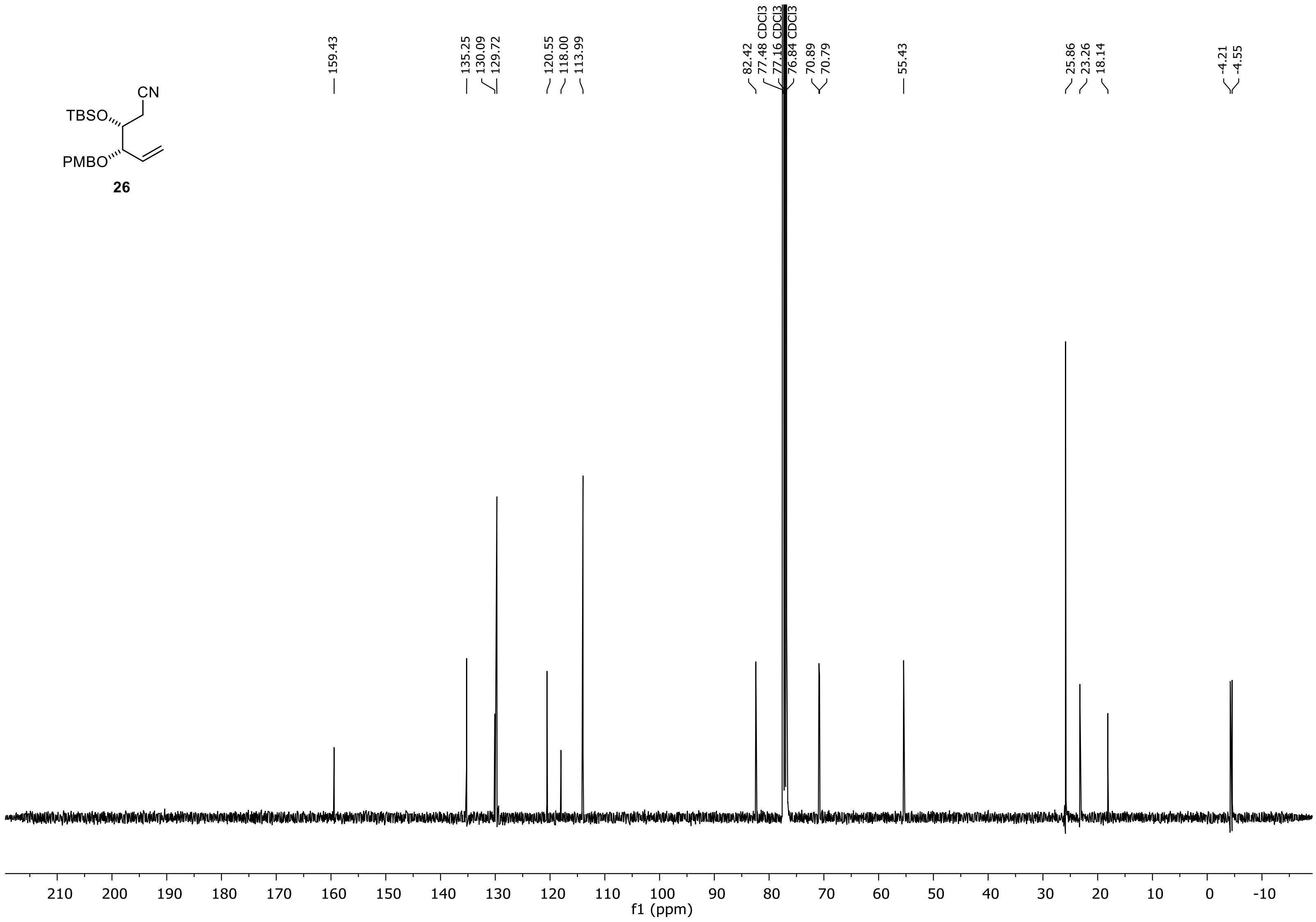

Preparation of 21: DIBAL-H reduction of nitrile 26

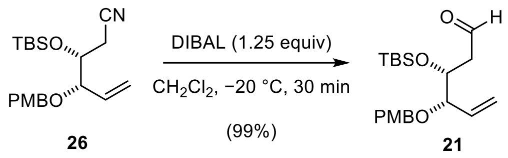

To a solution of nitrile **26** (6.00 g, 16.6 mmol, 1.00 equiv) in dry dichloromethane (60 mL) was added DIBAL solution (1 M in toluene, 22 mL, 22.0 mmol, 1.24 equiv) at −20 °C. After 30 min TLC-analysis (10% ethyl acetate in hexane, UV, *p*-anisaldehyde) (NOTE 1) indicated complete consumption of the starting material and excess DIBAL was quenched by careful addition of half-saturated aqueous Rochelle's salt solution (50 mL, NOTE2). The mixture was stirred vigorously for 2 hours while being warmed to ambient temperature. The phases were separated, and the aqueous phase was extracted further with diethyl ether (3 x 50mL). The combined organic extracts were washed with aqueous saturated sodium chloride solution (100 mL), dried over magnesium sulfate, filtered, and concentrated (150 mbar, 40 °C water bath). The crude product was purified over silica gel by MPLC Combi-flash column chromatography (80 g silica gel, 0 to 15% diethyl ether in hexanes over 35 min) to yield the desired volatile aldehyde **21** (5.98 g, 16.4 mmol, 99%) as a colorless liquid (NOTE 3).

NOTE 1: The starting material and product are copolar in a variety of solvent mixtures. The best way to monitor the reaction is by staining the developed TLC plate with different stains (*p*-anisaldehyde, DNP and vanillin) and carefully analyze the differently staining compounds. It is important to ensure completion of the reaction as residual starting material cannot be separated from the product and will decrease the yields of the following Evans aldol reaction significantly.

NOTE 2: The first 2 mL of the aqueous Rochelle's salt solution were added dropwise while continuously observing the stirring mixture to prevent an excessive quench from occurring.

NOTE 3: Aldehyde **21** was always prepared freshly as needed to avoid long term storage and potential decomposition of the building block.

Characterization Data for 21:

R$_f$ (30% EtOAc in hexanes) = 0.53 (UV, anisaldehyde, vanillin, DNP).

$[\boldsymbol{\alpha}]_{\mathbf{D}}^{\mathbf{22}}$ = +24.8° (c = 2, $CHCl_3$).

^{1}H-NMR (400 MHz, $CDCl_3$): δ = 9.76 (t, *J* = 2.5 Hz, 1H), 7.25 – 7.20 (m, 2H), 6.90 – 6.83 (m, 2H), 5.76 (ddd, *J* = 17.2, 10.4, 7.5 Hz, 1H), 5.38 – 5.26 (m, 2H), 4.52 (d, *J* = 11.4 Hz, 1H), 4.31 (d, *J* = 11.3 Hz, 1H), 4.19 (q, *J* = 5.5 Hz, 1H), 3.80 (s, 3H), 3.71 (ddt, *J* = 7.3, 5.3, 1.0 Hz, 1H), 2.63 (ddd, *J* = 15.9, 5.7, 2.5 Hz, 1H), 2.51 (ddd, *J* = 15.9, 5.7, 2.5 Hz, 1H), 0.85 (s, 9H), 0.05 (s, 3H), 0.04 (s, 3H) ppm.

^{13}C-NMR (101 MHz, $CDCl_3$): δ = 201.5, 159.3, 135.7, 130.3, 129.7, 119.8, 113.9, 83.5, 71.0, 70.4, 55.4, 48.3, 25.9, 18.2, −4.2, −4.6 ppm.

IR (Diamond-ATR, neat): ṽ = 2955 (m), 2929 (m), 2857 (m), 1725 (m), 1613 (w), 1514 (m), 1464 (w), 1361 (w), 1302 (w), 1249 (s), 1173 (w), 1103 (m), 1034 (w), 932 (w), 836 (s), 778 (m), 668 (s) cm^{-1}.

HRMS (APCI-TOF) m/z: $[M+H-H_2O]^+$ calcd. for $C_{20}H_{31}O_3Si^+$: 347.2037; found: 347.2024.

^{1}H-NMR (400 MHz, $CDCl_3$):

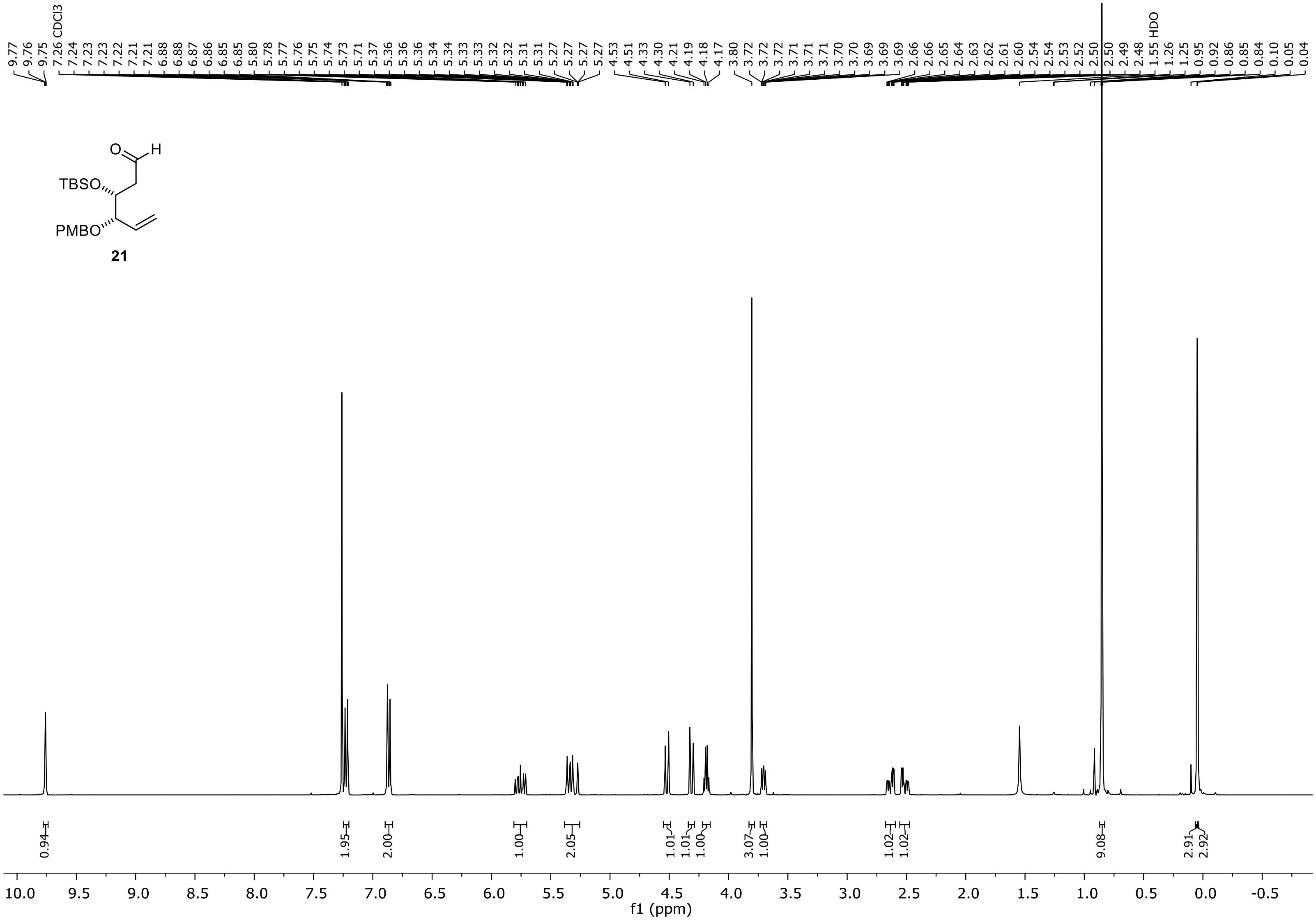

^{13}C-NMR (101 MHz, $CDCl_3$):

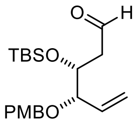

21

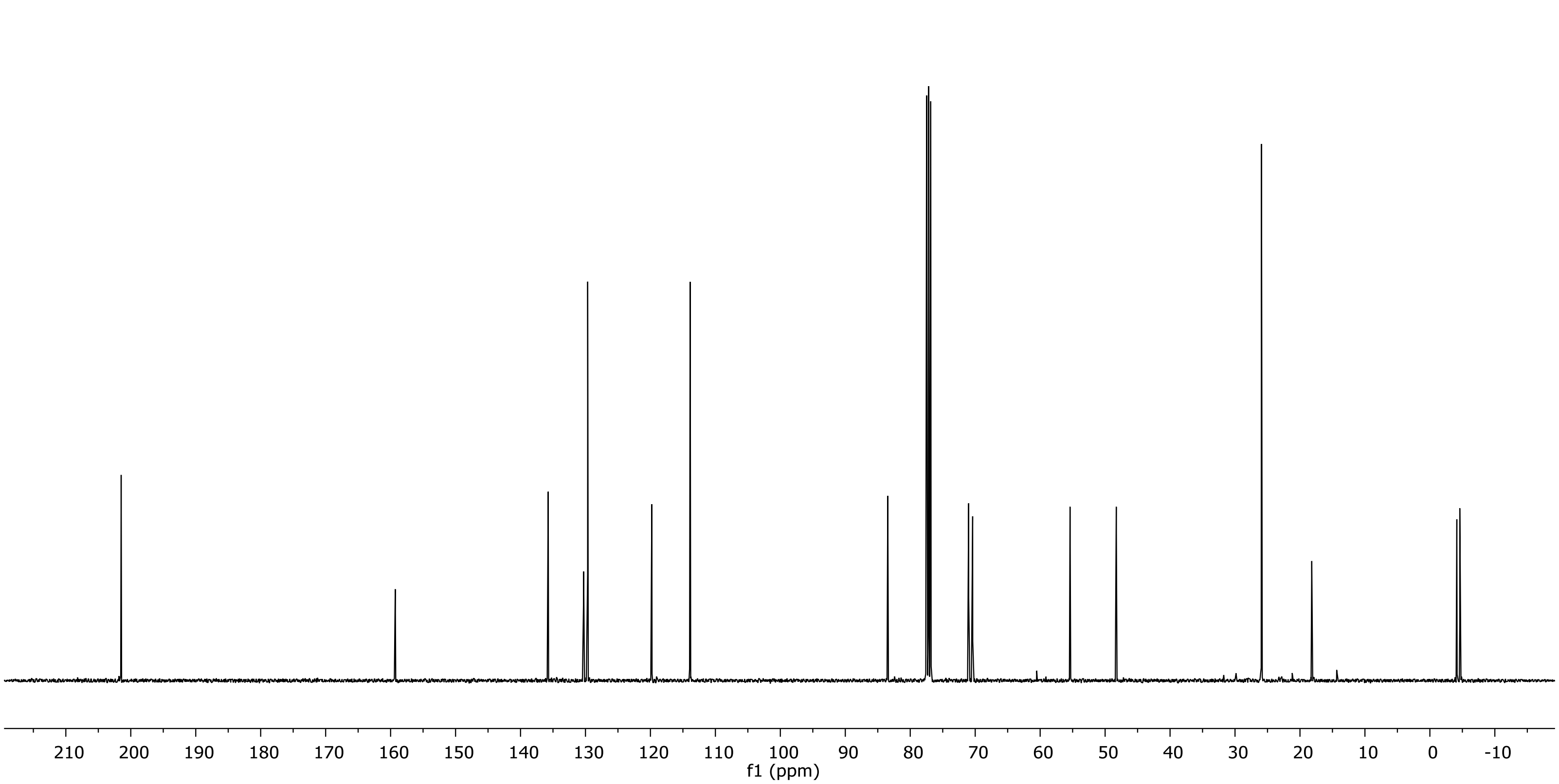

Preparation of 28: Evans Aldol Addition of Propionyl Oxazolidinone 16 to Aldehyde 21

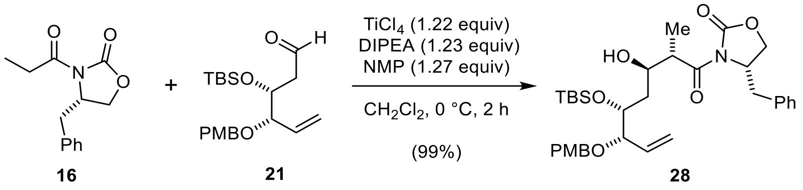

To the colorless solution of propionyl oxazolidinone **16** (4.59 g, 19.7 mmol, 1.20 equiv) in dichloromethane (80 mL) was added a freshly prepared titanium tetrachloride (2.2 mL, 20.0 mmol, 1.22 equiv) solution in dichloromethane (20 mL) dropwise at 0 °C. Initially the solution turned yellow, upon complete addition a yellow precipitate formed. After 15 min, anhydrous DIPEA (3.5 mL, 20.1 mmol, 1.23 equiv) was added dropwise, resulting in the formation of white fumes within the reaction vessel and a change of color of the solution to a deep red. After another 20 min of stirring at 0 °C, NMP (2.0 mL, 20.8 mmol, 1.27 equiv) (NOTE 1) was added to the reaction mixture. After 15 min of stirring, a solution of aldehyde **21** (5.97 g, 16.4 mmol, 1.00 equiv) in dichloromethane (20 mL) was added dropwise over 10 min. The flask containing the starting material, and the syringe were washed with additional dichloromethane (5 mL). Stirring was continued at 0 °C for 2 h when TLC-analysis (20% ethyl acetate in hexanes, UV, CAM, anisaldehyde) indicated complete consumption of aldehyde **21** and the spot-to-spot conversion to aldol product **28**. Excess titanium enolate was quenched by the addition of aqueous saturated ammonium chloride solution (200 mL). The layers were separated, and the aqueous phase was extracted with ethyl acetate (3 x 100 mL). The combined organic layers were dried over magnesium sulfate, filtered, and concentrated under reduced pressure. Silica gel flash column chromatography (2 to 20% EtOAc in hexanes) afforded the *syn*-aldol product **28** (9.71 g, 16.2 mmol, 99%) as a colorless viscous oil.

NOTE 1: The addition of NMP was crucial for the success of the reaction and improved the yields of this reactions from moderate (52–64%) to excellent (96–99%).[34]

Characterization Data for 28:

R_f (20% ethyl acetate in hexanes) = 0.47 (UV, CAM, anisaldehyde).

$[\alpha]_D^{22}$ = +48.0° (c = 2, $CHCl_3$).

^{1}H-NMR (400 MHz, $CDCl_3$): δ = 7.36 – 7.29 (m, 3H), 7.29 – 7.25 (m, 2H)*, 7.22 – 7.19 (m, 2H), 6.87 – 6.83 (m, 2H), 5.79 (ddd, *J* = 17.3, 10.4, 7.8 Hz, 1H), 5.33 (dd, *J* = 10.5, 1.9 Hz, 1H), 5.27 (ddd, *J* = 17.4, 1.8, 0.8 Hz, 1H), 4.67 (dtt, *J* = 9.9, 6.7, 3.5 Hz, 1H), 4.51 (d, *J* = 11.2 Hz, 1H), 4.33 (d, *J* = 11.3 Hz, 1H), 4.24 – 4.19 (m, 1H), 4.19 – 4.14 (m, 2H), 4.01 (dt, *J* = 6.0, 4.8 Hz, 1H), 3.79 (s, 3H), 3.77 – 3.71 (m, 2H), 3.52 – 3.30 (br, 1H), 3.26 (dd, *J* = 13.4, 3.4 Hz, 1H), 2.77 (ddd, *J* = 13.3, 9.6, 2.9 Hz, 1H), 1.68 – 1.62 (m, 2H), 1.25 (d, *J* = 7.0 Hz, 3H), 0.87 (s, 9H), 0.09 (s, 3H), 0.05 (s, 3H) ppm.

**Over-integrating due to overlap with the NMR solvent.*

^{13}C-NMR (101 MHz, $CDCl_3$): δ = 176.9, 159.2, 153.2, 136.1, 135.4, 130.7, 129.7, 129.6, 129.1, 127.5, 119.5, 113.8, 83.5, 72.7, 70.2, 68.5, 66.2, 55.41, 55.39, 43.2, 38.0, 37.0, 26.1, 18.3, 11.3, −4.1, −4.5 ppm.

IR (Diamond-ATR, neat): $\tilde{\nu}$ = 2953 (w), 2928 (w), 2857 (w), 1781 (s), 1697 (w), 1613 (w), 1514 (m), 1455 (w), 1387 (m), 1301 (w), 1247 (s), 1209 (m), 1060 (s), 1033 (s), 1006 (m), 928 (w), 832 (m), 812 (w), 778 (m), 703 (w), 668 (m) cm^{-1}.

HRMS (APCI-TOF) m/z: $[M+H-H_2O]^+$ calcd. for $C_{33}H_{46}NO_6Si^+$: 580.3089; found: 580.3087.

[1]**H-NMR** (400 MHz, $CDCl_3$):

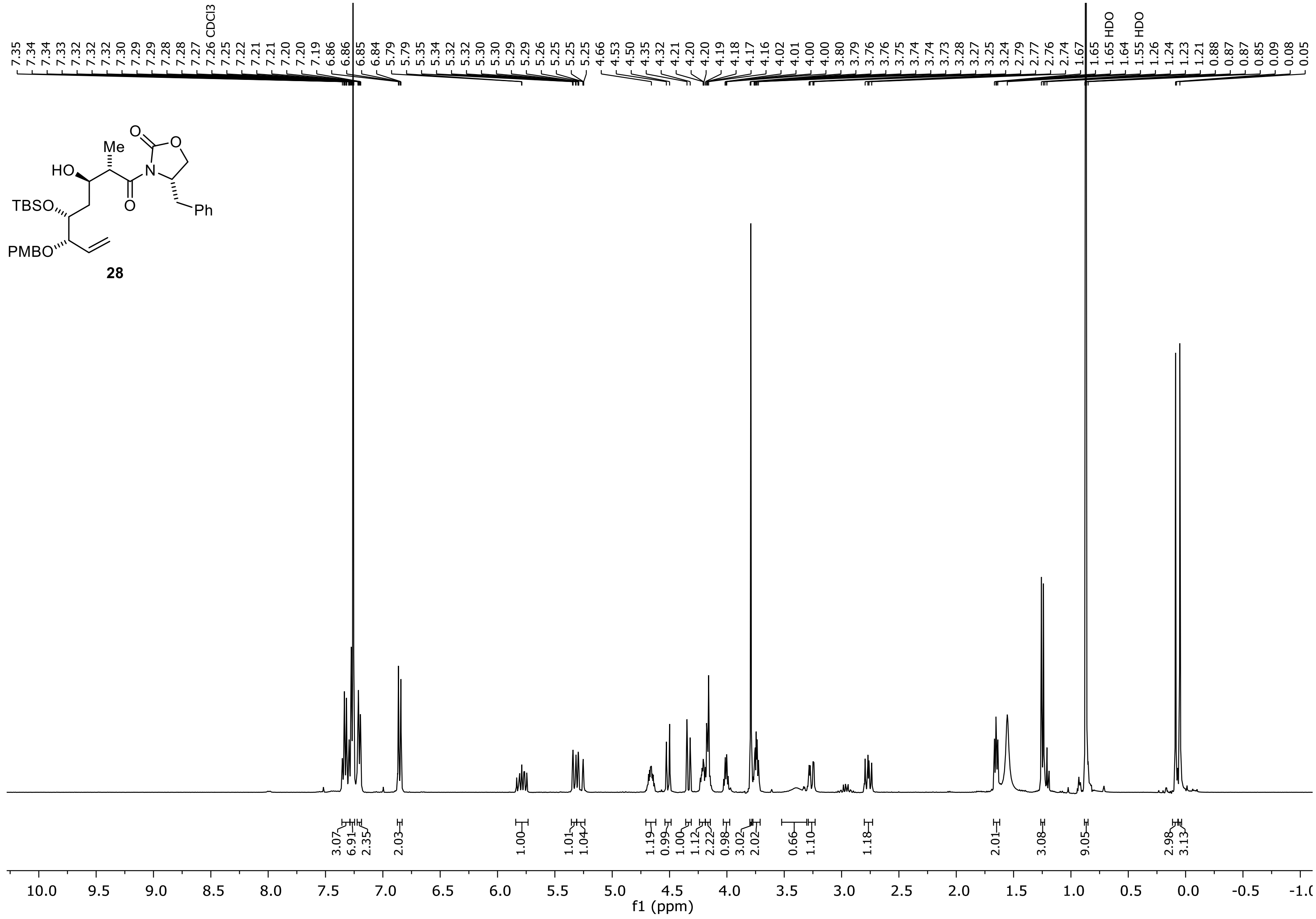

13C-NMR (101 MHz, $CDCl_3$):

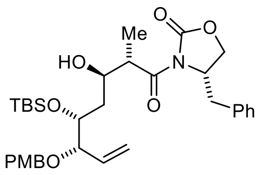

28

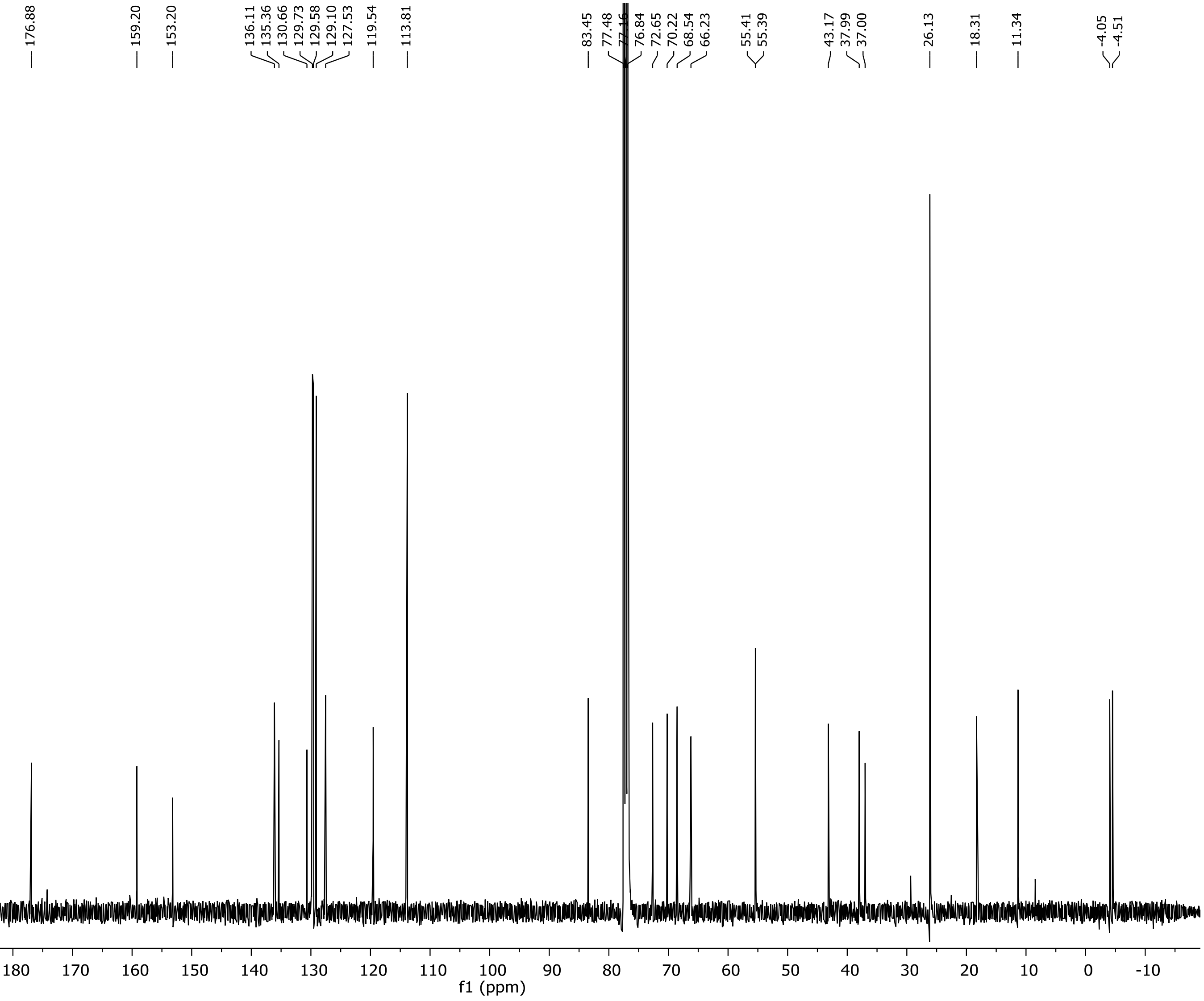

Preparation of 29: Benzoylation of Alcohol 28

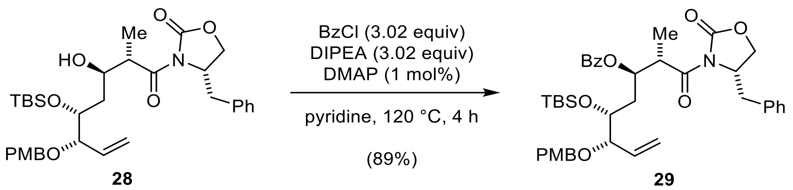

To a solution of aldol product **28** (9.34 g, 14.8 mmol, 1.00 equiv) in anhydrous pyridine (50 mL) was added DMAP (18.2 mg, 0.149 mmol, 0.01 equiv), DIPEA (7.8 mL, 44.8 mmol, 3.02 equiv) and benzoyl chloride (5.2 mL, 44.8 mmol, 3.02 equiv) successively at room temperature. The resulting reaction mixture was heated to 120 °C using a heating mantle and stirring was continued for 4 h until TLC-analysis (10% ethyl acetate in hexanes, UV, CAM) indicated completion of the reaction. After cooling of the brown mixture to room temperature, it was partitioned between aqueous saturated sodium bicarbonate solution (150 mL) and ethyl acetate (150 mL). The phases were separated, and the aqueous layer was extracted with EtOAc (2 x 150 mL). The combined organic fractions were washed with aqueous saturated sodium chloride solution (200 mL), dried over anhydrous magnesium sulfate, filtered, and concentrated affording the crude product as a brown residue. Silica gel flash column chromatography (1 to 16% EtOAc in hexanes) afforded benzoate **29** (9.29 g, 13.2 mmol, 89%) as a pale-yellow viscous oil.

Characterization Data for 29:

R_f (10% ethyl acetate in hexanes) = 0.49 (UV, CAM, anisaldehyde).

^{1}H-NMR (400 MHz, $CDCl_3$): δ = 7.99 – 7.94 (m, 2H), 7.59 – 7.53 (m, 1H), 7.43 (t, *J* = 7.7 Hz, 2H), 7.31 (dd, *J* = 8.0, 6.3 Hz, 2H), 7.27 – 7.21 (m, 3H)*, 7.19 (dd, *J* = 6.8, 1.6 Hz, 2H), 6.85 – 6.81 (m, 2H), 5.82 (ddd, *J* = 17.3, 10.4, 7.7 Hz, 1H), 5.61 (dt, *J* = 10.4, 2.7 Hz, 1H), 5.35 – 5.30 (m, 1H), 5.25 (ddd, *J* = 17.3, 1.9, 0.9 Hz, 1H), 4.58 – 4.51 (m, 1H), 4.49 (d, *J* = 11.5 Hz, 1H), 4.34 (d, *J* = 11.4 Hz, 1H), 4.24 (dd, *J* = 8.8, 7.5 Hz, 1H), 4.16 – 4.07 (m, 2H), 3.79 (s, 4H), 3.72 (dd, *J* = 7.8, 2.7 Hz, 1H), 3.26 (dd, *J* = 13.4, 3.4 Hz, 1H), 2.75 (dd, *J* = 13.4, 9.8 Hz, 1H), 1.97 (ddd, *J* = 14.5, 10.4, 2.7 Hz, 1H), 1.82 (ddd, *J* = 14.5, 8.9, 2.6 Hz, 1H), 1.26 (d, *J* = 6.9 Hz, 3H), 0.87 (s, 9H), −0.04 (s, 3H), −0.07 (s, 3H) ppm.

**Overintegrating due to aromatic signal overlapping with the NMR solvent.*

^{13}C-NMR (101 MHz, $CDCl_3$): δ = 174.1, 166.3, 159.1, 153.7, 135.8, 135.4, 133.1, 130.9, 130.4, 129.7, 129.6, 129.4, 129.0, 128.5, 127.3, 119.4, 113.7, 84.3, 71.9, 71.8, 70.2, 66.5, 56.3, 55.4, 42.1, 38.0, 36.8, 26.2, 18.4, 10.2, −3.6, −5.1 ppm.

IR (Diamond-ATR, neat): ṽ = 2967 (m), 2864 (m), 1781 (m), 1249 (m), 1055 (s), 1033 (m), 1002 (m), 668 (s) cm^{-1}.

HRMS (APCI-TOF) m/z: $[M+NH_4]^+$ calcd. for $C_{40}H_{55}N_2O_8Si^+$: 719.3722; found: 719.3723.

¹H-NMR (400 MHz, $CDCl_3$):

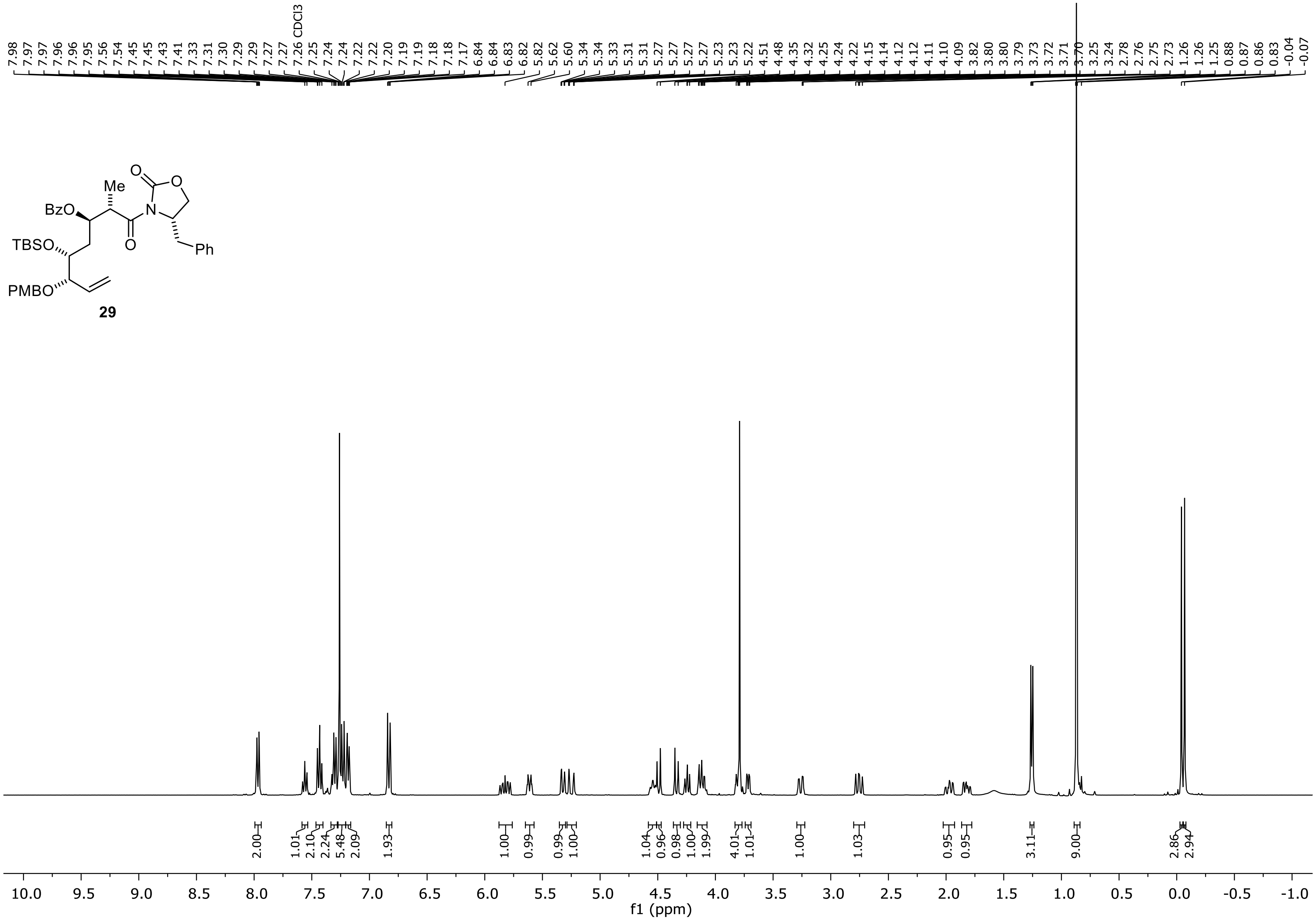

^{13}C-NMR (101 MHz, $CDCl_3$):

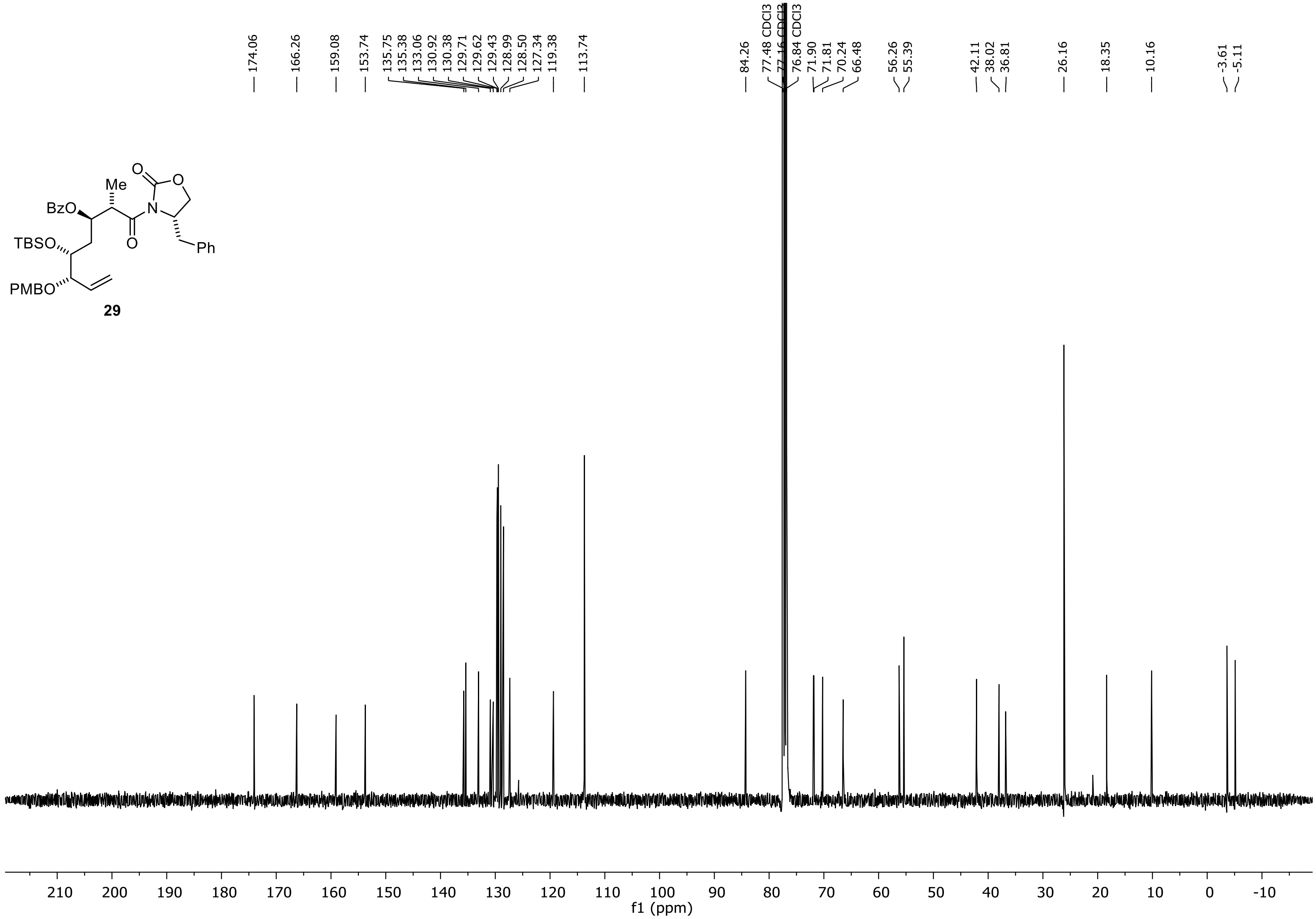

Preparation of 30: Hydrolytic Cleavage of the Oxazolidinone Auxiliary in 29

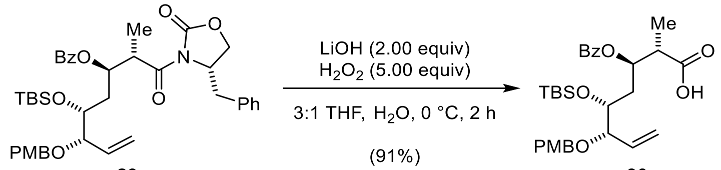

To a solution of *N*-acyl-oxazolidinone **29** (7.40 g, 10.0 mmol, 1.00 equiv) in THF/water (3:1, 120 mL) were added hydrogen peroxide solution (35%, 4.3 mL, 50.2 mmol, 5.01 equiv) and lithium hydroxide (480 mg, 20.0 mmol, 2.00 equiv) sequentially while cooling with an ice/water bath (0 °C). After 2 h of stirring TLC analysis (50% EtOAc in hexanes, UV, CAM) (NOTE 1) indicated complete consumption of starting material. Excess peroxide was quenched by the addition of aqueous saturated sodium thiosulfate solution (1.0 mL). The pH was adjusted to pH = 2 by dropwise addition of aqueous hydrochloric acid solution (1 M) and the aqueous layer was extracted with ethyl acetate (3 x 80 mL). The combined organic phases were dried over anhydrous magnesium sulfate, filtered, and concentrated under reduced pressure affording the crude product which was subjected to silica gel flash column chromatography (50 to 100% ethyl acetate in hexanes), yielding the desired carboxylic acid **30** (4.92 g, 9.07 mmol, 91%) as a clear, viscous oil.

NOTE 1: If TLC analysis did not indicate complete consumption of the oxazolidinone starting material after 2 h, the reaction would still need be quenched as longer reaction times would lead to significant decomposition of the material.

Characterization Data for 30:

R*f* (50% EtOAc in hexanes) = 0.67 (UV, CAM).

^{1}H-NMR (400 MHz, $CDCl_3$): δ = 7.99 (dt, *J* = 8.1, 1.1 Hz, 2H), 7.55 (td, *J* = 7.1, 6.5, 1.2 Hz, 1H), 7.42 (t, *J* = 7.7 Hz, 2H), 7.24–7.21 (m, 2H), 6.86–6.81 (m, 2H), 5.81 (ddd, *J* = 17.6, 10.4, 7.8 Hz, 1H), 5.48 (dt, *J* = 9.6, 3.6 Hz, 1H), 5.32 (dd, *J* = 10.4, 1.8 Hz, 1H), 5.28–5.21 (m, 1H), 4.50 (d, *J* = 11.5 Hz, 1H), 4.32 (d, *J* = 11.4 Hz, 1H), 3.89 (dt, *J* = 8.9, 2.9 Hz, 1H), 3.79 (d, *J* = 0.8 Hz, 3H), 3.70 (dd, *J* = 7.8, 2.8 Hz, 1H), 2.95 (qd, *J* = 7.1, 4.3 Hz, 1H), 1.93 (ddd, *J* = 14.8, 9.4, 2.8 Hz, 1H), 1.83 (ddd, *J* = 14.7, 8.8, 3.0 Hz, 1H), 1.23 (d, *J* = 7.1 Hz, 3H), 0.87 (d, *J* = 1.9 Hz, 9H), 0.00 (s, 3H), −0.01 (s, 3H) ppm.

^{13}C-NMR (101 MHz, $CDCl_3$): δ = 176.7, 166.1, 159.1, 135.3, 133.1, 130.8, 130.4, 129.8, 129.5, 128.6, 119.6, 113.8, 84.2, 72.6, 72.0, 70.2, 55.4, 42.9, 35.6, 26.2, 18.4, 12.0, −3.7, −4.9 ppm.

IR (Diamond-ATR, neat): ṽ = 2928 (w), 2856 (w), 1716 (s), 1613 (w), 1514 (m), 1452 (w), 1315 (w), 1270 (s), 1248 (s), 1175 (m), 1109 (m), 1069 (m), 1033 (m), 997 (w), 932 (w), 836 (m), 810 (m), 777 (m), 711 (s), 668 (m) cm^{-1}.

HRMS (APCI-TOF) m/z: $[M+Na]^+$ calcd. for $C_{30}H_{42}NaO_7Si^+$: 565.2592; found: 565.2602.

¹H-NMR (400 MHz, $CDCl_3$):

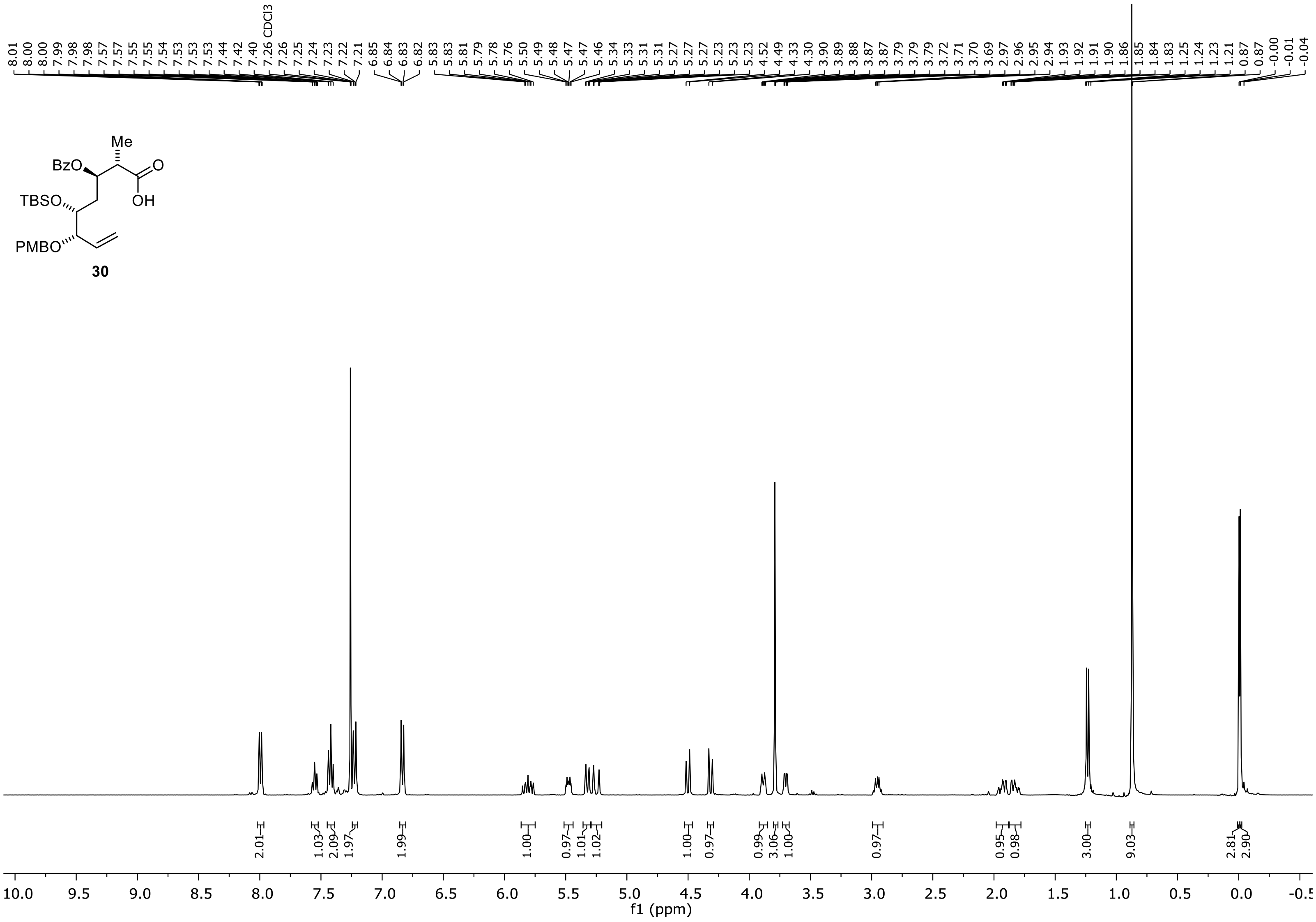

30

^{13}C-NMR (101 MHz, $CDCl_3$):

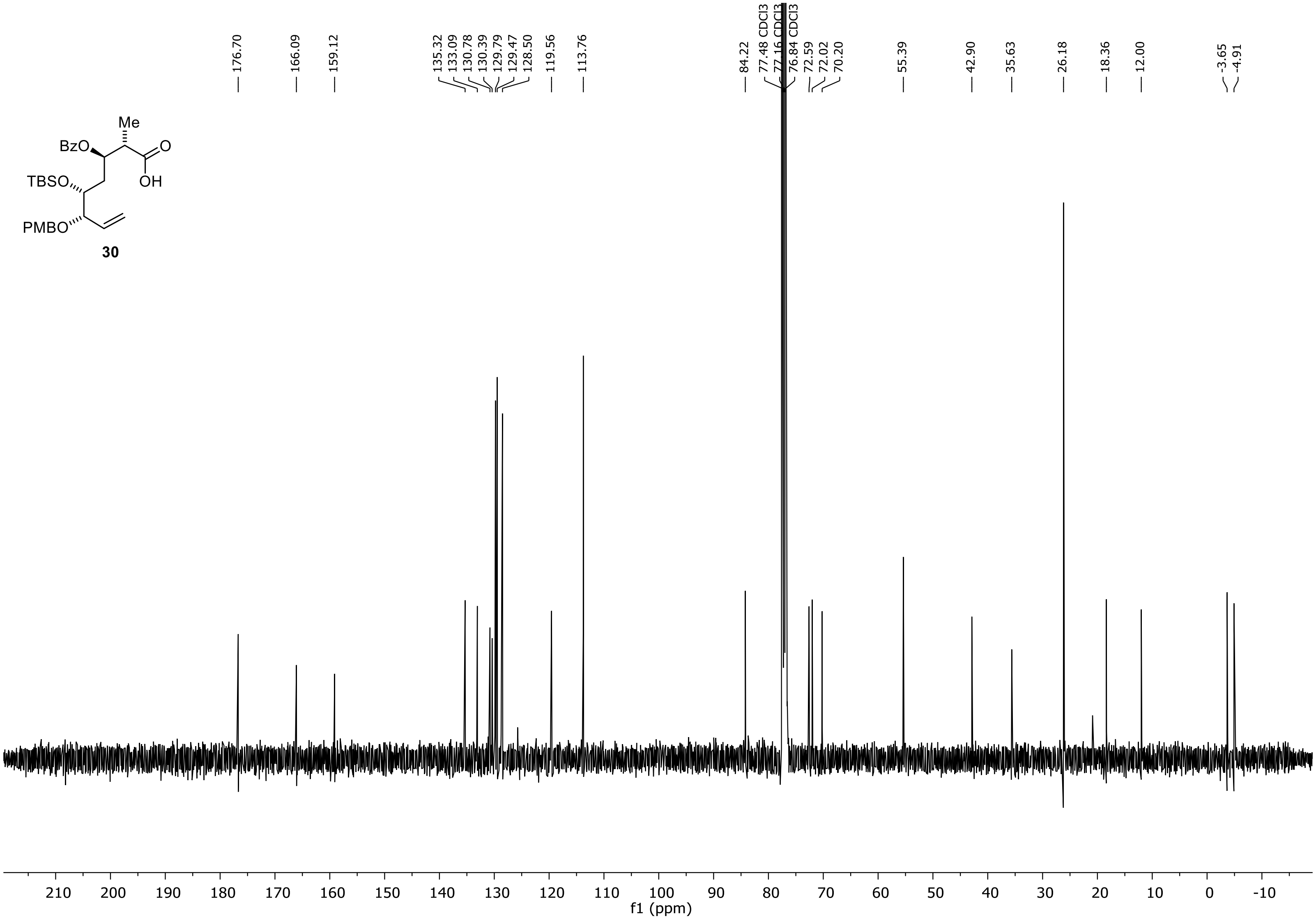

Preparation of 31: Ozonolysis of Alkene 30

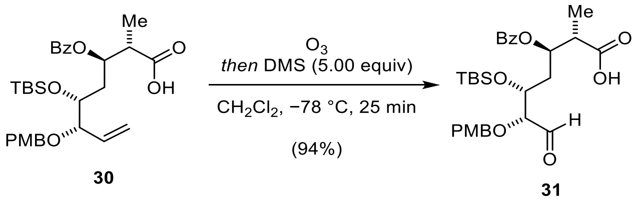

A solution of alkene **30** (4.92 g, 9.07 mmol, 1.00 equiv) in dichloromethane (100 mL) was cooled to −78 °C. An oxygen cylinder was attached to an ozone generator, the outlet was connected to a Pasteur pipette and was placed into this reaction mixture. Ozone was bubbled into the solution for 35 minutes (NOTE 1) until the mixture took a blue hue, indicating saturation of ozone. The ozone generator was switched off immediately and oxygen was bubbled into the solution until the blue color disappeared. TLC analysis (mini workup in DCM with one drop of DMS, eluent: 50% EtOAc in hexanes) indicated complete consumption of starting material. Dimethyl sulfide (3.4 mL, 46.0 mmol, 5.07 equiv) was added and the reaction mixture was slowly warmed to room temperature over the course of 1. The volatiles were removed under reduced pressure using a rotary evaporator inside a fume hood. The residue was purified over silica gel by MPLC Combi flash column chromatography (30 to 100% EtOAc in hexanes) which afforded aldehyde **31** (4.64 g, 8.52 mmol, 94%) as a clear viscous oil.

NOTE 1: The reaction time is dependent on the scale the ozonolysis was performed on. The appearance of the faint blue hue is always the ideal indicator for completion of the reaction. Longer reaction times need to be avoided as extensive exposure to ozone will initiate the formation of side products.

Characterization Data for 31:

R_f (70% ethyl acetate in hexanes) = 0.45 (UV, CAM, DNP).

^{1}H-NMR (400 MHz, $CDCl_3$): δ = 9.64 (d, *J* = 1.9 Hz, 1H), 8.02 – 7.98 (m, 2H), 7.58 – 7.52 (m, 1H), 7.42 (d, *J* = 7.7 Hz, 2H), 7.25 – 7.21 (m, 2H), 6.88 – 6.82 (m, 2H), 5.43 (dt, *J* = 8.6, 4.3 Hz, 1H), 4.59 (d, *J* = 11.4 Hz, 1H), 4.54 (d, *J* = 11.4 Hz, 1H), 4.19 – 4.12 (m, 1H), 3.79 (s, 3H), 3.74 (t, *J* = 2.4 Hz, 1H), 2.94 (qd, *J* = 7.1, 4.4 Hz, 1H), 2.01 (dt, *J* = 8.9, 4.1 Hz, 2H), 1.24 (d, *J* = 7.1 Hz, 3H), 0.87 (s, 9H), 0.04 (s, 3H), 0.02 (s, 3H) ppm.

^{13}C-NMR (101 MHz, $CDCl_3$): δ = 203.5, 175.6*, 166.3, 159.6*, 133.3, 130.1, 129.9, 129.8, 129.3, 128.6, 114.1, 86.8, 77.4, 73.0, 72.1, 71.1, 55.4, 36.0, 26.0, 18.2, 12.1, −4.0, −5.0 ppm.

*Signals were deduced from HSQC and HMBC analysis.

IR (Diamond-ATR, neat): ṽ = 2929 (w), 2856 (w), 1776 (m), 1701 (m), 1612 (w), 1585 (w), 1453 (w), 1382 (m), 1272 (m), 1247 (s), 1240 (m), 1176 (m), 1107 (s), 1070 (m), 1026 (m), 970 (m), 837 (s), 778 (m) cm^{-1}.

HRMS (ESI-TOF) m/z: $[M+H_2O+Na]^+$ calcd. for $C_{29}H_{38}NaO_7Si^+$: 549.2279; found: 549.2259.

^{1}H-NMR (400 MHz, $CDCl_3$):

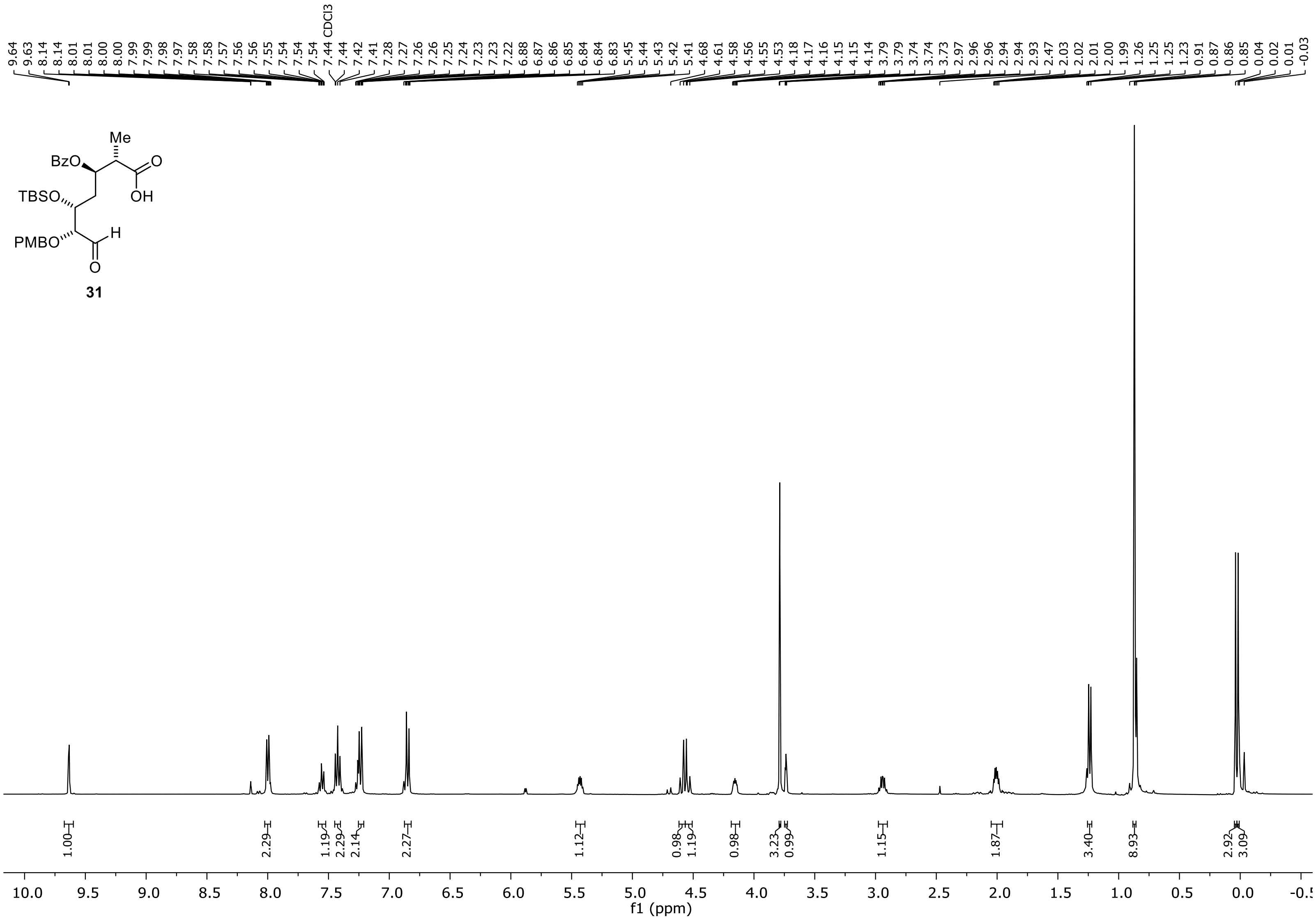

^{13}C-NMR (101 MHz, $CDCl_3$):

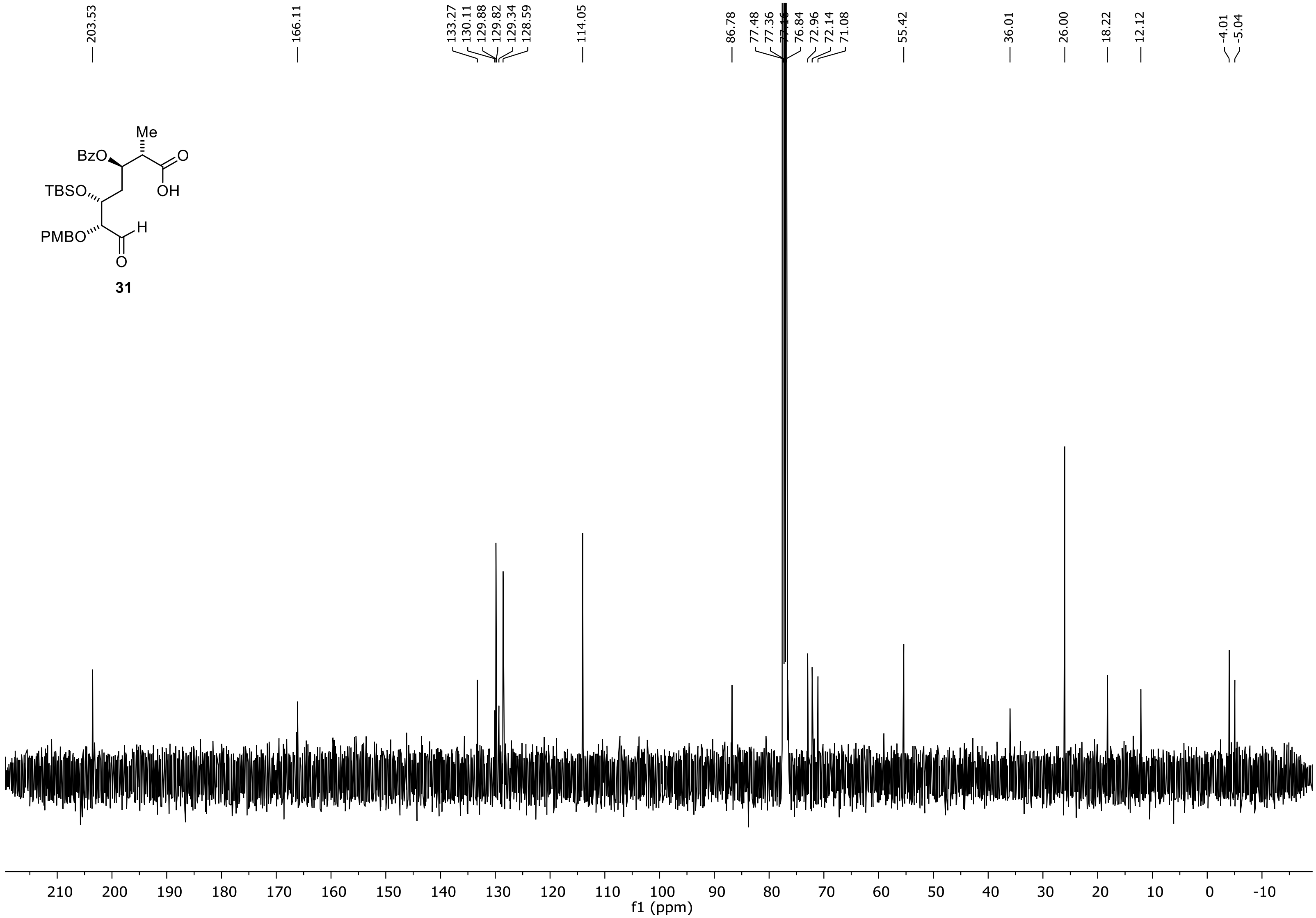

31

Preparation of 32: Esterification of Carboxylic Acid 31 with Alcohol 19

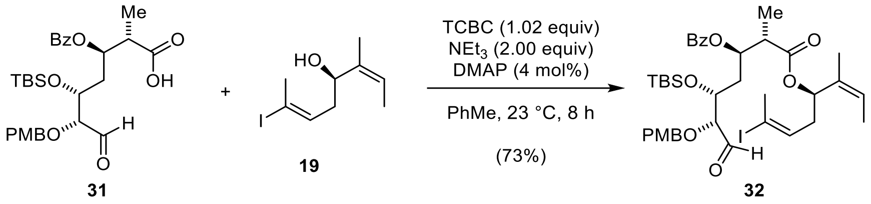

To a stirred solution of carboxylic acid **31** (4.46 g, 8.19 mmol, 1.00 equiv) in anhydrous toluene (70 mL) was added triethylamine (2.3 mL, 16.4 mmol, 2.00 equiv) and Yamaguchi's reagent (TCBC) (2.03 g, 8.32 mmol, 1.02 equiv) at 0 °C. The reaction mixture was stirred at 0 °C for 1 h. TLC-analysis (20% EtOAc in hexanes, UV, CAM) (NOTE 1) indicated complete consumption of carboxylic acid **31** and formation of the anhydride electrophile. A solution of alcohol **19** (2.28 g, 8.57 mmol, 1.05 equiv) in toluene (3 mL+2 mL rinse) was added, followed by a solution of DMAP (40.0 mg, 0.328 mmol, 4 mol%) in toluene (1 mL). The reaction mixture was kept in the ice bath but was slowly allowed to warm to ambient temperature. After 8 h TLC-analysis (20% EtOAc in hexanes, UV, CAM) (NOTE 1) indicated complete consumption of the anhydride intermediate and aqueous saturated sodium bicarbonate solution (50 mL) was added. The aqueous phase was extracted with ethyl acetate (3 x 100 mL). The combined organic layers were washed with aqueous saturated sodium chloride solution (200 mL), dried over anhydrous magnesium sulfate, filtered and concentrated under reduced pressure. The crude product was subjected to silica gel flash column chromatography (1 to 15% EtOAc in hexanes) affording the desired ester **32** (5.24 g, 5.95 mmol, 73%) (NOTE 2) as a yellow viscous oil.

NOTE 1: One drop of the reaction mixture was diluted in a 1 mL vial with anhydrous dichloromethane and the vial was kept as a reference TLC sample.

NOTE 2: The product was kept in an amber glass vial, or a glass vial wrapped in aluminum foil for storage within the freezer to prevent photoisomerization or deiodination.

Characterization Data for 32:

R_f (20% EtOAc in hexanes) = 0.63 (UV, CAM).

$[\alpha]_D^{26}$ = +32.6° (c = 2, $CHCl_3$).

^{1}H-NMR (400 MHz, $CDCl_3$): δ = 9.63 (dd, *J* = 7.2, 1.9 Hz, 1H), 7.99 (ddd, *J* = 7.8, 6.3, 1.4 Hz, 2H), 7.59 – 7.51 (m, 1H), 7.48 – 7.41 (m, 2H), 7.26 – 7.21 (m, 2H), 6.88 – 6.81 (m, 2H), 5.96 (ddt, *J* = 7.7, 6.2, 1.5 Hz, 1H), 5.63 – 5.54 (m, 1H), 5.48 – 5.40 (m, 1H), 5.40 – 5.33 (m, 1H), 4.63 – 4.52 (m, 2H), 4.15 (dt, *J* = 8.9, 2.9 Hz, 1H), 3.80 (d, *J* = 2.9 Hz, 3H), 3.75 – 3.71 (m, 1H), 2.94 – 2.87 (m, 1H), 2.41 – 2.27 (m, 4H), 2.20 – 2.09 (m, 1H), 2.05 – 1.86 (m, 2H), 1.62 (dt, *J* = 7.1, 1.5 Hz, 3H), 1.55 (dd, *J* = 3.2, 1.7 Hz, 3H), 1.21 (dd, *J* = 6.2, 3.3 Hz, 3H), 0.87 (s, 9H), 0.05 (2 x s, 6H) ppm.

^{13}C-NMR (101 MHz, $CDCl_3$): δ = 203.6, 172.5, 165.9, 159.6, 135.6, 133.2, 132.1, 130.2, 130.0, 129.84, 129.80, 129.4, 128.6, 124.8, 114.0, 86.9, 72.9, 72.3, 71.1, 71.0, 55.4, 43.4, 36.0, 33.0, 27.8, 26.1, 18.3, 17.9, 13.3, 12.0, −4.0, −5.0 ppm.

IR (Diamond-ATR, neat): ṽ = 2929 (m), 2857 (m), 2361 (w), 1724 (s), 1612 (m), 1514 (m), 1452 (m), 1379 (m), 1361 (m), 1271 (s), 1250 (s), 1176 (m), 1111 (s), 1069 (m), 1027 (m), 837 (m), 778 (m) cm^{-1}.

HRMS (APCI-TOF) m/z: $[M+H]^+$ calcd. for $C_{38}H_{54}IO_8Si^+$: 793.2620; found: 793.2627.

¹H-NMR (400 MHz, $CDCl_3$):

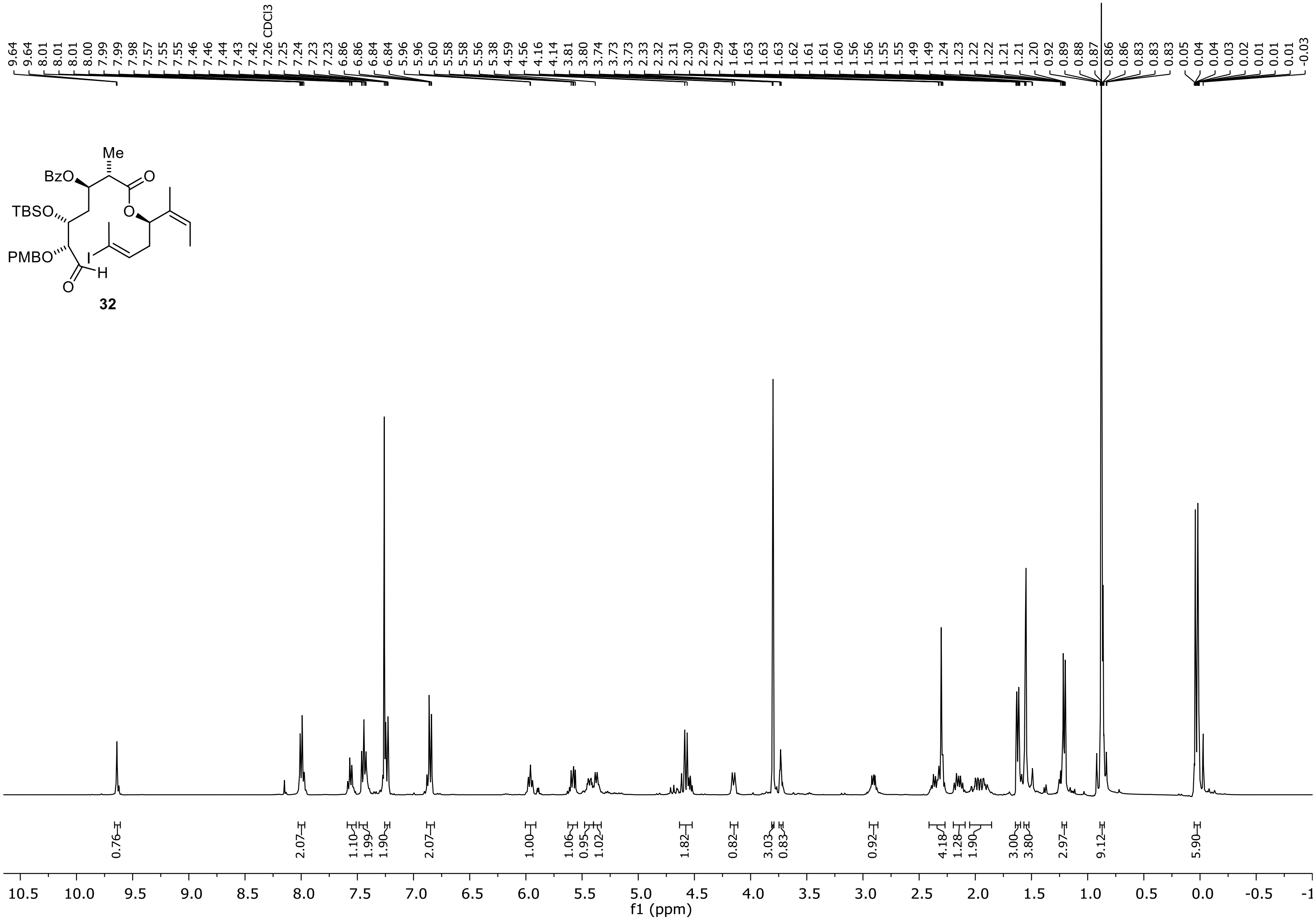

^{13}C-NMR (101 MHz, $CDCl_3$):

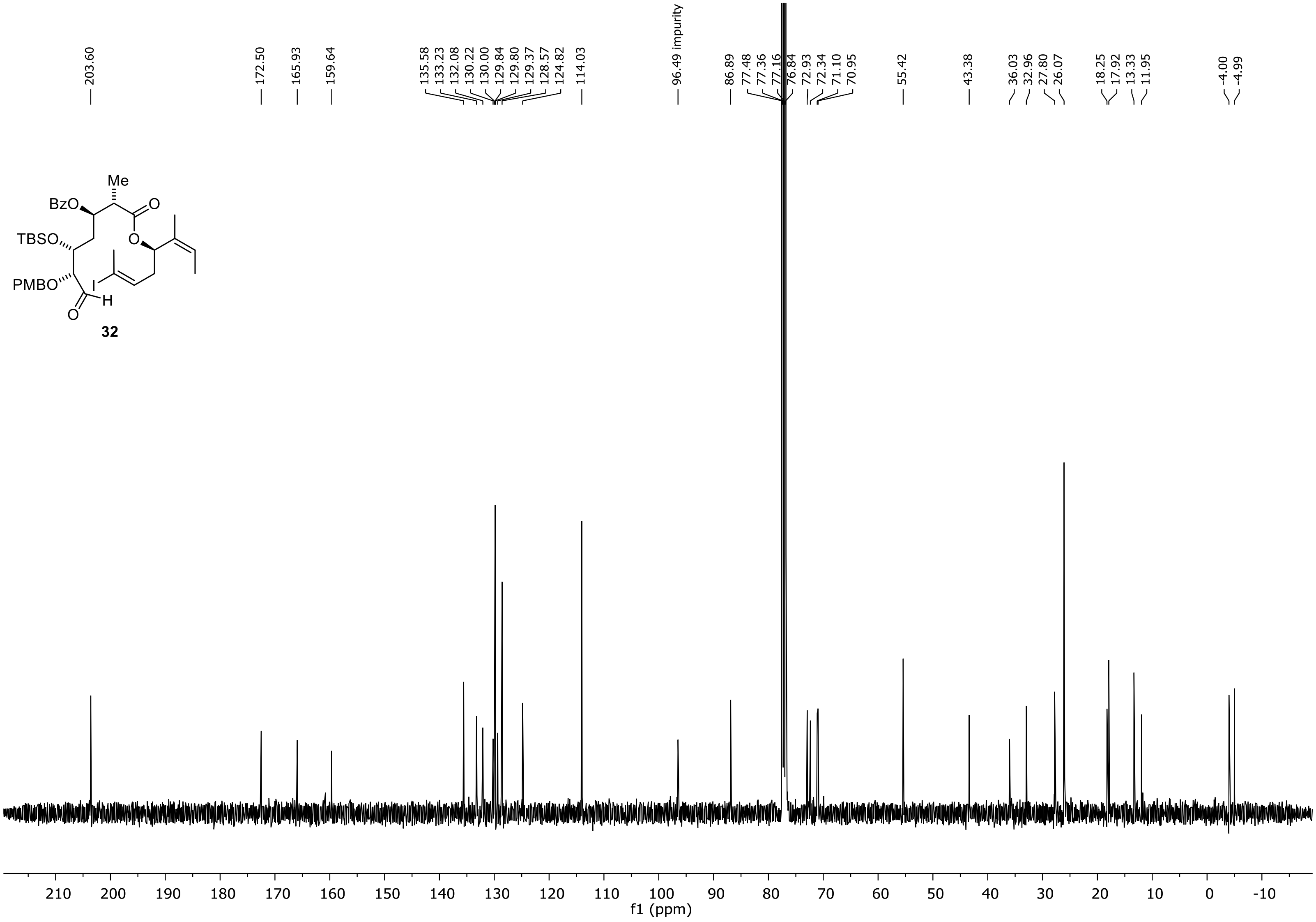

Preparation of 33: NHTK Macrocyclization of Precursor 32

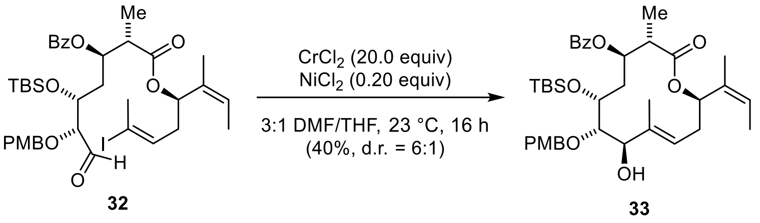

A 3:1 mixture of DMF/THF (180 mL, 135 mL + 45 mL) was degassed by three consecutive freeze-pump-thaw cycles. Simultaneously, a 250 mL Schlenck flask was charged with a stir bar and 4 Å molecular sieves (25 g) and was flame dried extensively under vacuum. After it cooled to room temperature, the flask was transferred into a glovebox and anhydrous chromium(II) chloride (4.31 g, 35.1 mmol, 20.0 equiv) and nickel(II) chloride (45.4 mg, 0.351 mmol, 0.20 equiv) were weighed into the reaction flask. The flask was capped with a rubber septum, was removed from the glovebox and attached to an inert atmosphere Schlenck line. The degassed DMF/THF solvent mixture (160 mL) was added to the reaction flask resulting in a turquoise color to appear. A solution of the macrocyclization precursor **32** (1.39 g, 1.75 mmol, 1.00 equiv) in the remaining degassed solvent mixture (20 mL) was slowly added to the reaction flask by use of a syringe pump over the course of 2 h. The dropwise addition initiated a slow change of color towards a dark green. After the complete addition, stirring was continued for 16 h (NOTE 1) when TLC analysis (20% acetone in hexanes) indicated complete consumption of the starting material. The reaction mixture was filtered over a short celite pad and partitioned between an aqueous saturated ammonium chloride solution (150 mL) and EtOAc (200 mL). The phases were separated, and the aqueous layer was extracted with EtOAc (2 x 150 mL). The combined organic phases were washed successively with water (100mL), 10% aqueous lithium chloride solution (100 mL) and brine (100 mL), dried over anhydrous magnesium sulfate, filtered, and concentrated. Two subsequent flash column chromatography purifications over silica gel (1st using a 5 to 20% EtOAc in hexanes gradient, 2nd using a 5 to 20% acetone in

hexanes gradient) afforded the pure macrocyclic alcohol **33** (471 mg, 706 µmol, 40%) (NOTE 2) as a pale-yellow foam.

NOTE 1: Sometimes the reaction slowed down significantly and stirring was continued for 3–5 days.

NOTE 2: The product was obtained as a mixture of macrocyclic conformers. NMR measurements needed to be conducted at higher temperatures (usually in benzene-d6 at 70 °C) to converge the atropoisomeric signals.

Characterization Data for 33:

R$_f$ (20% EtOAc in hexanes) = 0.35 (UV, CAM).

$[\boldsymbol{\alpha}]_{\mathbf{D}}^{\mathbf{26}}$ = –57.0° (c = 2, $CHCl_3$).

^{1}H-NMR (400 MHz, C_6D_6, 60 °C): δ = 8.15 – 8.11 (m, 2H), 7.22 – 7.07 (m, 5H*), 6.74 – 6.68 (m, 2H), 6.09 (dd, *J* = 11.7, 2.5 Hz, 1H), 5.70 (d, *J* = 11.2 Hz, 1H), 5.58 (dd, *J* = 10.4, 7.5 Hz, 1H), 5.24 (q, *J* = 6.3 Hz, 1H), 4.60 (s, 1H), 4.56 – 4.49 (m, 1H), 4.45 (dd, *J* = 12.4, 2.4 Hz, 1H), 4.24 (d, *J* = 11.3 Hz, 1H), 3.32 (s, 3H), 3.26 (d, *J* = 8.9 Hz, 1H), 3.11 – 3.00 (m, 1H), 2.89 (dd, *J* = 14.6, 11.6 Hz, 1H), 2.84 – 2.76 (m, 1H), 2.56 (s, 1H), 1.95 – 1.85 (m, 1H), 1.82 (t, *J* = 1.5 Hz, 3H), 1.70 – 1.66 (m, 6H), 1.63 (d, *J* = 7.6 Hz, 1H), 1.36 (s, 3H), 1.18 – 1.14 (s, 9H),0.42 (s, 3 H), 0.34 (s, 3H) ppm.

**Overintegrating due to aromatic signals overlapping with the NMR solvent.*

^{13}C-NMR (101 MHz, C_6D_6, 25 °C): δ = 172.3, 166.2, 159.9, 137.9, 133.8, 133.1, 130.0, 129.9, 128.7, 128.2, 127.9, 127.4, 123.8, 114.2, 84.1, 76.5, 73.5, 73.0, 72.5, 69.4, 54.7, 32.3, 30.2, 26.7, 26.4, 23.2, 19.1, 18.5, 13.1, 11.9, –2.84, –5.04 ppm.

IR (Diamond-ATR, neat): ṽ = 3550 (br, w), 2928 (m), 2857 (m), 2362 (w), 1724 (s), 1613 (w), 1586 (w), 1514 (m), 1452 (m), 1378 (w), 1270 (s), 1250 (s), 1167 (s), 1109 (s), 1069 (s), 1033 (s), 917 (m), 869 (m), 837 (m), 778 (m) cm^{-1}.

HRMS (APCI-TOF) m/z: $[M+H]^+$ calcd. for $C_{38}H_{55}O_8Si^+$: 667.3661; found: 667.3652.

¹H-NMR (400 MHz, C_6D_6), 25 °C:

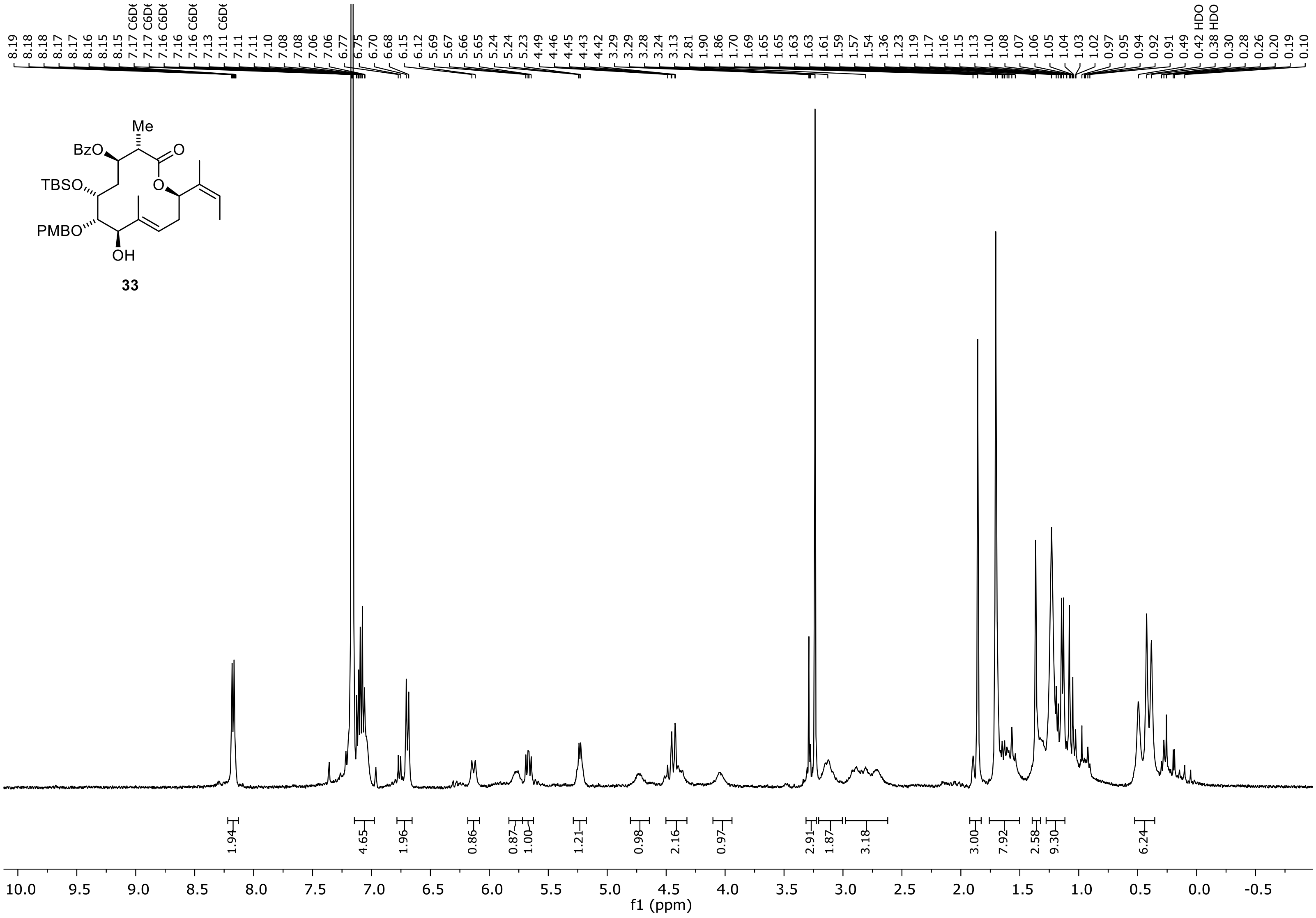

¹H-NMR (400 MHz, C_6D_6), 60 °C:

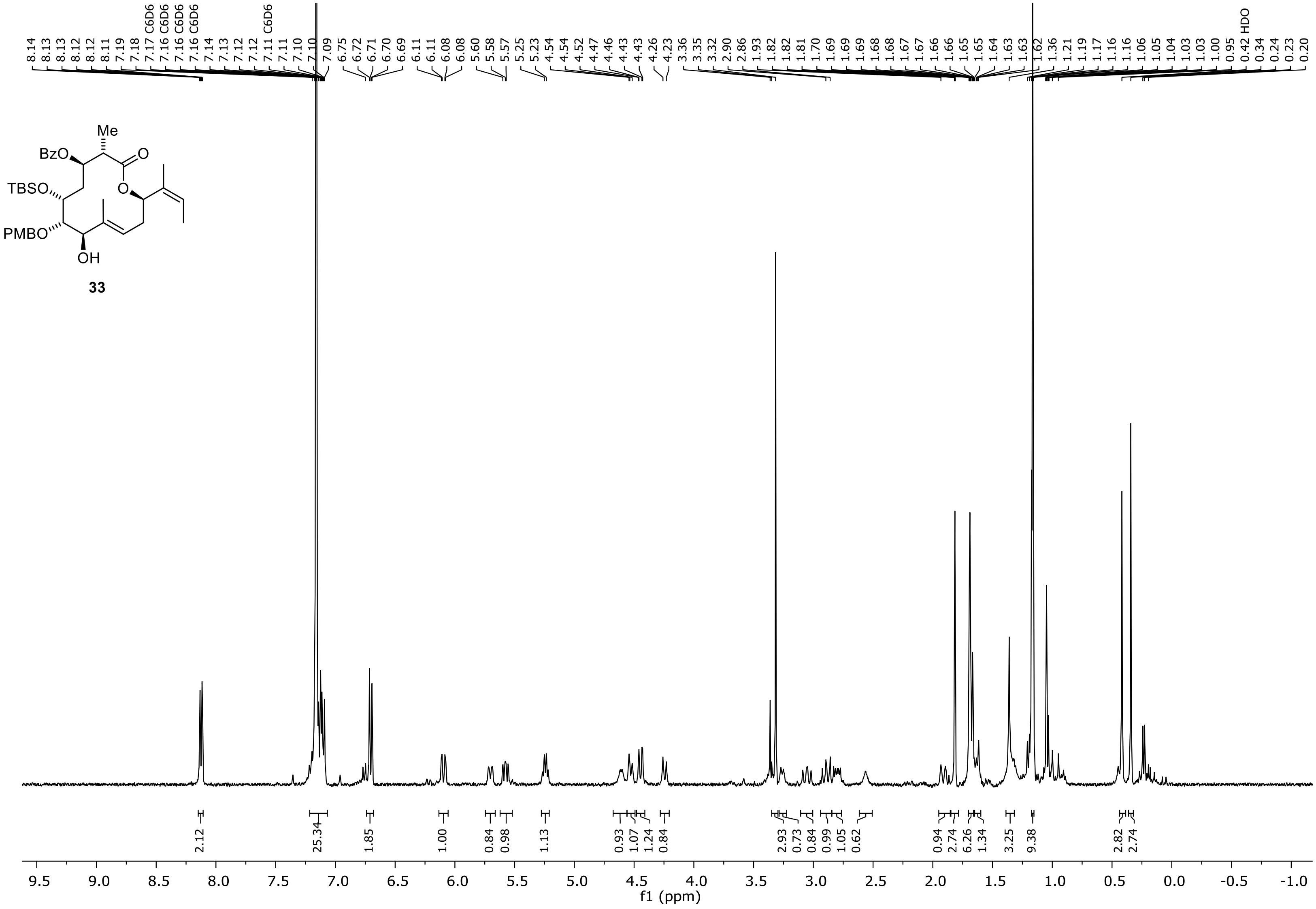

^{13}C-NMR (101 MHz, C_6D_6), 25 °C:

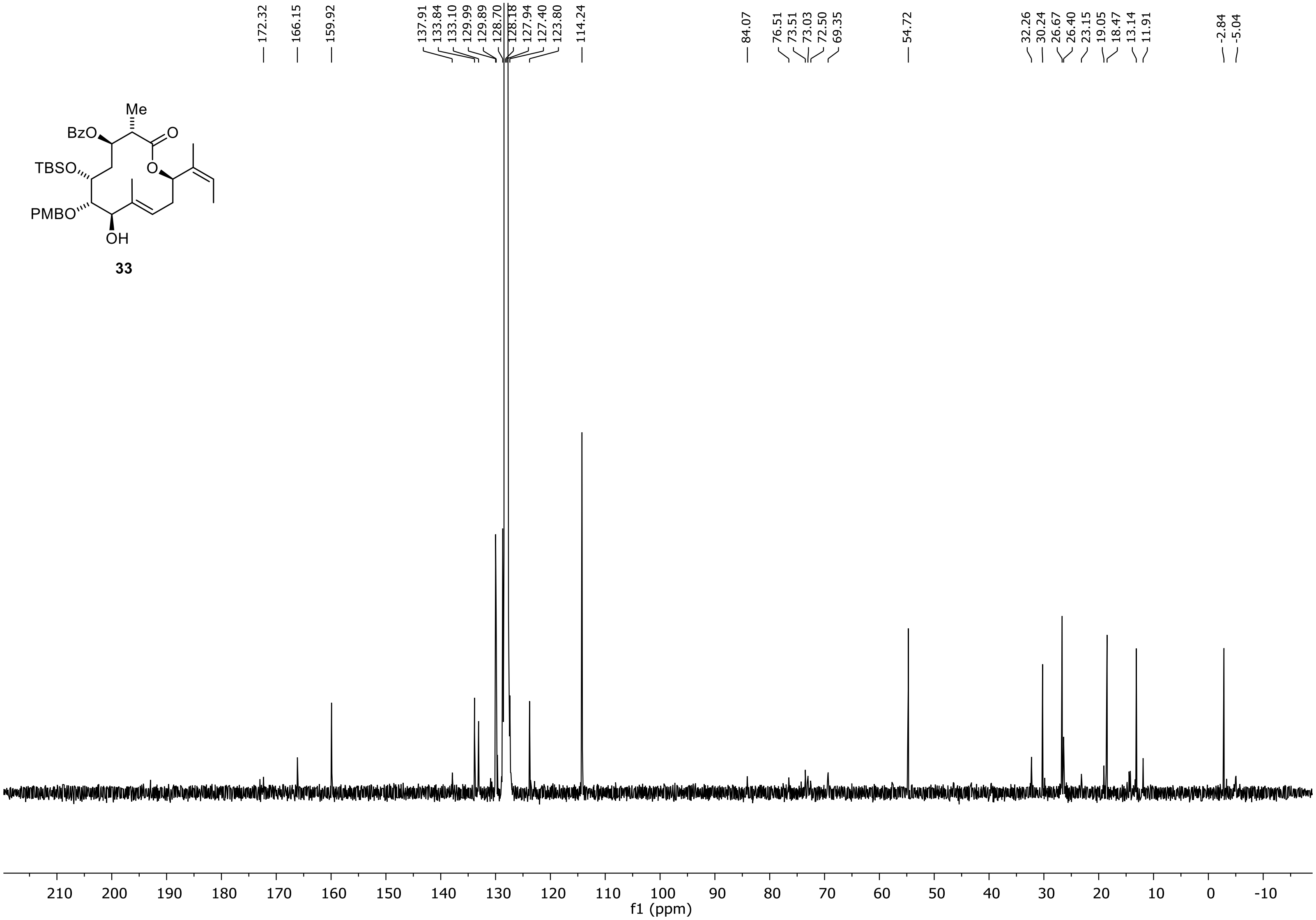

33

Preparation of 34: Schmidt Glycosylation of Allylic Alcohol 33

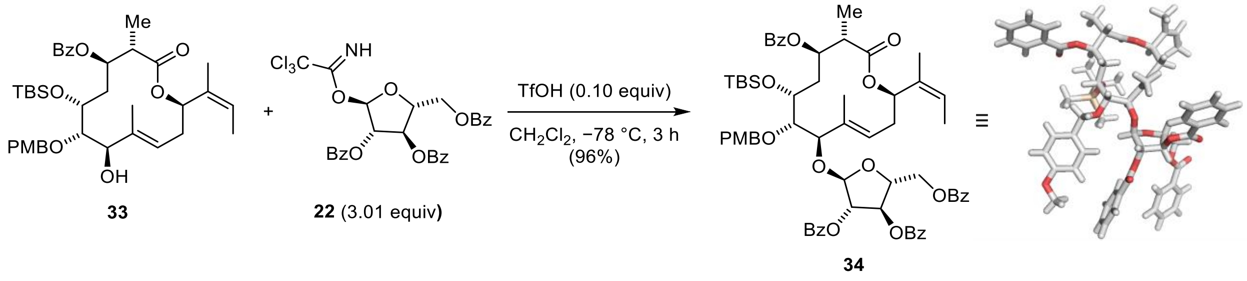

A 10 mL Schlenck tube was charged with a stir bar and 4 Å molecular sieves (100 mg) and was flame dried extensively under vacuum. After it cooled to room temperature, the Schlenck tube was charged with macrocycle **33** (44.5 mg, 66.7 μmol, 1.00 equiv) and the arabinose donor **22** (122 mg, 201 μmol, 3.01 equiv). Dichloromethane (1 mL) was added, and the resultant suspension was stirred at room temperature (23 °C) for 10 min before it was cooled to −78 °C. Triflic acid (TfOH, 0.6 μL, 6.8 μmol, 0.10 equiv) (NOTE 1) was added and the reaction was stirred at −78°C for 3 h when TLC analysis (15 % acetone in hexanes) (NOTE 2) indicated completion. The reaction mixture was neutralized by dropwise addition of triethylamine (1 mL) at −78 °C and the quenched solution is slowly warmed to room temperature (23 °C). The mixture was diluted with DCM (5 mL), was filtered over a short celite pad and was partitioned between an aqueous saturated sodium bicarbonate solution (10 mL) and EtOAc (10 mL). The phases were separated, and the aqueous layer was extracted with EtOAc (2 x 10 mL). The combined organic phases were washed with brine (10 mL), dried over anhydrous magnesium sulfate, filtered, and concentrated under reduced pressure. Purification over silica gel by flash column chromatography (5 to 20% acetone in hexanes) yielded the glycosylated product **34** (70.9 mg, 63.8 μmol, 96%) (NOTE 3) as a viscous clear oil which solidified upon storage in the freezer (NOTE 4).

NOTE 1: A 1% stock solution of TfOH (10 μL) in DCM (990 μL) was prepared freshly and 60 μL of this stock solution was added to the reaction mixture. The equivalence of TfOH should be kept as described as higher concentration of the acid result in significant decomposition of the product.

NOTE 2: The starting material and product are copolar if EtOAc in hexanes is used as the solvent system to monitor reaction progress. In 15% acetone in hexanes the starting material has an R_f = 0.41 and the glycosylated product R_f = 0.37.

NOTE 3: The product was obtained as a mixture of macrocyclic conformers. NMR measurements needed to be conducted at higher temperatures (usually in toluene-d_8 at 85 °C) to converge the signals.

NOTE 4: Crystals of glycosylated macrolactone **34** suitable for X-ray analysis were prepared as follows: In a 1 mL vial, approximately 10 mg of compound were dissolved in minimal amounts of benzene (90 μL). This opened vial was placed inside a larger 20 mL scintillation vial containing 2 mL of hexanes. The larger vial was sealed with a screw cap and placed inside a 4 °C fridge. Slow diffusion of hexanes into the solution of **34** resulted in the growth of needle shaped crystals over the course of 3 days. The crystal was collected and carefully washed with hexanes. The colorless block-like specimen of $C_{67}H_{77}O_{15}Si$, approximate dimensions 0.350 mm x 0.540 mm x 0.560 mm, was used for the X-ray crystallographic analysis. The X-ray intensity data were measured on a Bruker APEX-II CCD system equipped with a graphite monochromator and a Mo sealed tube (λ = 0.71073 Å).

Characterization Data for 34:

$\mathbf{R}_f$ (15% acetone in hexanes) = 0.36 (UV, CAM).

$[\boldsymbol{\alpha}]_{\mathbf{D}}^{\mathbf{25}}$ = −11.1° (c = 2, $CHCl_3$).

^{1}H-NMR (400 MHz, toluene-d_8, 85 °C): δ = 8.04 (td, *J* = 5.8, 4.9, 3.1 Hz, 6H), 8.00 – 7.95 (m, 2H), 7.23 (d, *J* = 8.2 Hz, 2H), 7.19 – 7.01 (m, 12H*), 6.63 – 6.56 (m, 2H), 5.95 (dd, *J* = 11.4, 2.4 Hz, 1H), 5.88 (d, *J* = 1.5 Hz, 1H), 5.76 (s, 1H), 5.70 (d, *J* = 10.8 Hz, 1H), 5.62 – 5.56 (m, 1H), 5.46 (t, *J* = 8.8 Hz, 1H), 5.20 (d, *J* = 6.1 Hz, 1H), 4.83 (q, *J* = 6.8 Hz, 1H), 4.75 – 4.57 (m, 5H), 4.35 (d, *J* = 11.9 Hz, 1H), 3.54 (s, 1H), 3.26 (s, 3H), 2.96 (t, *J* = 13.8 Hz, 1H), 2.74 (q, *J* = 11.8 Hz, 2H), 1.89 – 1.78 (m, 4H), 1.68 – 1.58 (m, 7H), 1.13 (d, *J* = 6.6 Hz, 3H), 1.07 (s, 9H) ppm.**

**Overintegrating due to aromatic signals overlapping with the NMR solvent.*

***The NMR-instrument mistakenly locked the wrong signal for toluene-d_8 and signals below 0.73 ppm were cut off, hence the $R_2Si(CH_3)_2$ signals are missing.*

^{13}C-NMR (101 MHz, C_6D_6, 25 °C): δ = 172.3, 166.1, 166.0, 165.8, 165.5, 159.6, 136.9, 133.7, 133.4, 133.2, 133.0, 132.9, 130.7, 130.32, 130.28, 130.22, 130.16, 130.09, 130.00, 129.97, 129.8, 129.2, 128.6, 128.5, 128.2, 127.9, 123.8, 114.2, 114.0, 107.0, 83.79, 82.75, 82.2, 81.9, 79.6, 73.4, 73.2, 71.4, 64.7, 54.5, 46.5, 39.4, 32.0, 30.2, 26.8, 19.1, 18.4, 14.1, 13.3, 12.6, −2.4, −5.3 ppm.

IR (Diamond-ATR, neat): ṽ = 2924 (br, m), 2851 (m), 2360 (w), 2336 (w), 1725 (s), 1514 (w), 1452 (m), 1315 (w), 1269 (s), 1170 (m), 1109 (s), 1070 (s), 1028 (m), 968 (m), 840 (w), 810 (w), 776 (w) cm^{-1}.

HRMS (APCI-TOF) m/z: $[M+NH_4]^+$ calcd. for $C_{64}H_{78}NO_{15}Si^+$: 1128.5135; found: 1128.5147.

^{1}H-NMR (400 MHz, C_6D_6), 25 °C:

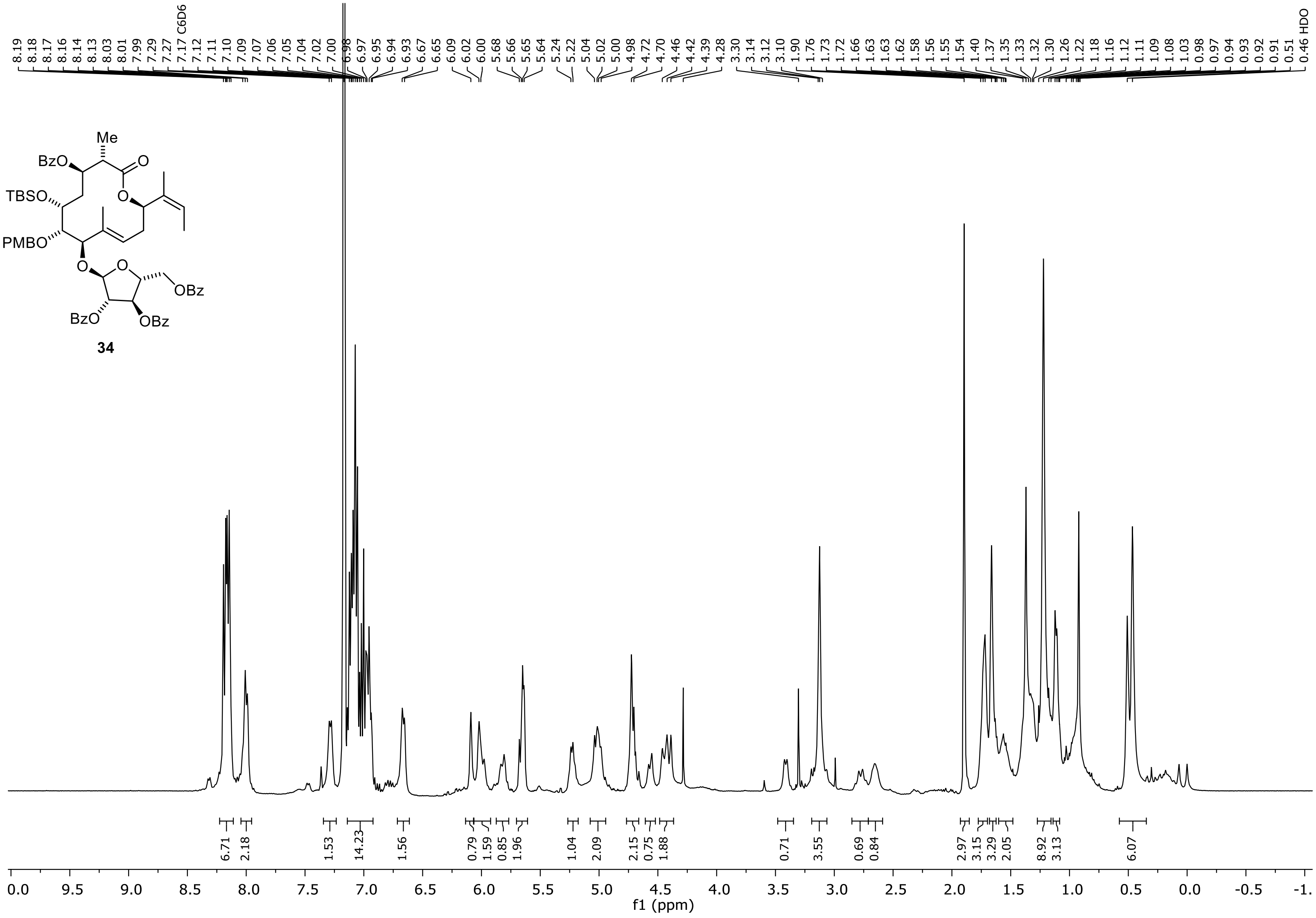

^{1}**H-NMR** (400 MHz, toluene-d$_8$), 85 °C:

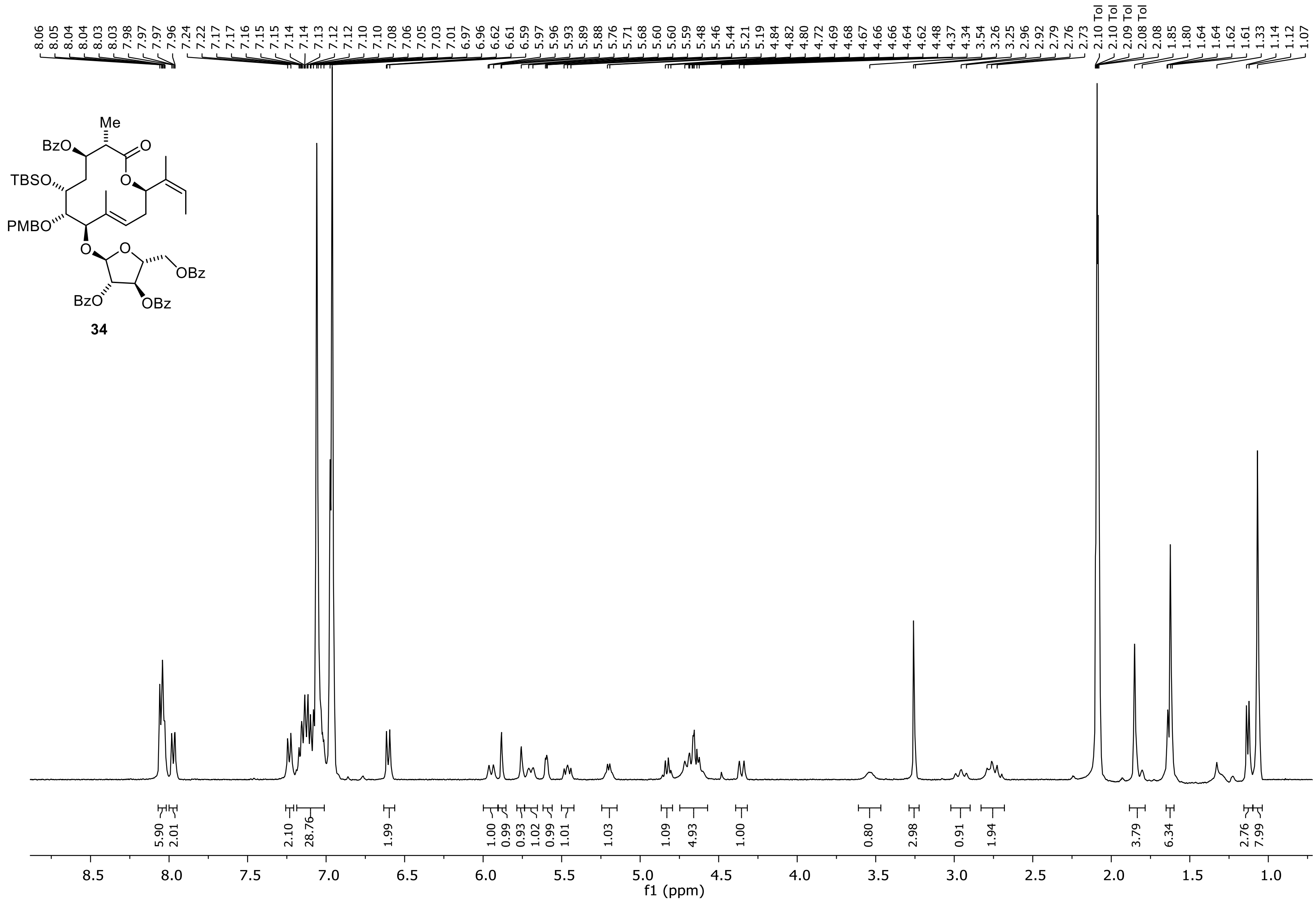

34

^{13}C-NMR (101 MHz, C_6D_6), 25 °C:

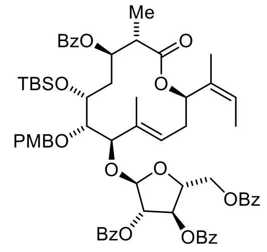

34

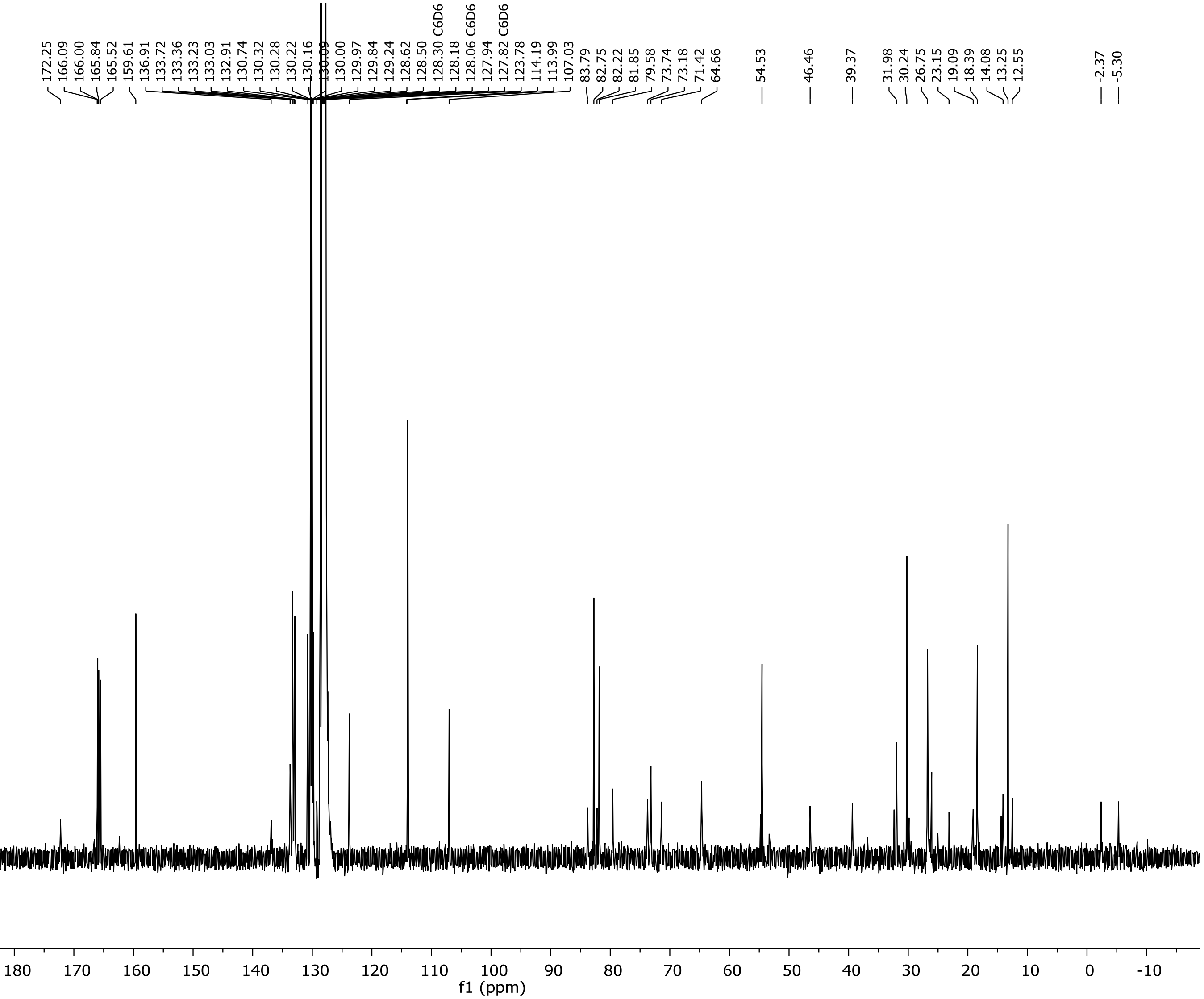

Preparation of 35: PMB Deprotection of Ether 34

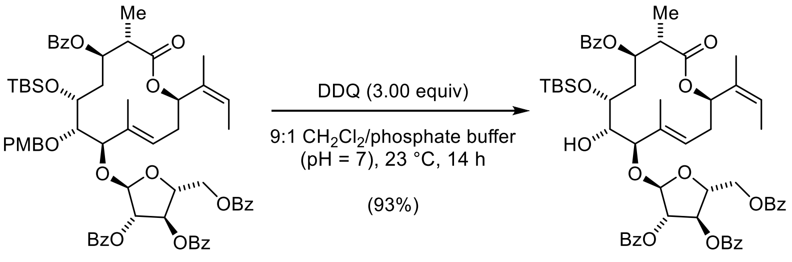

Within a 4 mL vial, the PMB protected macrocycle **34** (65.6 mg, 59.0 μmol, 1.00 equiv) was dissolved in dichloromethane (540 μL) and phosphate buffer (pH = 7, 60 μL). DDQ (40.2 mg, 177 μmol, 3.00 equiv) was added in one portion and the resultant deep orange suspension was stirred at 23 °C for 14 h. TLC analysis (20% acetone in hexanes) (NOTE 1) indicated complete deprotection and was quenched by the addition of aqueous saturated sodium bicarbonate solution (1 mL). The phases were separated, and the aqueous layer was extracted with dichloromethane (2 x 2 mL). The combined organic layers were washed with brine (2 mL), dried over anhydrous sodium sulfate, filtered, and concentrated. The crude product was purified over silica gel by Combi flash MPLC (5 to 20% EtOAc in hexanes) affording the desired secondary alcohol **35** (54.6 mg, 55.1 μmol, 93%) as a colorless viscous oil.

NOTE 1: The starting material and product are copolar if EtOAc in hexanes is used as the solvent system to monitor reaction progress.

Characterization Data for 35:

R$_f$ (20% EtOAc in hexanes) = 0.47 (UV, CAM).

$[\alpha]_D^{25}$ = −23.9° (c = 2, $CHCl_3$).

^{1}H-NMR (400 MHz, $CDCl_3$): δ = 8.08 – 8.01 (m, 8H), 7.60 – 7.49 (m, 4H), 7.43 (p, *J* = 7.9 Hz, 6H), 7.33 (t, *J* = 7.7 Hz, 2H), 5.92 – 5.80 (m, 1H), 5.61 – 5.51 (m, 3H), 5.34 (d, *J* = 5.6 Hz, 2H), 5.30 – 5.23 (m, 1H), 4.78 (dd, *J* = 11.4, 2.8 Hz, 1H), 4.68 – 4.56 (m, 2H), 4.28 (s, 1H), 4.07 (d, *J* = 11.9 Hz, 1H), 3.66 (s, 1H), 2.84 – 2.69 (m, 2H), 1.87 (d, *J* = 14.8 Hz, 1H), 1.73 (s, 3H), 1.71 – 1.61 (m, 7H), 1.13 (d, *J* = 6.8 Hz, 3H), 0.91 (s, 9H), 0.16 (s, 3H), 0.14 (s, 3H) ppm.

^{13}C-NMR (101 MHz, $CDCl_3$): δ = 172.9*, 166.3, 166.1, 165.9, 165.8, 135.5, 133.8, 133.7, 133.4, 133.3, 133.0, 130.5, 130.1, 130.0, 129.9, 129.8, 129.7, 129.1, 128.8, 128.69, 128.66, 128.5, 123.4, 114.5, 106.3, 86.0, 82.6, 81.2, 77.6, 77.4, 74.7*, 73.0*, 72.8*, 70.6*, 64.2, 36.8, 32.0, 29.8, 26.3, 24.8, 23.5, 18.4, 13.2, 12.3, −3.0, −5.4* ppm.

**Signals were identified by analysis of 2D-NMR (HSQC, HMBC).*

IR (Diamond-ATR, neat): ṽ = 2928 (w), 2856 (w), 2360 (w), 2341 (w), 1723 (s), 1602 (w), 1452 (m), 1315 (m), 1268 (s), 1166 (m), 1108 (s) 1069 (s), 1027 (m), 838 (w), 710 (s) cm^{-1}.

HRMS (APCI-TOF) m/z: $[M+H]^+$ calcd. for $C_{56}H_{67}O_{14}Si^+$: 991.4295; found: 991.4224.

^{1}H-NMR (400 MHz, $CDCl_3$):

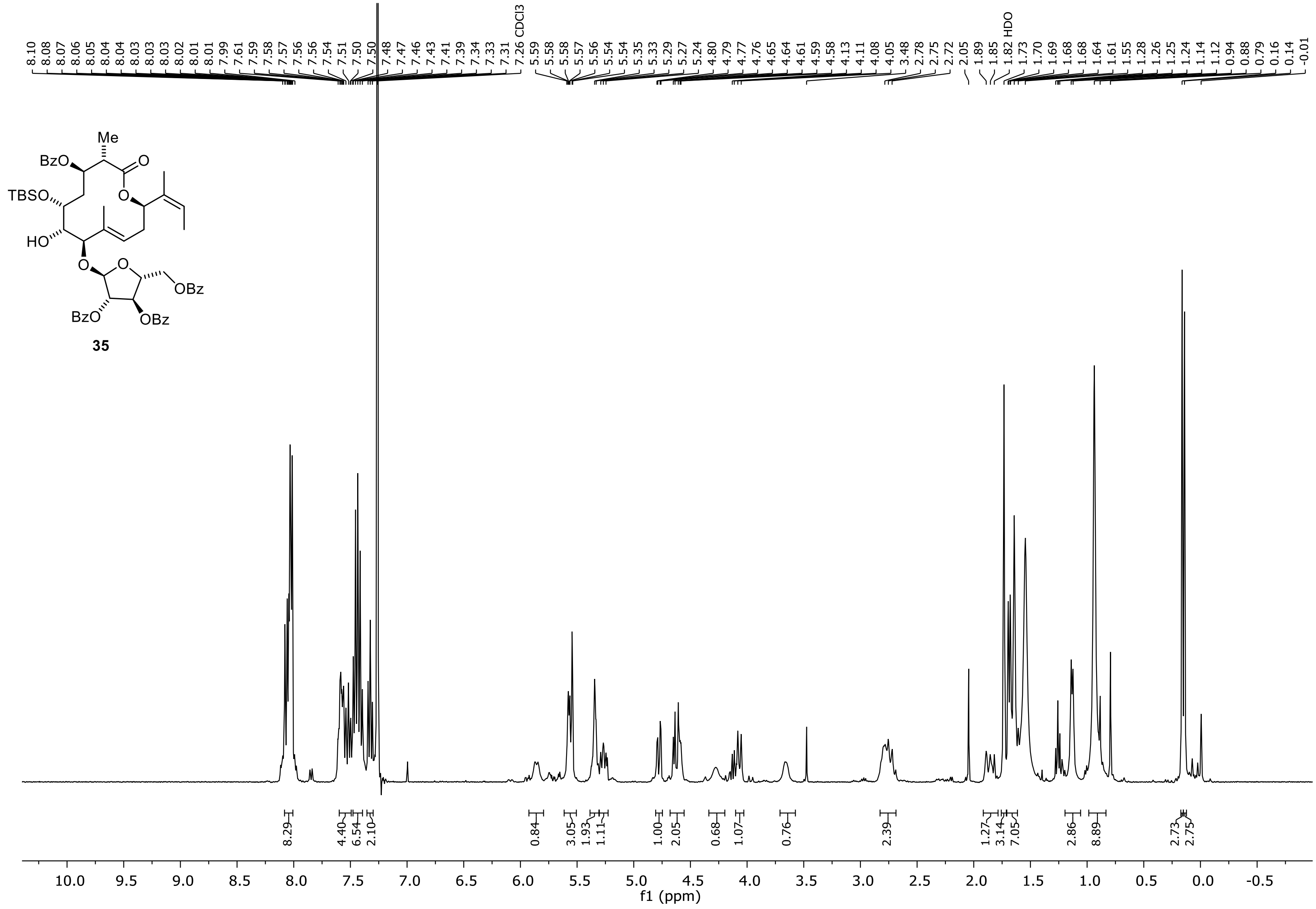

^{13}C-NMR (101 MHz, $CDCl_3$):

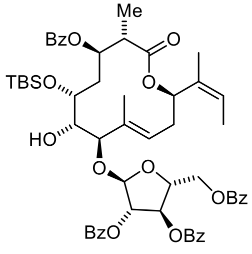

35

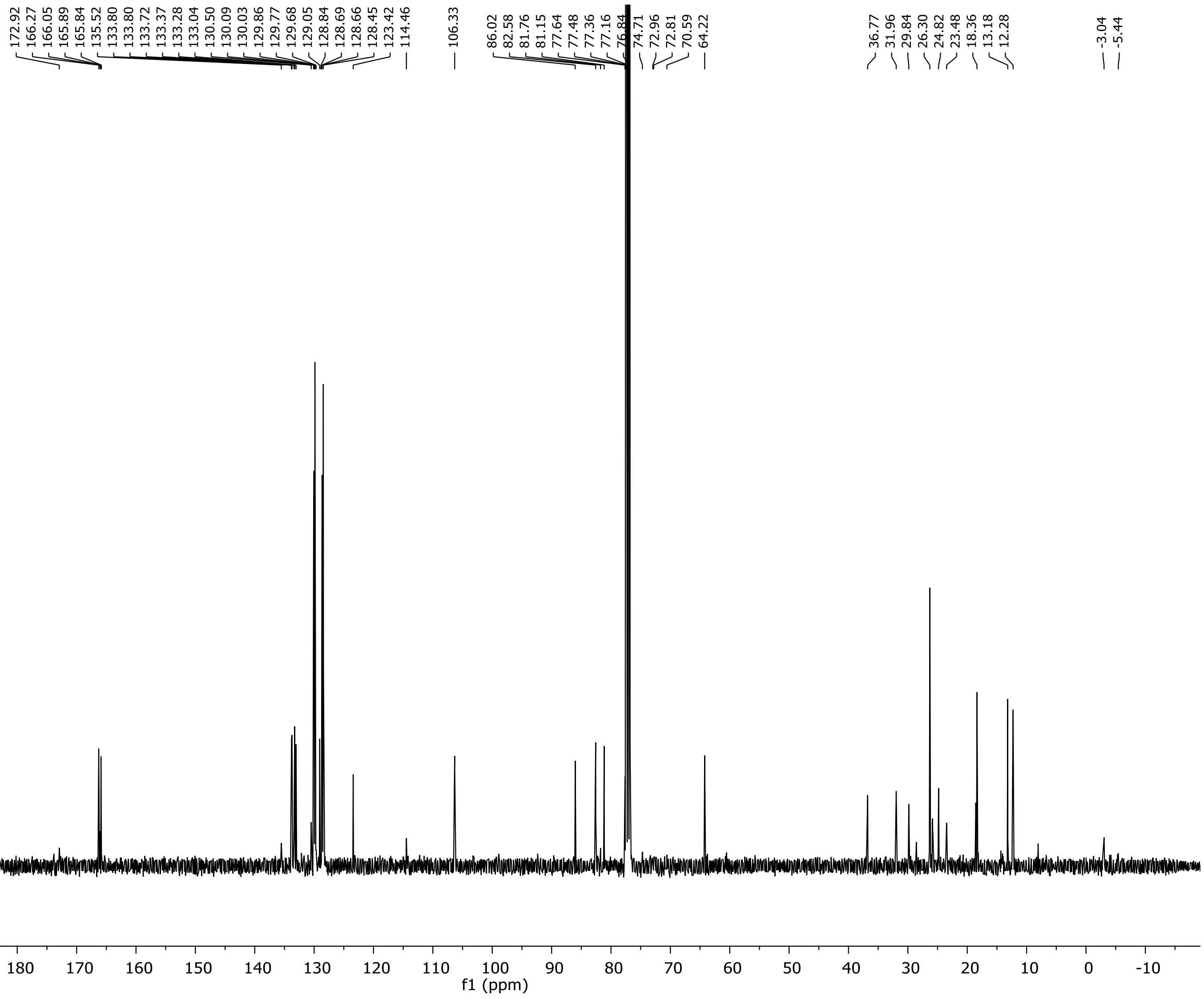

HSQC (400 MHz, CDCl3):

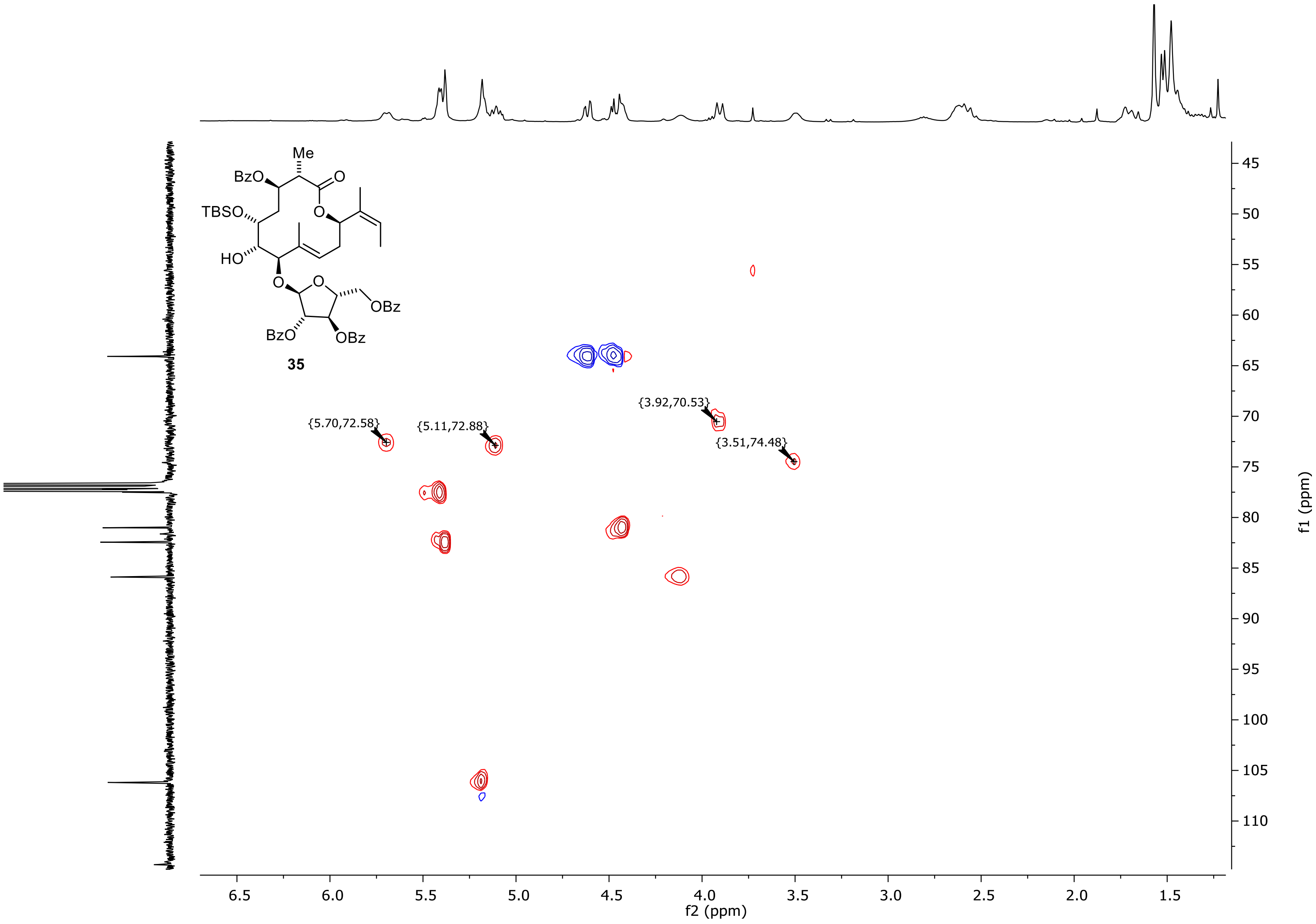

Preparation of 36: Isovaleryl Ester Formation with Alcohol 35

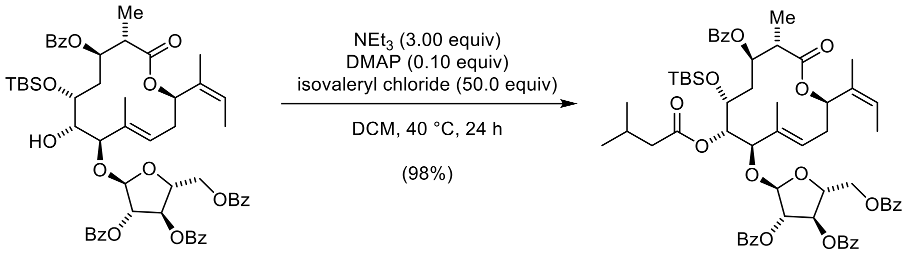

35 **36**

To a solution of the secondary alcohol **35** (163 mg, 164 μmol, 1.00 equiv) in dichloromethane (1 mL) was added isovaleryl chloride (1.0 mL, 8.20 mmol, 49.9 equiv) (NOTE 1) trietylamine (70 μL, 503 mmol, 3.06 equiv) and DMAP (2.0 mg, 16.4 mmol, 0.10 equiv) at room temperature. The reaction mixture was heated to 40 °C using a heating mantle for 24 hours. TLC analysis indicated full conversion and the mixture was partitioned between aqueous saturated sodium bicarbonate solution (5 mL) and ethyl acetate (5 mL). The phases were separated, and the aqueous layer was extracted with EtOAc (2 x 5 mL). The combined organic layers were washed with brine (5 mL), dried over anhydrous, magnesium sulfate, filtered, and concentrated. The crude product was purified over silica gel by Combi flash MPLC (5 to 20% EtOAc in hexanes) affording the isovaleryl ester **36** (173 mg, 161 μmol, 98%) as a clear viscous oil.

NOTE 1: The isovaleryl chloride and the hydrolyzed acid have a notorious smell, and the reaction should be set up in a well vented hood.

Characterization Data for 36:

R_f (30% EtOAc in hexanes) = 0.66 (UV, CAM).

$[\boldsymbol{\alpha}]_{\mathbf{D}}^{\mathbf{25}}$ = −24.0° (c = 2, $CHCl_3$).

^{1}H-NMR (400 MHz, $CDCl_3$): δ = 8.09 (d, *J* = 7.6 Hz, 2H), 8.03 – 7.99 (m, 6H), 7.63 – 7.39 (m, 10H), 7.30 (t, *J* = 7.8 Hz, 2H), 5.85 – 5.66 (m, 1H), 5.62 (br, s, 1H), 5.44 (d, *J* = 5.0 Hz, 1H), 5.41 (d, *J* = 1.3 Hz, 1H), 5.39 – 5.28 (m, 2H), 5.24 (dd, *J* = 10.8, 7.0 Hz, 1H), 5.01 (br, s, 1H), 4.77 (dd, *J* = 11.8, 3.3 Hz, 1H), 4.72 – 4.45 (m, 2H), 4.40 (t, *J* = 4.7 Hz, 1H), 3.96 (d, *J* = 12.2 Hz, 1H), 2.80 – 2.67 (m, 2H), 2.33 (s, 1H), 2.23 – 2.13 (m, 1H), 2.07 (m, 1H), 1.91 – 1.80 (m, 1H), 1.77 (s, 3H), 1.76 – 1.70 (m, 1H), 1.66 (m, 6H), 1.28 – 1.20 (m, 1H), 1.11 (s, 3H), 1.05 – 0.90 (m, 9H), 0.88 (dd, *J* = 6.6, 3.8 Hz, 6H), 0.22 (s, 3H), 0.15 (s, 3H) ppm.

^{13}C-NMR (101 MHz, $CDCl_3$): δ = 173.9, 172.4, 166.5, 166.3, 165.9, 165.2, 133.7, 133.6, 133.3, 133.2, 133.1, 130.2, 130.03, 129.99, 129.96, 129.91 129.84, 128.82, 129.3, 129.2, 128.7, 128.5, 128.4, 125.7, 123.5, 106.8, 82.4, 81.5, 78.14, 78,07, 77.4, 75.7*, 73.0*, 71.9*, 71.4*, 64.3, 32.0, 30.5, 29.9, 26.3, 25.8, 25.5, 22.6, 22.6, 18.3, 13.2, 12.3, 1.17, −3.25 ppm.

**Signals were identified by analysis of 2D-NMR (HSQC, HMBC).*

IR (Diamond-ATR, neat): ṽ = 2957 (m), 2360 (w), 1724 (s), 1602 (w), 1584 (w), 1452 (m), 1351 (m), 1268 (s), 1167 (m), 1108 (s), 1069 (m), 1027 (m), 973 (m), 838 (m) cm^{-1}.

HRMS (ESI-TOF) m/z: $[M+H]^+$ calcd. for $C_{61}H_{75}O_{15}Si^+$: 1075.4870; found: 1075.4881.

¹H-NMR (400 MHz, $CDCl_3$):

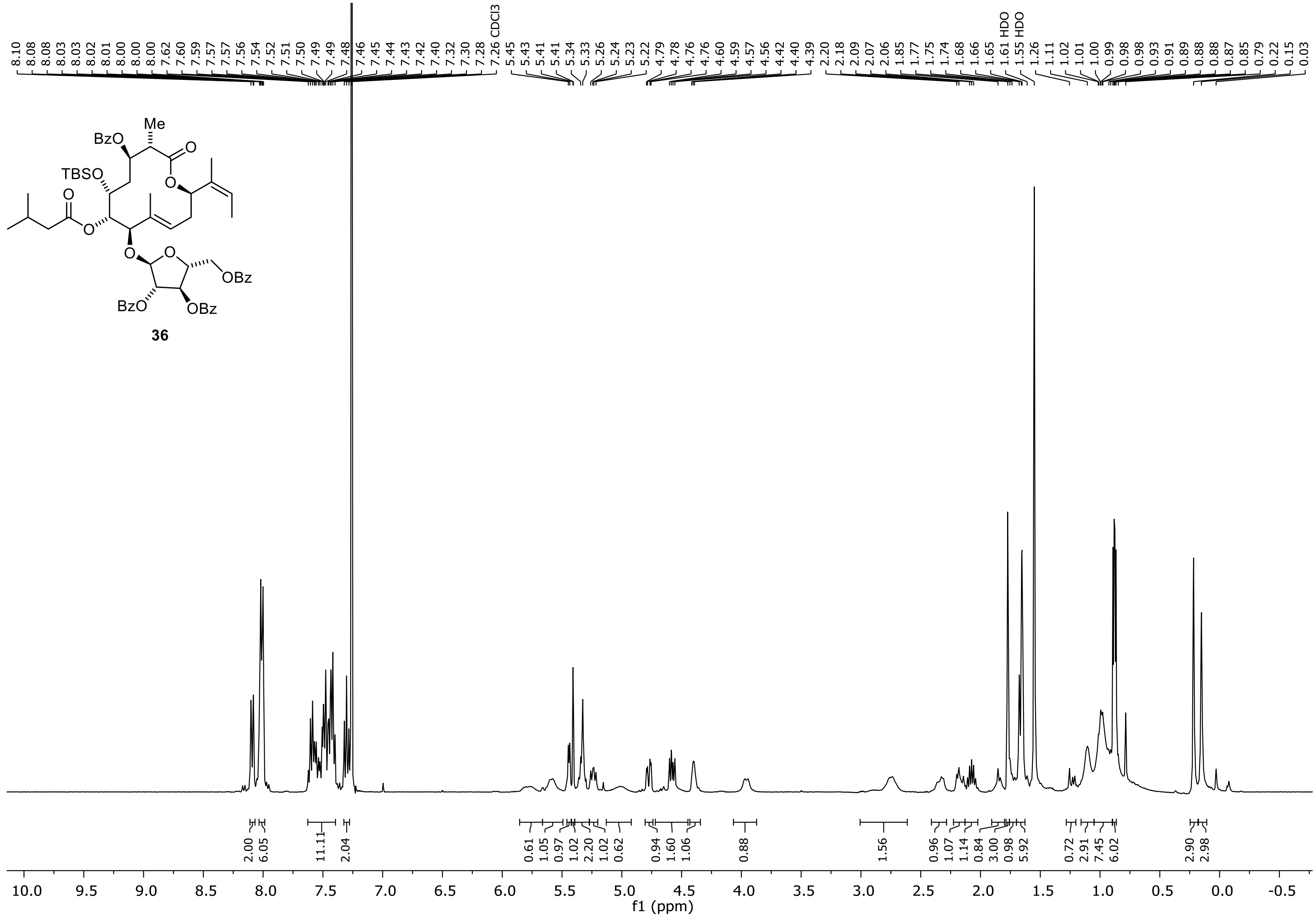

^{13}C-NMR (101 MHz, $CDCl_3$):

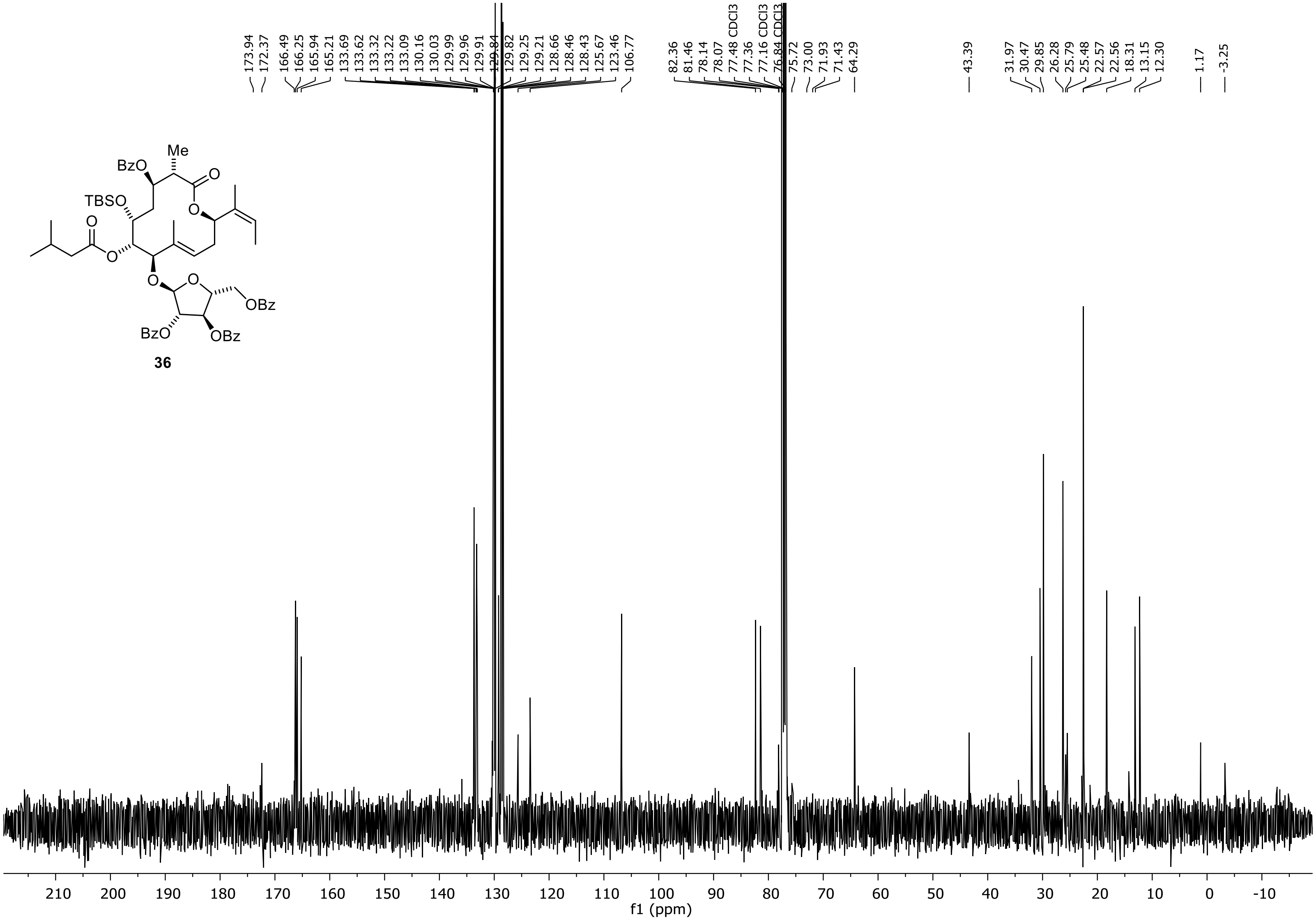

HSQC (400 MHz, $CDCl_3$):

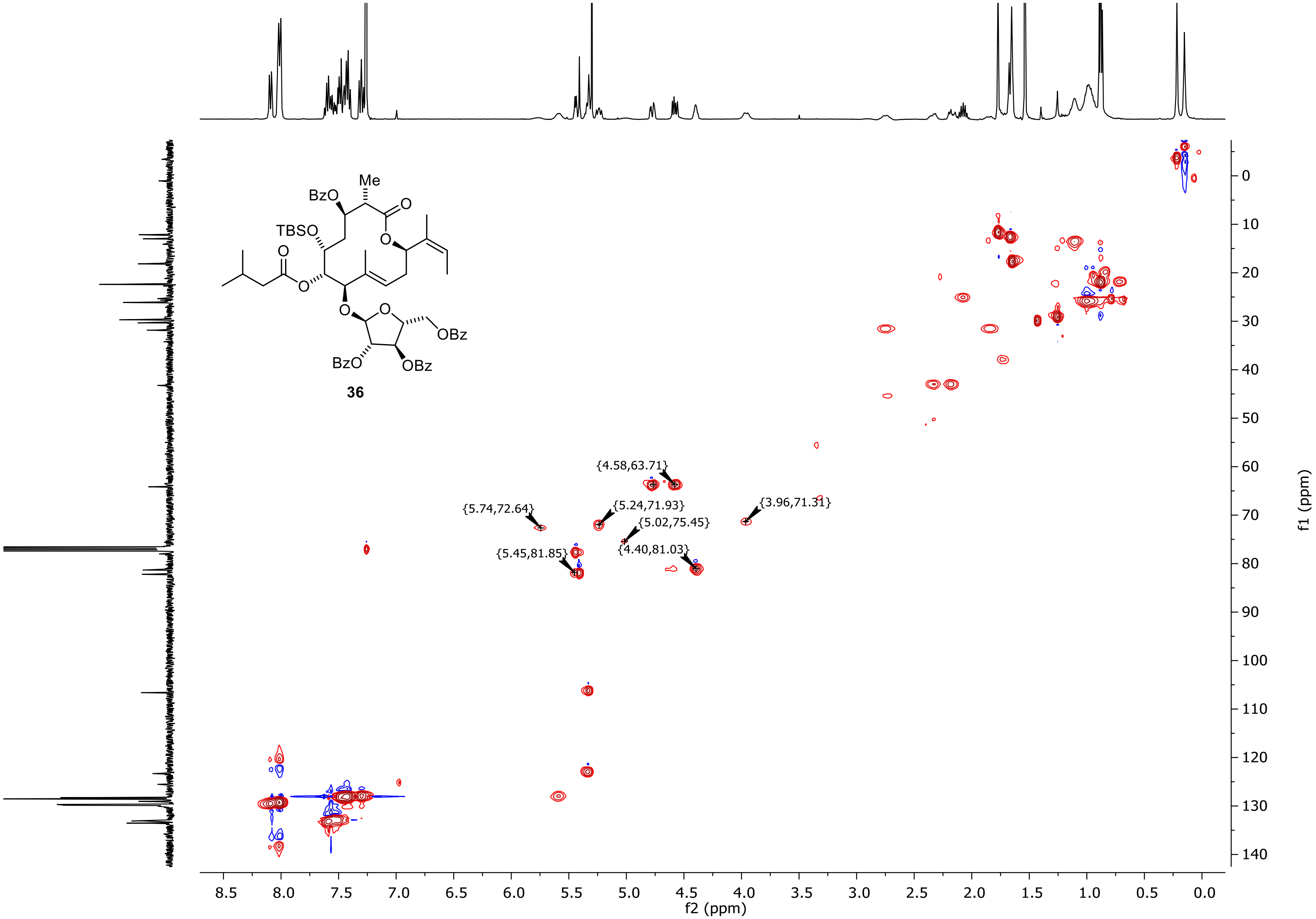

Preparation of 37a/b: Deprotection of TBS ether 36

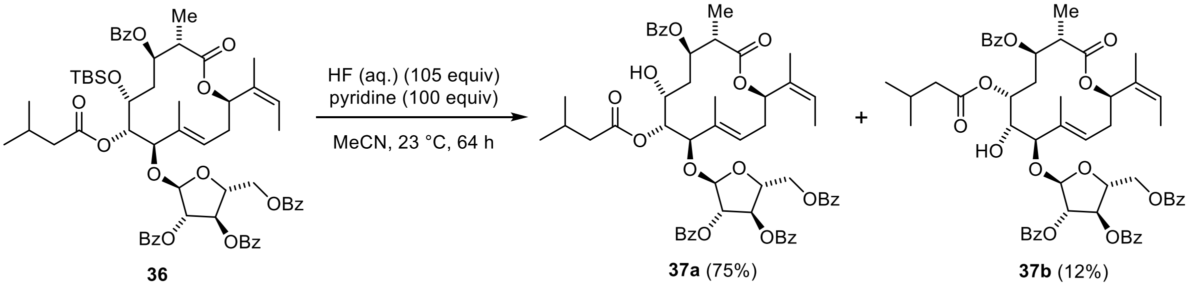

Within a 6 mL Teflon-coated vessel, pyridine (860 μL, 10.7 mmol, 50.0 equiv) was added to a solution of silyl ether **36** (230 mg, 214 μmol, 1.00 equiv) in acetonitrile (1.5 mL). The vessel was secured by clamping and stirring was adjusted to low speed (200 – 300 rpm). Using a plastic pipette, aqueous hydrofluoric acid solution (48%, 460 μL, 10.8 mmol, 50.5 equiv) was added carefully, the reaction vessel was covered loosely with a yellow cap and stirring was continued for 48 h at 23 °C. TLC analysis (NOTE 1) indicated presence of starting material and additional pyridine (860 μL, 10.7 mmol, 50.0 equiv) and aqueous hydrofluoric acid solution (48%, 500 μL, 11.7 mmol, 55.0 equiv) were added. After an additional 16 h of reaction time, TLC analysis (NOTE 1) consumption of most of the starting material, but also the formation of significant amounts of side product **37b**. The reaction was terminated by careful transfer of the mixture into a stirring solution of aqueous saturated sodium bicarbonate (10 mL) using a plastic pipette. Diethyl ether (10 mL) was added, the phases were separated, and the aqueous layer was extracted further with diethyl ether (2 x 10 mL). The combined organic phases were washed with aqueous saturated sodium bicarbonate solution (10 mL), brine (10 mL), were dried over anhydrous sodium sulfate, filtered, and concentrated. Column chromatography over silica gel (10 to 20% EtOAc in hexanes gradient) afforded the acyl migrated side product **37b** (first, less polar fraction, 23.8 mg, 24.8 μmol, 12%) along the desired secondary alcohol **37a** (second, more polar fraction, 155 mg, 161 μmol 75%) as clear viscous oils, respectively.

NOTE 1: A mini workup was executed as follows: One drop of the reaction mixture was removed from the reaction mixture by use of a plastic pipette and was partitioned between aqueous saturated sodium bicarbonate solution (100 μL) and diethyl ether (100 μL), the organic layer was spotted.

Characterization Data for 37a:

R*f* (30% ethyl acetate in hexanes) = 0.42 (UV, CAM).

$[\boldsymbol{\alpha}]_{\mathbf{D}}^{\mathbf{26}}$ = −10.1° (c = 2, $CHCl_3$).

^{1}H-NMR (400 MHz, $CDCl_3$): δ = 8.08 (d, *J* = 7.7 Hz, 2H), 8.04 – 7.96 (m, 6H), 7.62 – 7.37 (m, 10H), 7.31 (t, *J* = 7.7 Hz, 2H), 6.03 (dd, *J* = 11.9, 3.2 Hz, 1H), 5.54 – 5.29 (m, 7H), 4.77 (dd, *J* = 11.9, 3.4 Hz, 1H), 4.60 (dd, *J* = 11.9, 5.7 Hz, 1H), 4.47 – 4.40 (m, 1H), 4.13 – 4.04 (m, 2H), 3.00 (dq, *J* = 13.3, 6.7 Hz, 1H), 2.65 (dt, *J* = 14.6, 11.9 Hz, 1H), 2.56 (d, *J* = 3.7 Hz, 1H), 2.23 (d, *J* = 7.2 Hz, 2H), 2.03 (dt, *J* = 13.4, 7.1 Hz, 1H), 1.95 (d, *J* = 14.6 Hz, 1H), 1.82 – 1.73 (m, 4H), 1.70 (s, 3H), 1.66 (d, *J* = 7.1 Hz, 3H), 1.21 (d, *J* = 6.7 Hz, 3H), 0.82 (d, *J* = 6.6 Hz, 6H) ppm.

^{13}C-NMR (101 MHz, $CDCl_3$): δ = 173.58, 173.56, 166.3, 165.9, 165.6, 165.3, 135.54 133.7, 133.6, 133.4, 133.2, 132.9, 130.1, 129.99, 129.96, 129.90, 129.84, 129.80, 129.7, 129.15, 129.14, 128.69, 128,66, 128.4, 127.4, 123.7, 106.6, 84.6, 82.4, 81.6, 77.9, 77.4, 74.3 71.0, 67.8, 64.1, 43.6, 42.1, 31.9, 31.5, 25.8, 22.42, 22.39, 18.4, 15.5, 13.3, 11.7 ppm.

IR (Diamond-ATR, neat): ṽ = 3515 (br, w), 2958 (m), 2361 (w), 1724 (s), 1602 (w), 1452 (m), 1315 (m), 1269 (s), 1175 (m), 1111 (s), 1070 (m) 1027 (m), 877 (w) cm^{-1}.

HRMS (APCI-TOF) m/z: $[M+H]^+$ calcd. for $C_{55}H_{61}O_{15}^+$: 961.4005; found: 961.4008.

¹H-NMR (400 MHz, $CDCl_3$):

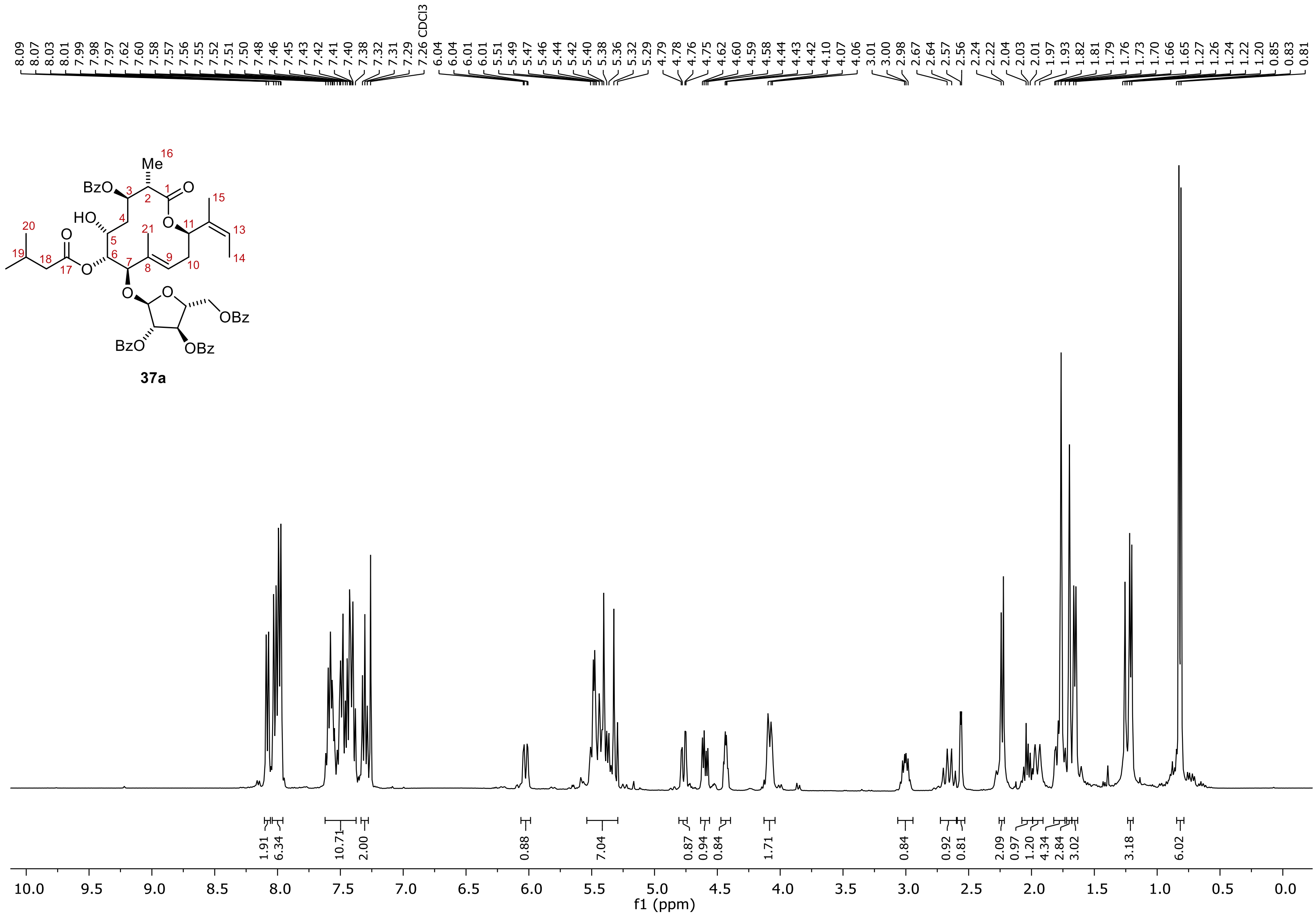

^{13}C-NMR (101 MHz, $CDCl_3$):

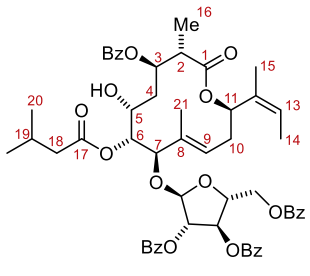

37a

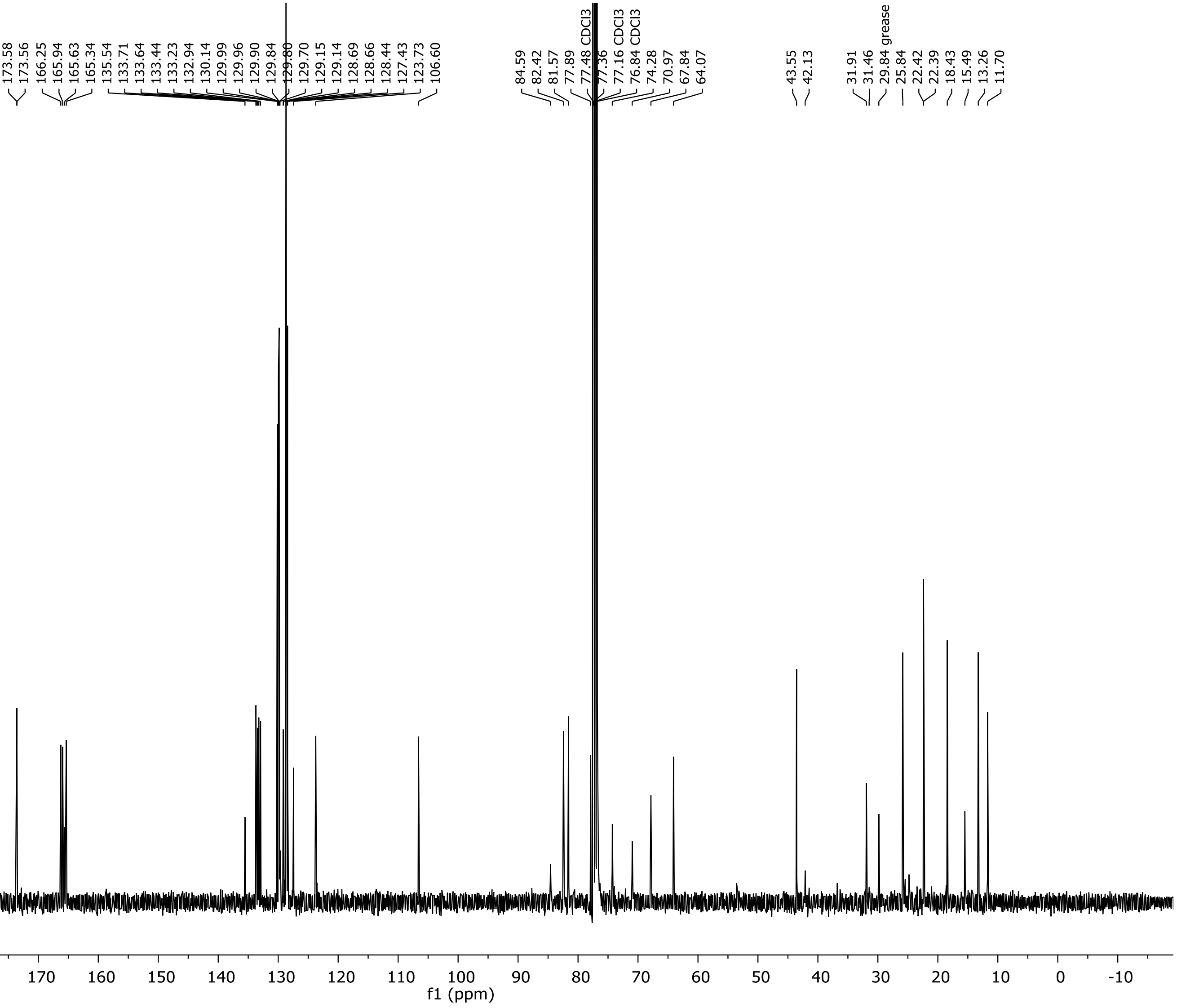

COSY (400 MHz, $CDCl_3$):

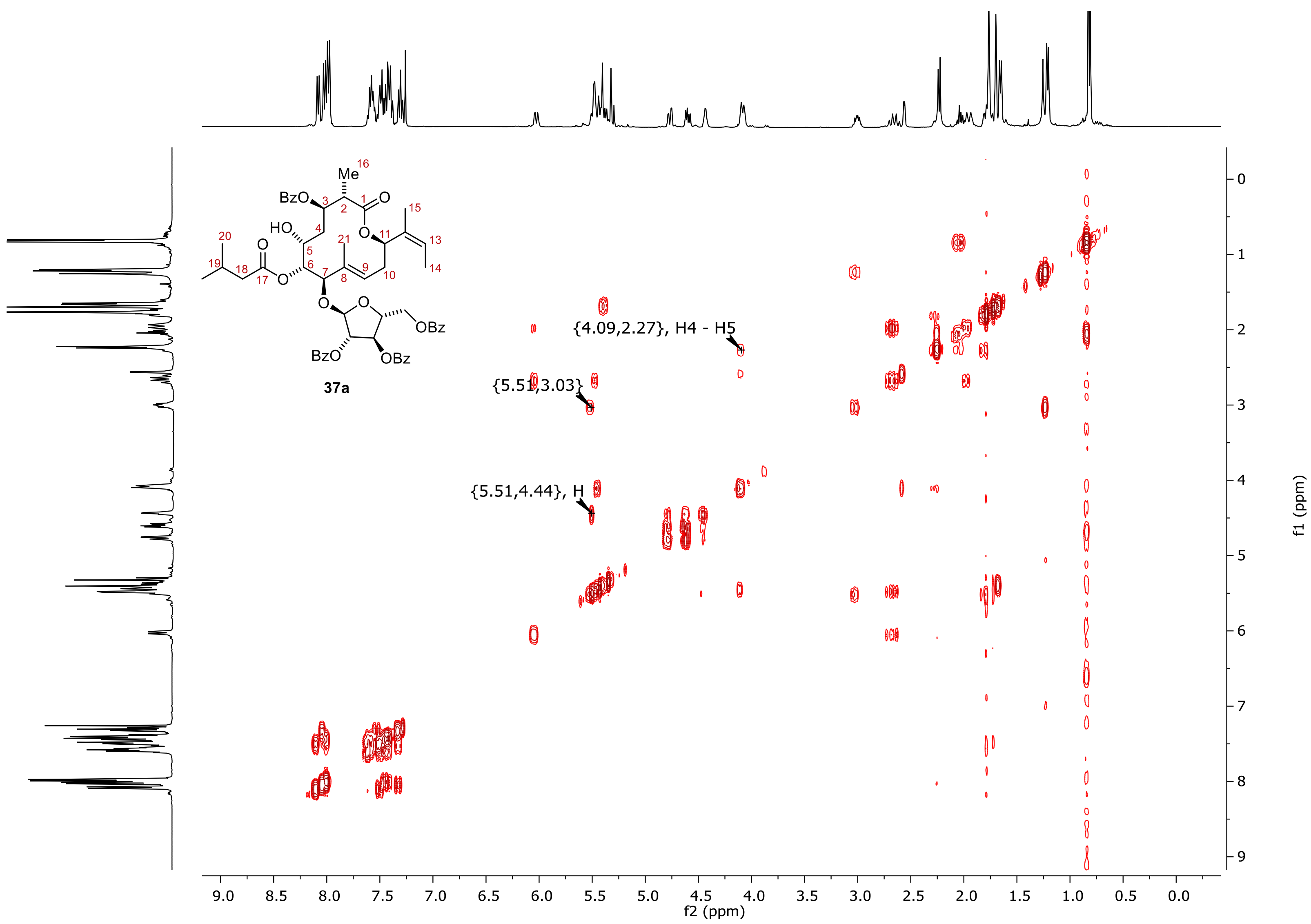

HSQC (400 MHz, CDCl3):

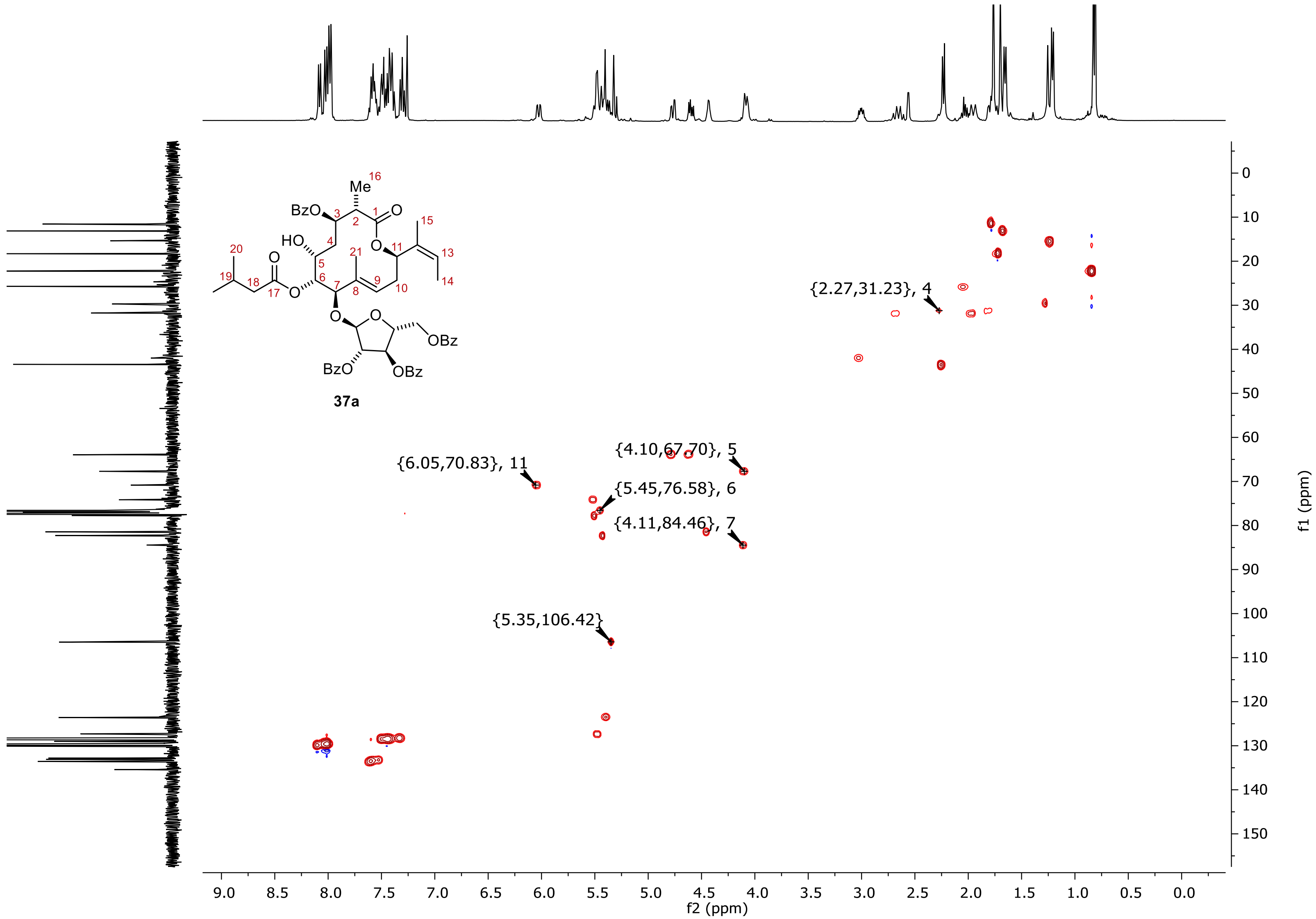

HMBC (400 MHz, $CDCl_3$):

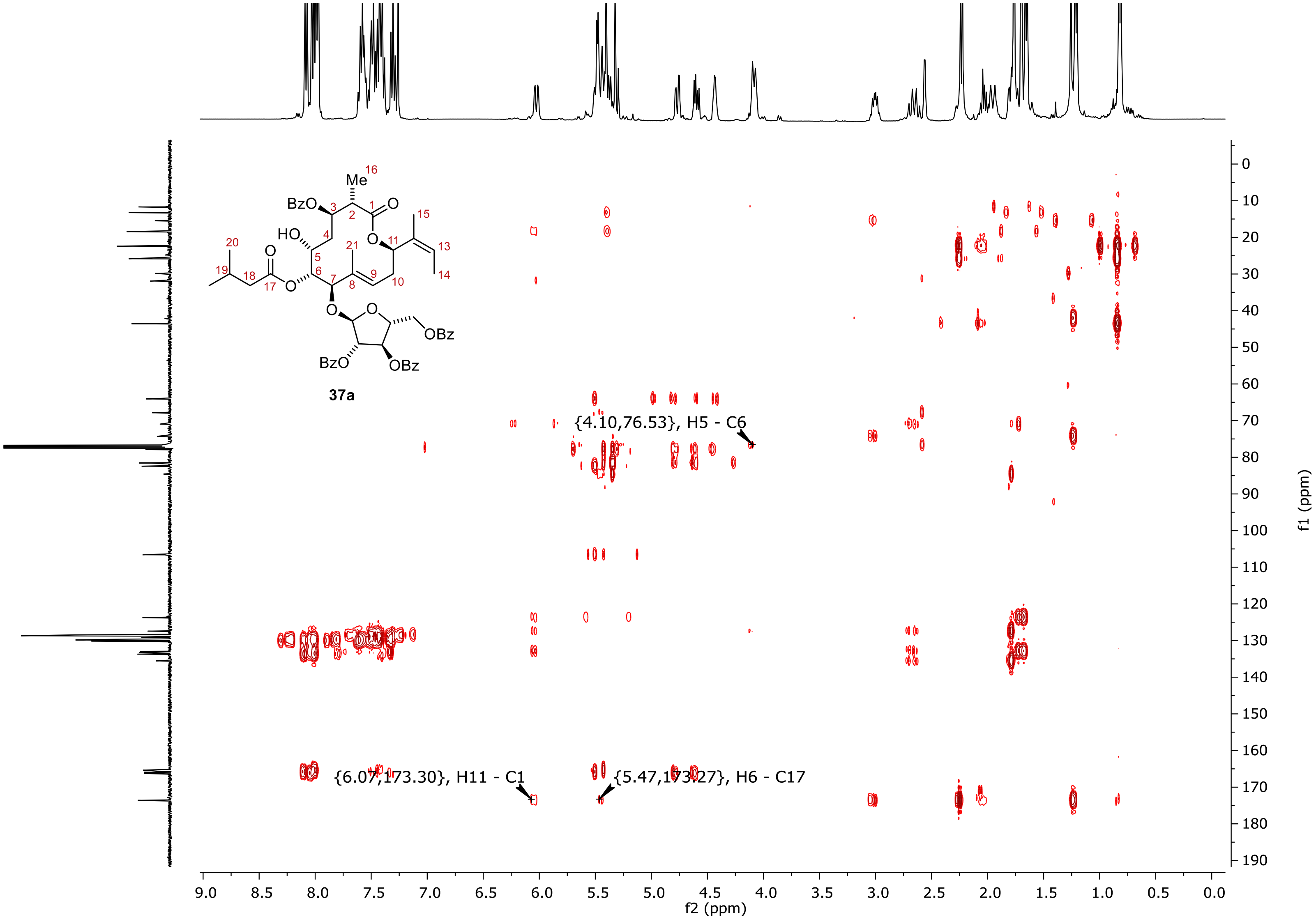

Characterization Data for 37b:

R$_f$ (30% EtOAc in hexanes) = 0.45 (UV, CAM).

$[\boldsymbol{\alpha}]_{\mathbf{D}}^{\mathbf{22}}$ = −6.2° (c = 2, $CHCl_3$).

^{1}H-NMR (400 MHz, $CDCl_3$): δ = 8.07 – 7.95 (m, 8H), 7.61 – 7.48 (m, 4H), 7.48 – 7.39 (m, 6H), 7.33 (t, *J* = 7.7 Hz, 2H), 6.08 (dd, *J* = 11.9, 3.2 Hz, 1H), 5.57 (dt, *J* = 8.1, 4.1 Hz, 1H), 5.48 (d, *J* = 2.2 Hz, 1H), 5.44 (d, *J* = 11.2 Hz, 1H), 5.41 – 5.29 (m, 3H), 5.24 (d, *J* = 11.8 Hz, 1H), 4.74 (dd, *J* = 11.9, 3.4 Hz, 1H), 4.61 (dd, *J* = 11.8, 5.8 Hz, 1H), 4.53 (td, *J* = 5.8, 3.4 Hz, 1H), 4.00 (dd, *J* = 8.6, 1.9 Hz, 1H, *H6*), 3.86 (d, *J* = 8.6 Hz, 1H, *z*), 2.99 (dq, *J* = 13.5, 6.8 Hz, 1H), 2.79 – 2.57 (m, 3H), 1.98 – 1.87 (m, 3H), 1.79 (s, 3H), 1.73 (d, *J* = 3.2 Hz, 3H), 1.70 (d, *J* = 3.5 Hz, 1H), 1.65 (dd, *J* = 6.9, 1.8 Hz, 3H), 1.22 (d, *J* = 6.8 Hz, 3H), 0.83 (dd, *J* = 8.3, 6.2 Hz, 6H) ppm.

^{13}C-NMR (101 MHz, $CDCl_3$): δ = 173.8, 172.8, 166.2, 166.1, 165.8, 165.7, 135.1, 133.82, 133.76, 133.3, 133.2, 133.1, 130.5, 130.1, 129.99, 129.95, 129.9, 129.7, 129.1, 129.0, 128.7, 128.5, 128.4, 127.6, 123.6, 107.0, 88.2, 83.3, 80.6, 77.4, 74.7, 74.0, 70.4, 69.1, 64.1, 43.4, 41.5, 32.1, 27.2, 25.4, 22.43, 22.42, 18.6, 16.1, 13.3, 11.5 ppm.

**One aromatic signal is missing due to overlap of two diastereotopic signals.*

IR (Diamond-ATR, neat): ṽ = 3527 (br, w), 2959 (m), 1720 (s), 1602 (w), 1451 (m), 1375 (m), 1266 (s), 1174 (m), 1109 (s), 1069 (m), 1027 (m), 977 (m), 879 (w) cm^{-1}.

HRMS (APCI-TOF) m/z: $[M+H]^+$ calcd. for $C_{55}H_{61}O_{15}^+$: 961.4005; found: 961.4038.

¹H-NMR (400 MHz, CDCl₃):

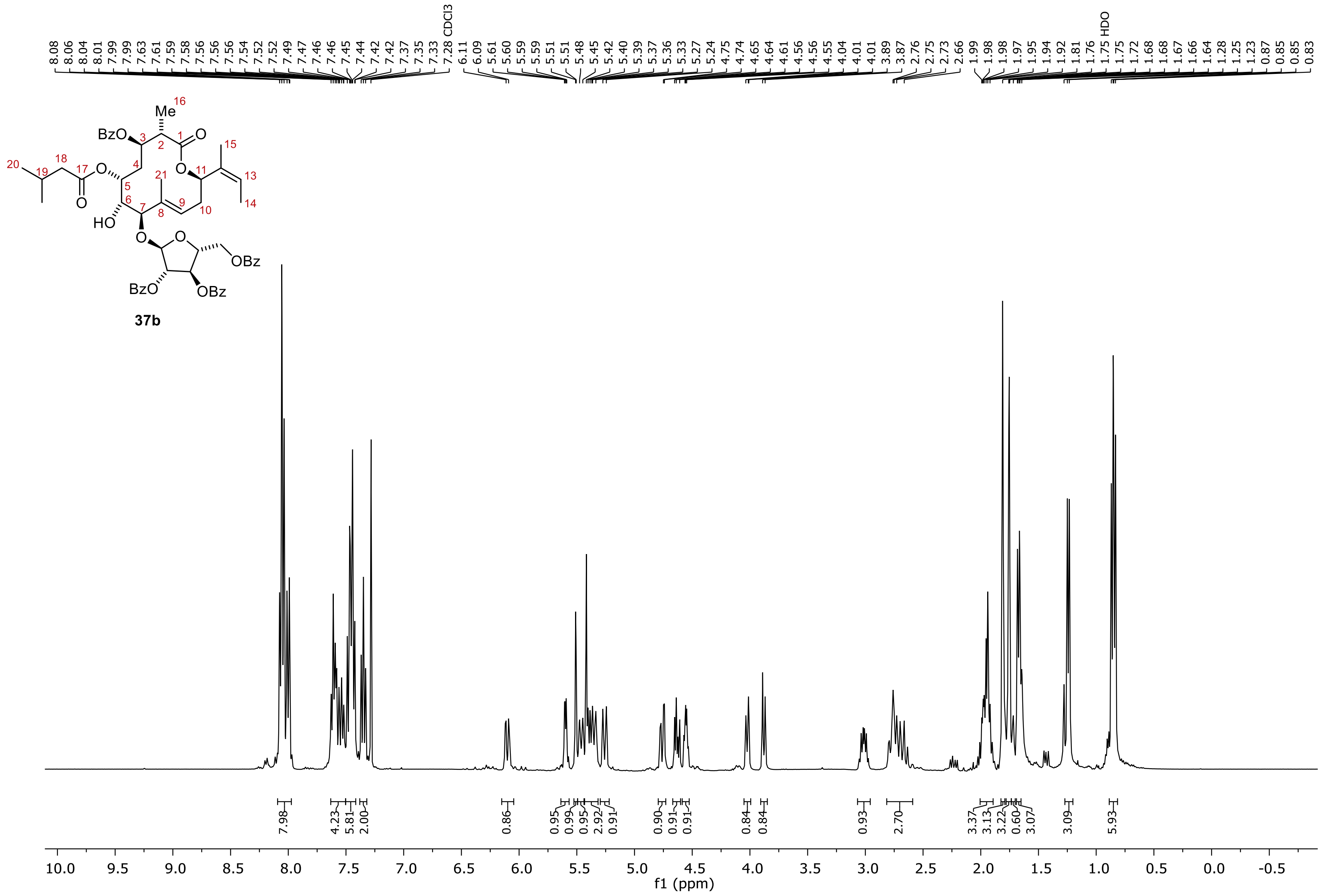

^{13}C-NMR (101 MHz, $CDCl_3$):

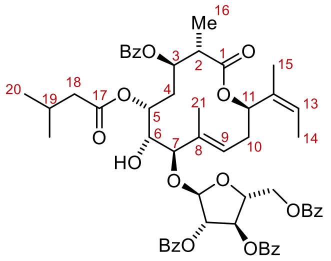

37b

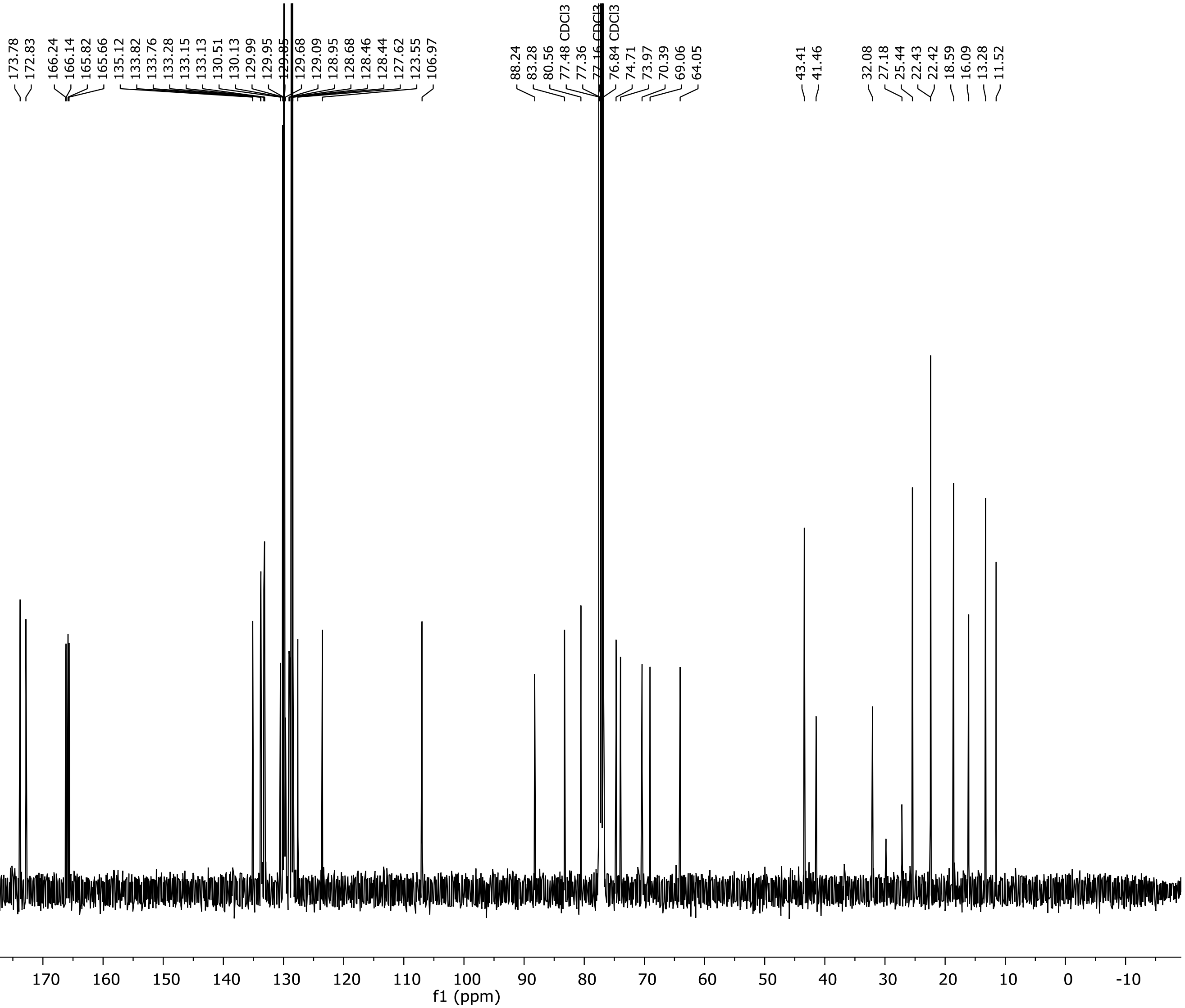

COSY (400 MHz, CDCl3):

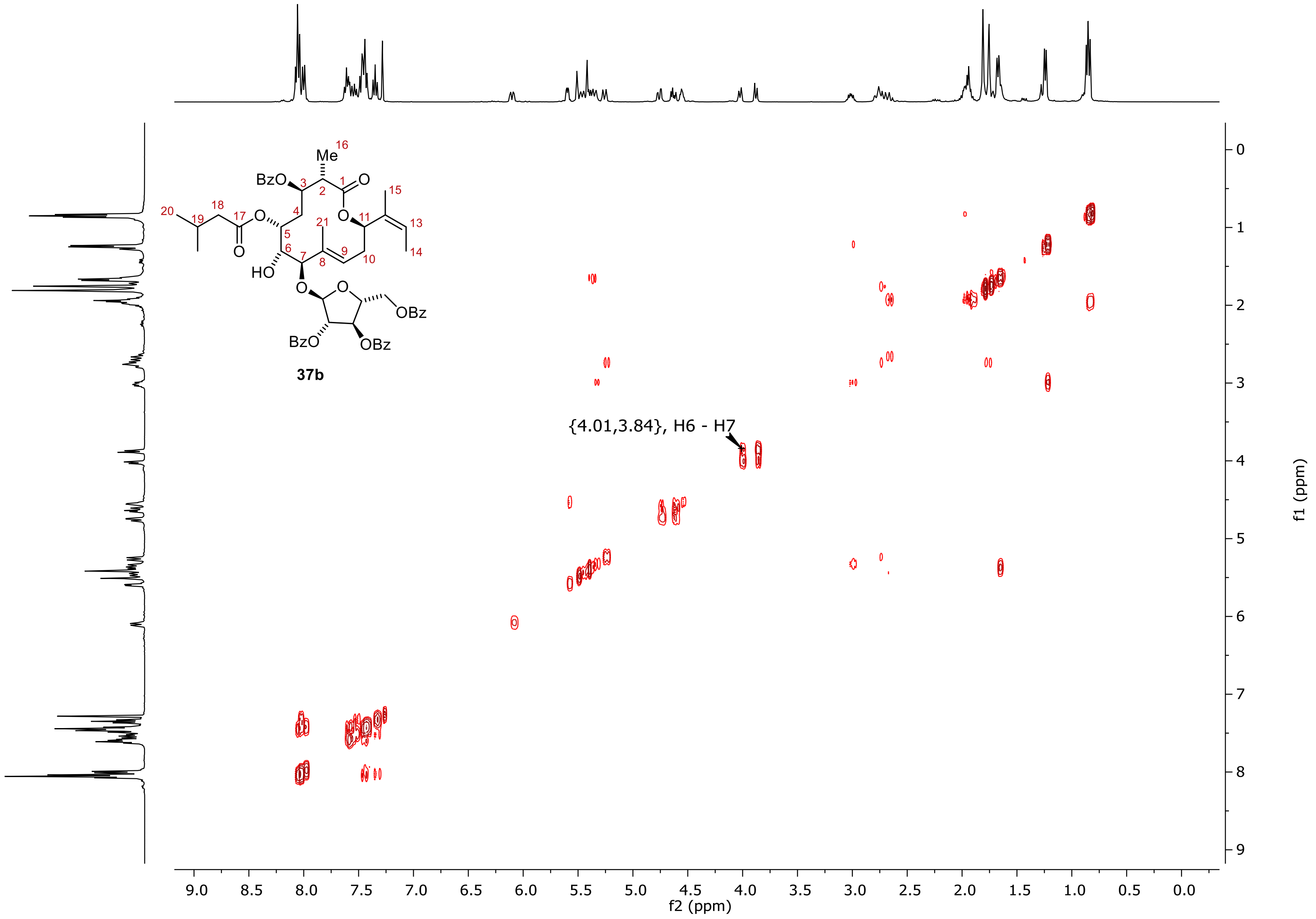

HSQC (400 MHz, $CDCl_3$):

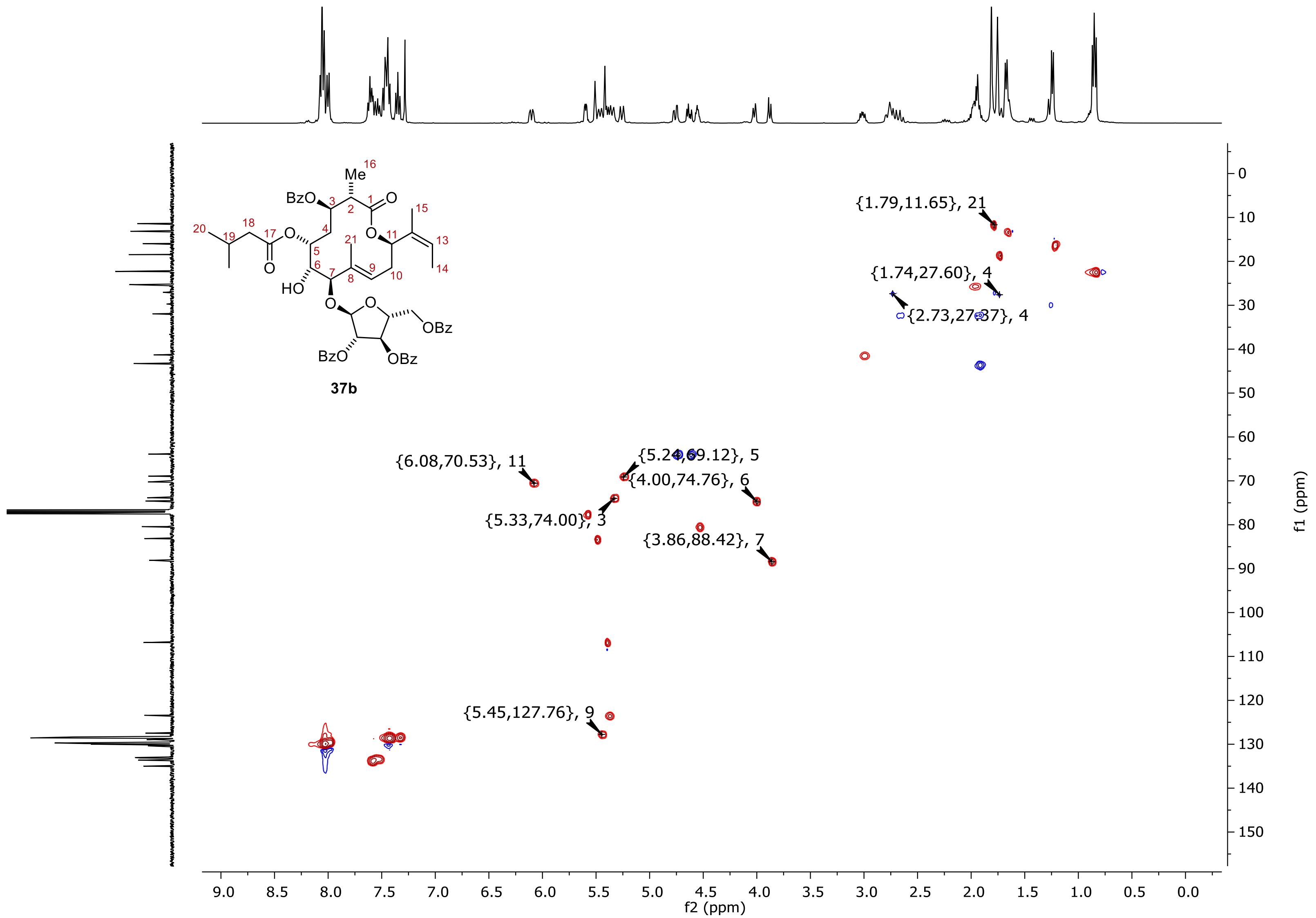

HMBC (400 MHz, $CDCl_3$):

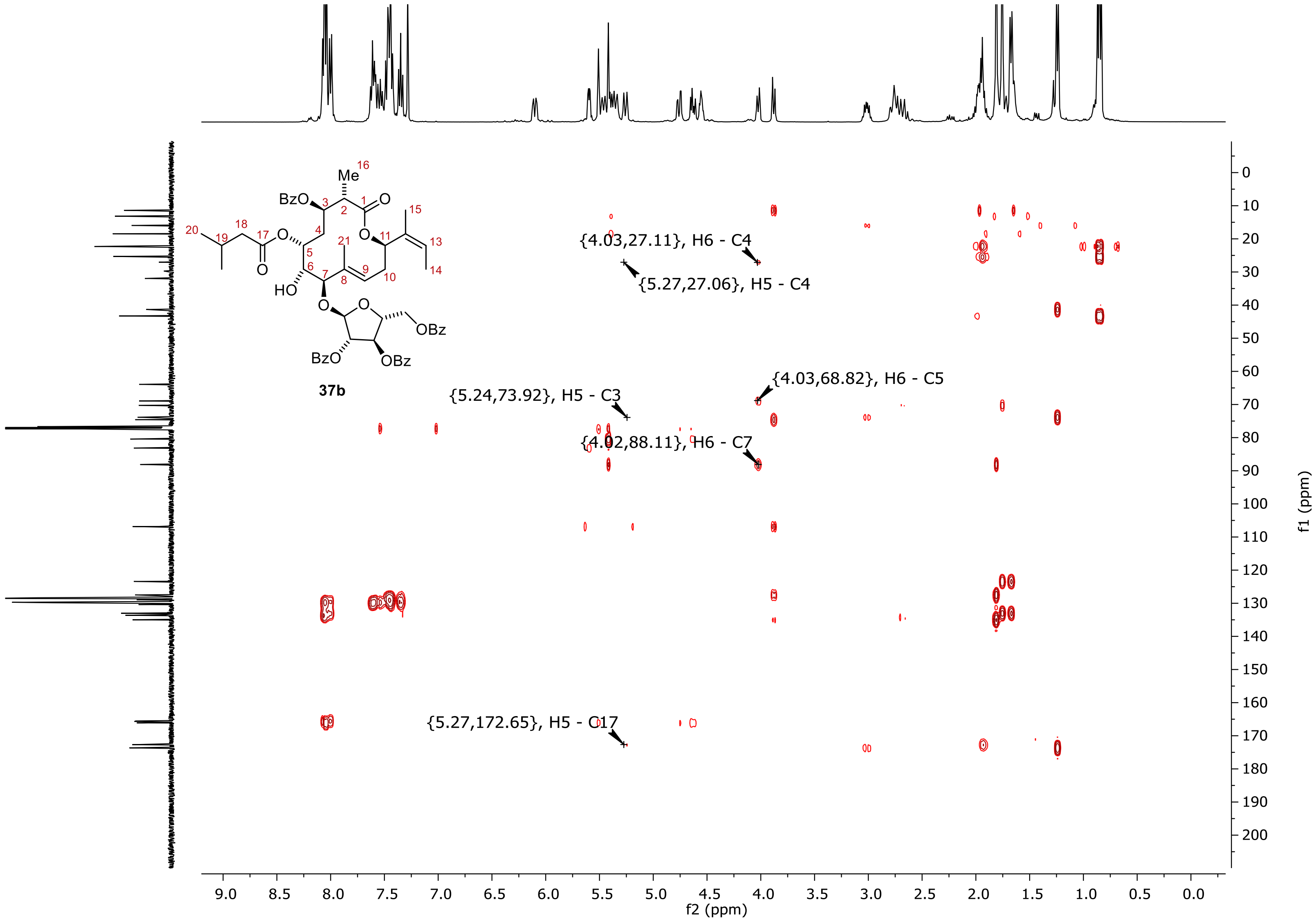

Preparation of 38: DMP Oxidation of Secondary Alcohol 37a

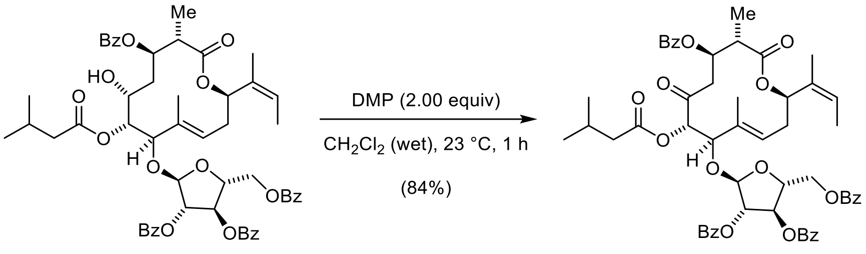

37a **38**

The biphasic mixture of dichloromethane (3 mL) and water (3 mL) was mixed vigorously in a separatory funnel. After separation of the phases, a solution of secondary alcohol **37a** (29.9 mg, 31.1 µmol, 1.00 equiv) in the water saturated dichloromethane (500 µL) was prepared and DMP (26.4 mg, 62.2 µmol, 2.00 equiv) was added in one portion at room temperature (23 °C). After 1 h of stirring TLC analysis (NOTE 1) indicated complete consumption of starting material. Aqueous saturated sodium bicarbonate solution (500 µL) was added, the biphaseic mixture was stirred vigorously for 15 min before aqueous saturated sodium bisulfate solution (500 µL) was added and stirring was continued for another 30 min. The phases were separated and the aqueous layer was extracted with diethyl ether (2 x 1 mL). The combined organic phases were washed with bine (2 mL), dried over anhydrous sodium sulfate, filtered, and concentrated. Analysis of the crude NMR indicated a very clean conversion towards ketone **38**. A short silica plug inside a Pasteur pipette (30 to 50% EtOAc in hexanes, NOTE 2) was sufficient in separating residual DMP-impurities and the fourfold benzoylated gulmirecin precursor **38** (25 mg, 26.1 µmol. 84%) was obtained as a white solid.

NOTE 1: A mini workup was prepared as follows: One drop of the reaction mixture was partitioned between aqueous saturated sodium bicarbonate solution (100 µL) and diethyl ether (100 µL), the organic layer was spotted.

NOTE 2: Exposure of the elimination-prone β-keto benzoate **38** to larger amounts of silica or HPLC-purification conditions resulted in elimination towards a corresponding threefold benzoylated disciformycin precursor **39**.

Characterization Data for 38:

R$_f$ (30% EtOAc in hexanes) = 0.60 (UV, CAM).

$[\alpha]_D^{26}$ = +39.0° (c = 2, $CHCl_3$).

^{1}H-NMR (400 MHz, $CDCl_3$): δ = 8.07 – 7.98 (m, 8H), 7.63 – 7.38 (m, 10H), 7.32 (t, *J* = 7.8 Hz, 2H), 5.86 (dd, *J* = 11.9, 2.7 Hz, 1H), 5.54 – 5.43 (m, 4H), 5.36 (td, *J* = 7.5, 4.2 Hz, 2H), 5.09 (d, *J* = 9.1 Hz, 1H), 4.79 (dd, *J* = 11.9, 3.5 Hz, 1H), 4.62 (dd, *J* = 11.9, 5.7 Hz, 1H), 4.46 (td, *J* = 5.3, 3.6 Hz, 1H), 4.36 (d, *J* = 9.0 Hz, 1H), 3.37 (td, *J* = 11.3, 6.0 Hz, 1H), 3.18 (dd, *J* = 20.2, 4.6 Hz, 1H), 2.95 (dd, *J* = 20.2, 2.7 Hz, 1H), 2.62 (dt, *J* = 14.5, 11.5 Hz, 1H), 2.20 (dd, *J* = 7.2, 2.6 Hz, 2H), 1.99 (dq, *J* = 13.6, 6.8 Hz, 1H), 1.93 – 1.86 (m, 1H), 1.77 (d, *J* = 1.4 Hz, 3H), 1.69 – 1.66 (m, 3H), 1.65 (dd, *J* = 7.0, 1.8 Hz, 3H), 1.18 (d, *J* = 6.9 Hz, 3H), 0.82 (dd, *J* = 6.7, 2.6 Hz, 6H) ppm.

^{13}C-NMR (101 MHz, $CDCl_3$): δ = 200.3, 173.3, 172.4, 166.1, 165.8, 135.7, 165.3, 133.9, 133.7, 133.6, 133.5, 133.2, 133.1, 133.0, 129.9, 129.8, 129.7, 129.6, 129.00, 128.95, 128.6, 128.5, 128.43, 128.36, 128.34, 125.5, 123.5, 106.5, 83.7, 82.1, 81.6, 77.8, 73.0, 70.9, 70.7, 63.8, 42.7, 31.8, 30.3, 29.7, 25.6, 22.21, 22.20, 18.3, 15.5, 13.1, 11.5 ppm.

IR (Diamond-ATR, neat): ṽ = 2920 (m), 2850 (w), 2360 (w), 2341 (w), 1724 (s), 1602 (w), 1585 (w), 1452 (m), 1376 (m), 1315 (m), 1268 (s), 1176 (m), 1109 (s), 1070 (m), 1027 (m), 978 (w), 711 (m) cm^{-1}.

HRMS (APCI-TOF) m/z: $[M+NH_4]^+$ calcd. for $C_{55}H_{62}NO_{15}^+$: 976.4114; found: 976.4118.

^{1}H-NMR (400 MHz, $CDCl_3$):

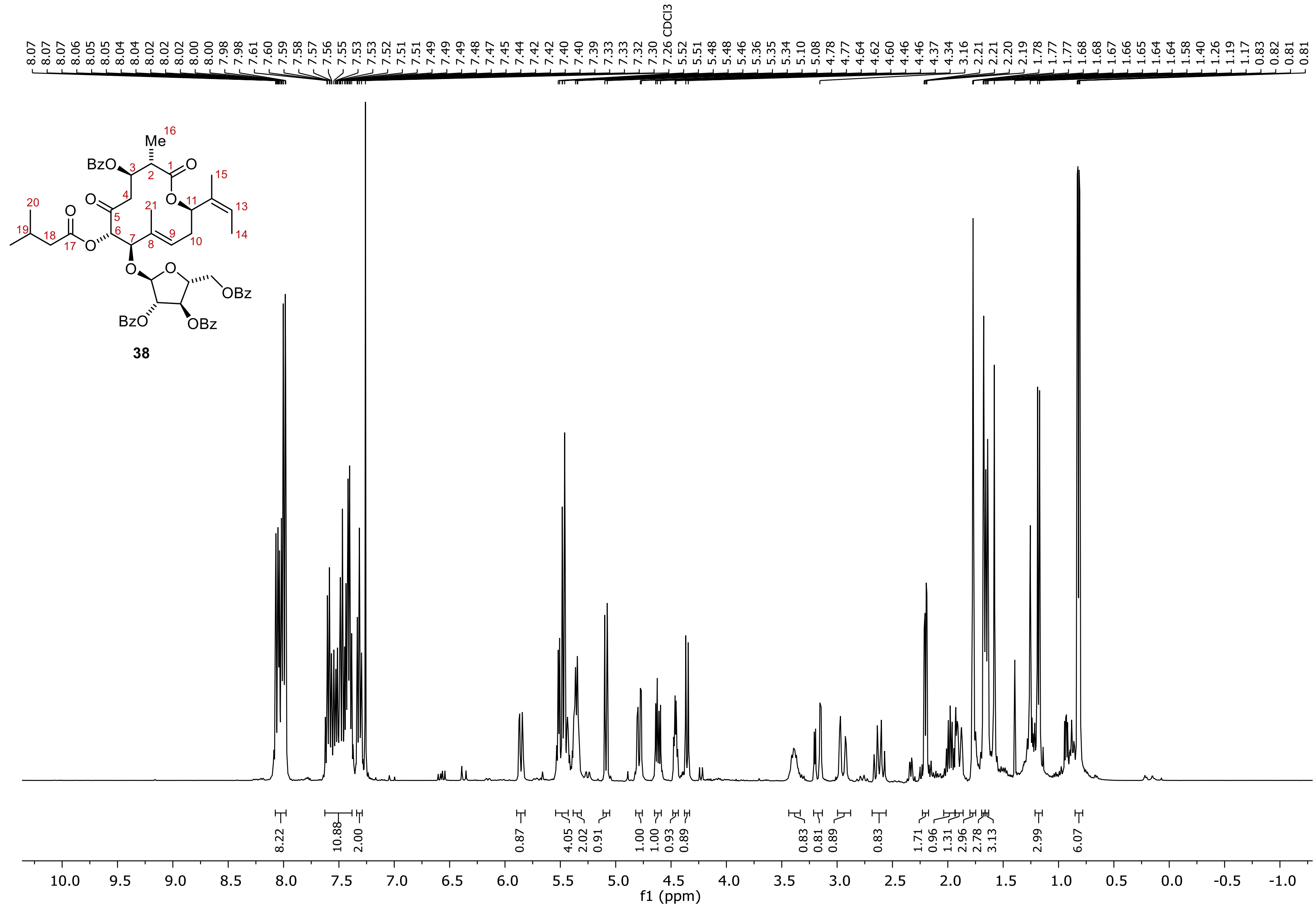

13C-NMR (101 MHz, $CDCl_3$):

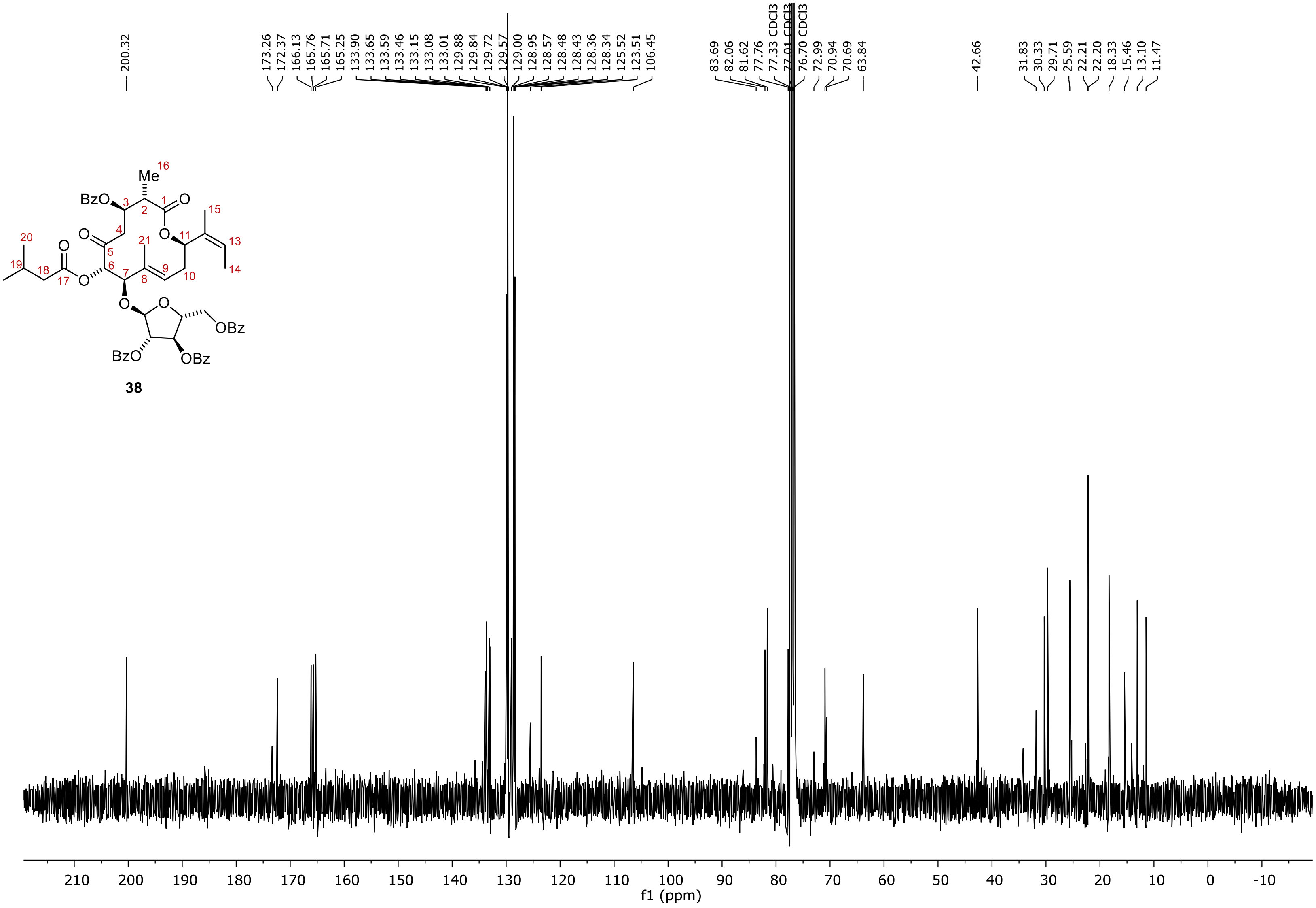

COSY (400 MHz, $CDCl_3$):

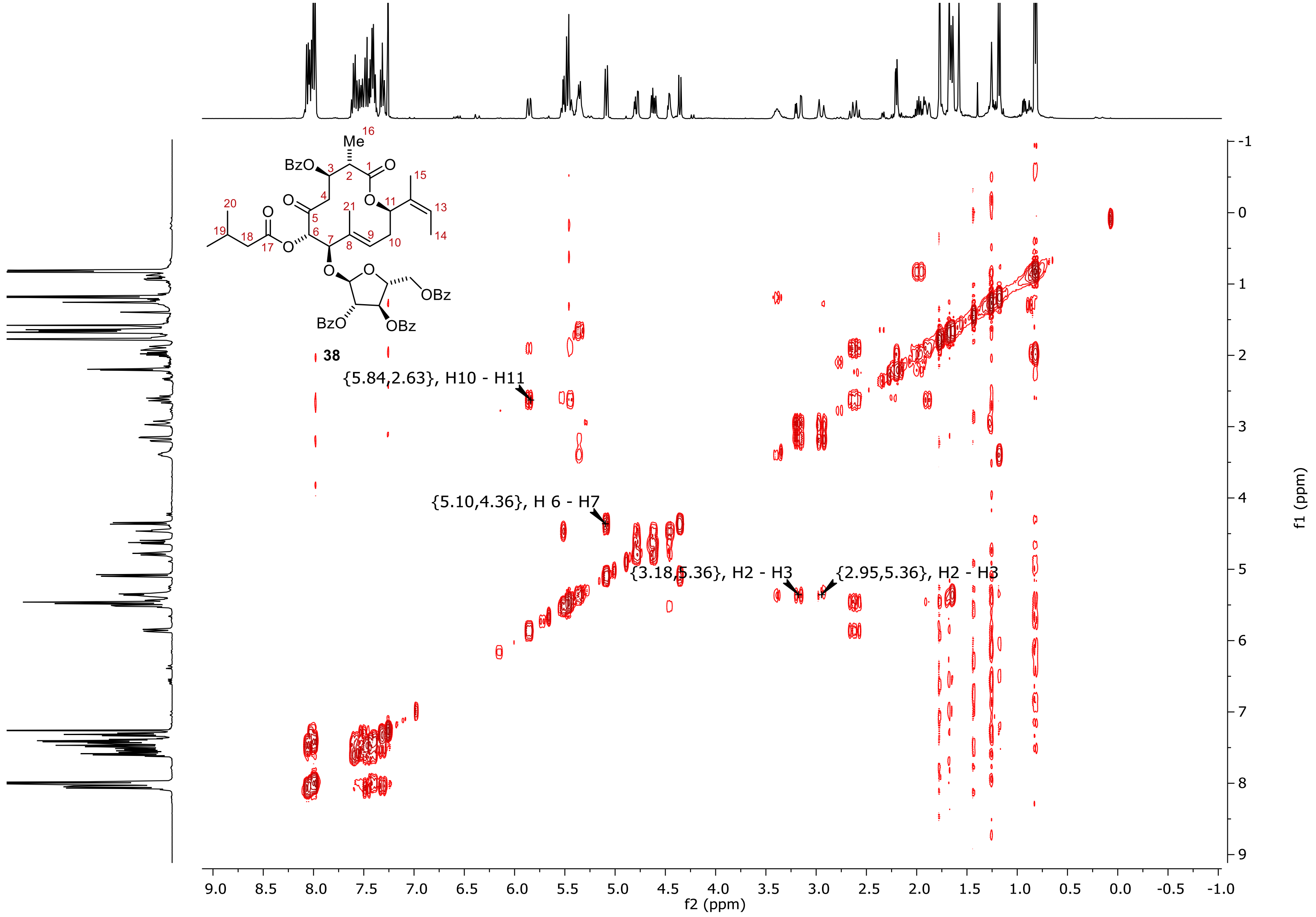

HSQC (400 MHz, $CDCl_3$):

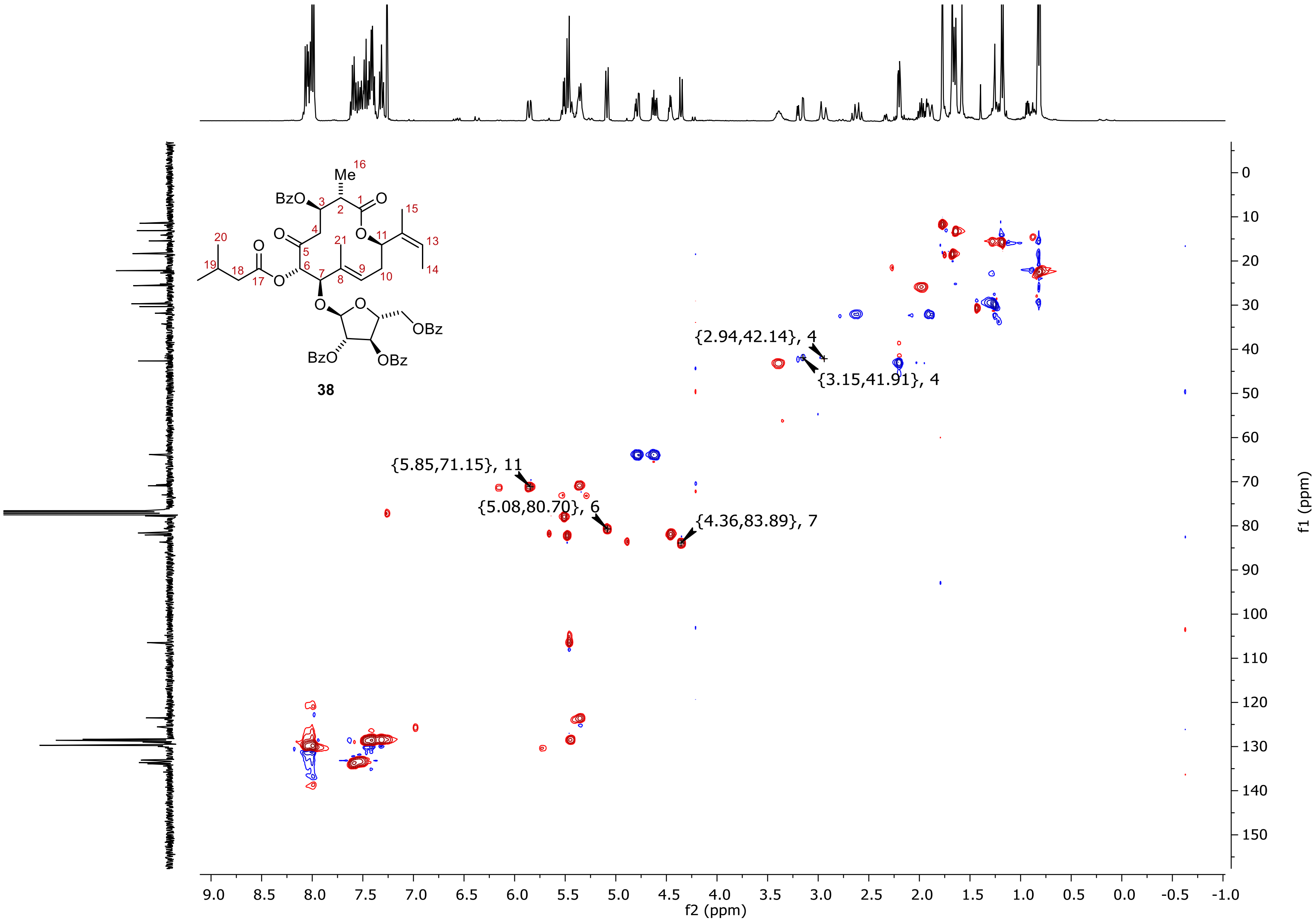

HMBC (400 MHz, $CDCl_3$):

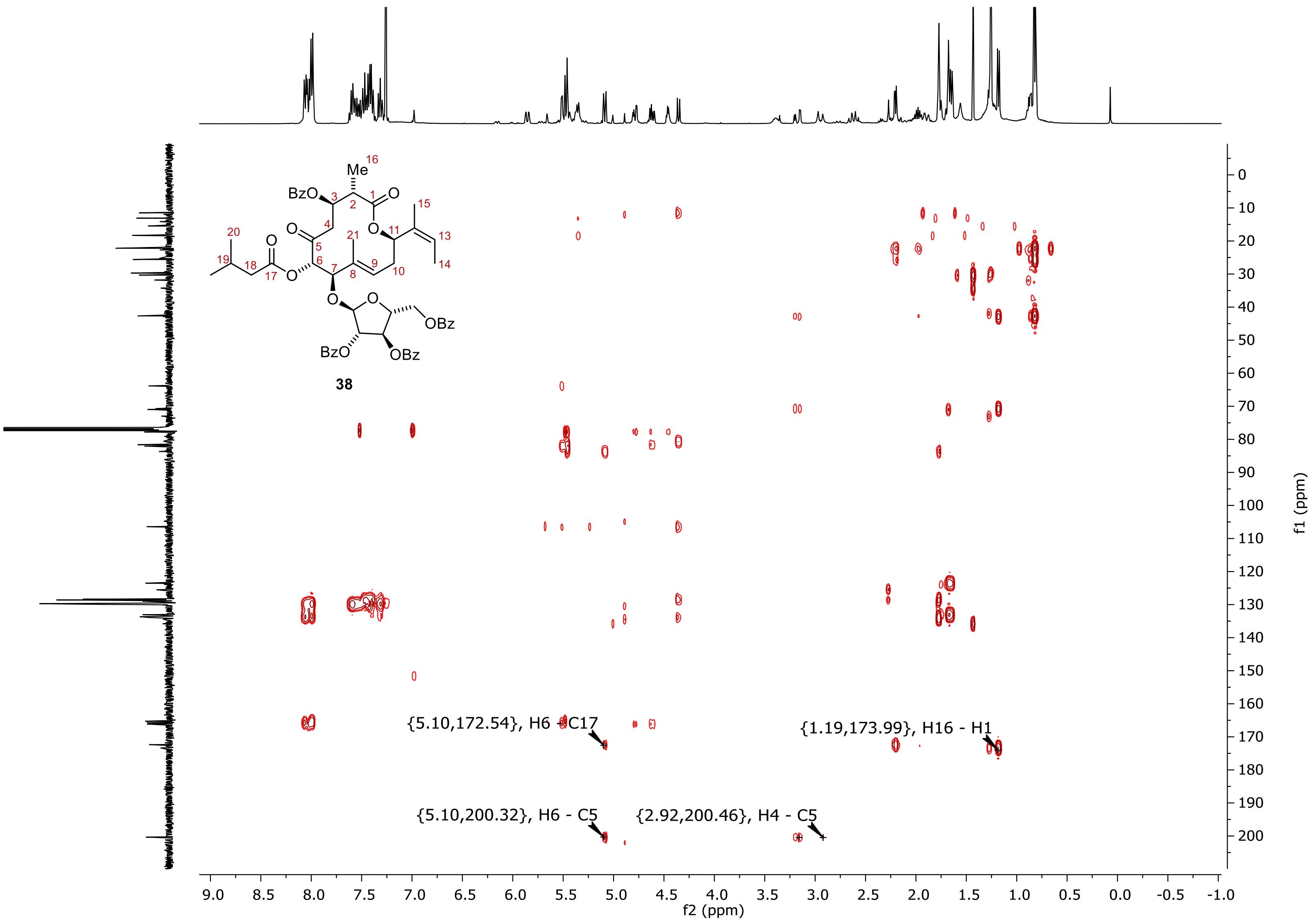

Preparation of 39: Elimination of Benzoate 38 during HPLC-Purification

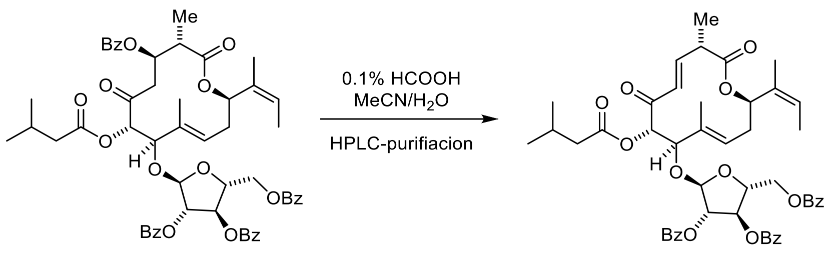

38 39

In an attempt to purify β-keto benzoate **38** (5.0 mg, 5.2 µmol) by HPLC purification, pure enone **39** (2.8 mg, 3.3 µmol, 64%) was obtained as the major compound as a white film. Purification conditions: semi-preparative reverse phase column, 80 to 100% MeCN in H_2O containing 0.1% formic acid each over 8 min gradual elution, 8 mL/min flow rate, detection, and collection at 254 nm.

Characterization Data for 39:

HPLC (Phenomenex, Gemini 5 µm C18, 110 Å, 150 x 10 mm, MeCN/H_2O = 80/20 to MeCN/H_2O = 100/0 over 8 min, flow rate = 8.0 mL/min, I = 254 nm) t_R = 6.44 min.

$[\alpha]_D^{26}$ = +31.2° (c = 0.45, $CHCl_3$).

^{1}H-NMR (400 MHz, $CDCl_3$): δ = 8.10 – 8.06 (m, 2H), 8.00 (td, *J* = 8.4, 1.4 Hz, 4H), 7.65 – 7.56 (m, 2H), 7.50 (dt, *J* = 9.3, 7.6 Hz, 3H), 7.40 (t, *J* = 7.8 Hz, 2H), 7.30 (t, *J* = 7.8 Hz, 2H), 6.57 (dd, *J* = 15.3, 9.1 Hz, 1H), 6.37 (dd, *J* = 15.3, 1.0 Hz, 1H), 5.54 – 5.52 (m, 2H), 5.51 – 5.48 (m, 1H), 5.40 (d, *J* = 10.2 Hz, 1H), 5.38 – 5.32 (m, 2H), 5.25 (dd, *J* = 11.7, 2.8 Hz, 1H), 4.80 (dd, *J* = 11.9, 3.4 Hz, 1H), 4.60 (dd, *J* = 11.9, 5.7 Hz, 1H), 4.39 (td, *J* = 5.3, 3.5 Hz, 1H), 4.23 (d, *J* = 10.2 Hz, 1H), 3.36 – 3.28 (m, 1H), 2.77 (dt, *J* = 14.6, 11.4 Hz, 1H), 2.33 (dd, *J* = 7.2, 1.8 Hz, 2H), 2.11 (dp, *J* = 13.5, 6.7 Hz, 1H), 1.93 (d, *J* = 2.5 Hz, 3H), 1.88 (d, *J* = 3.9 Hz, 1H), 1.67 – 1.63 (m, 6H), 1.24 (s, 3H), 0.93 (dd, *J* = 6.6, 4.0 Hz, 6H) ppm.

^{13}C-NMR (101 MHz, $CDCl_3$): δ = 192.5, 172.7, 171.7, 166.3, 165.9, 165.3, 145.7, 134.3, 133.8, 133.7, 133.3, 133.2, 130.1, 130.03, 130.97, 129.85, 129.83, 129.2, 129.1, 128.7, 128.5, 128.1, 123.4, 106.7, 82.1, 81.7, 81.4, 78.7, 78.2, 72.9, 64.0, 43.1, 43.0, 32.1, 25.9, 22.49, 22.45, 18.2, 14.2, 13.2, 12.6 ppm.

**One aromatic signal is missing due to overlap of two diastereotopic signals.*

IR (Diamond-ATR, neat): ṽ = 2925 (m), 2361 (m), 2340 (m), 1725 (s),1627 (w), 1602 (w), 1452 (w), 1378 (w), 1315 (w), 1267 (s), 1177 (m), 1110 (m), 1070 (m), 1028 (m), 970 (w) cm^{-1}.

HRMS (APCI-TOF) m/z: $[M+H]^+$ calcd. for $C_{48}H_{53}O_{13}^+$: 837.3481; found: 837.3483.

¹H-NMR (400 MHz, $CDCl_3$):

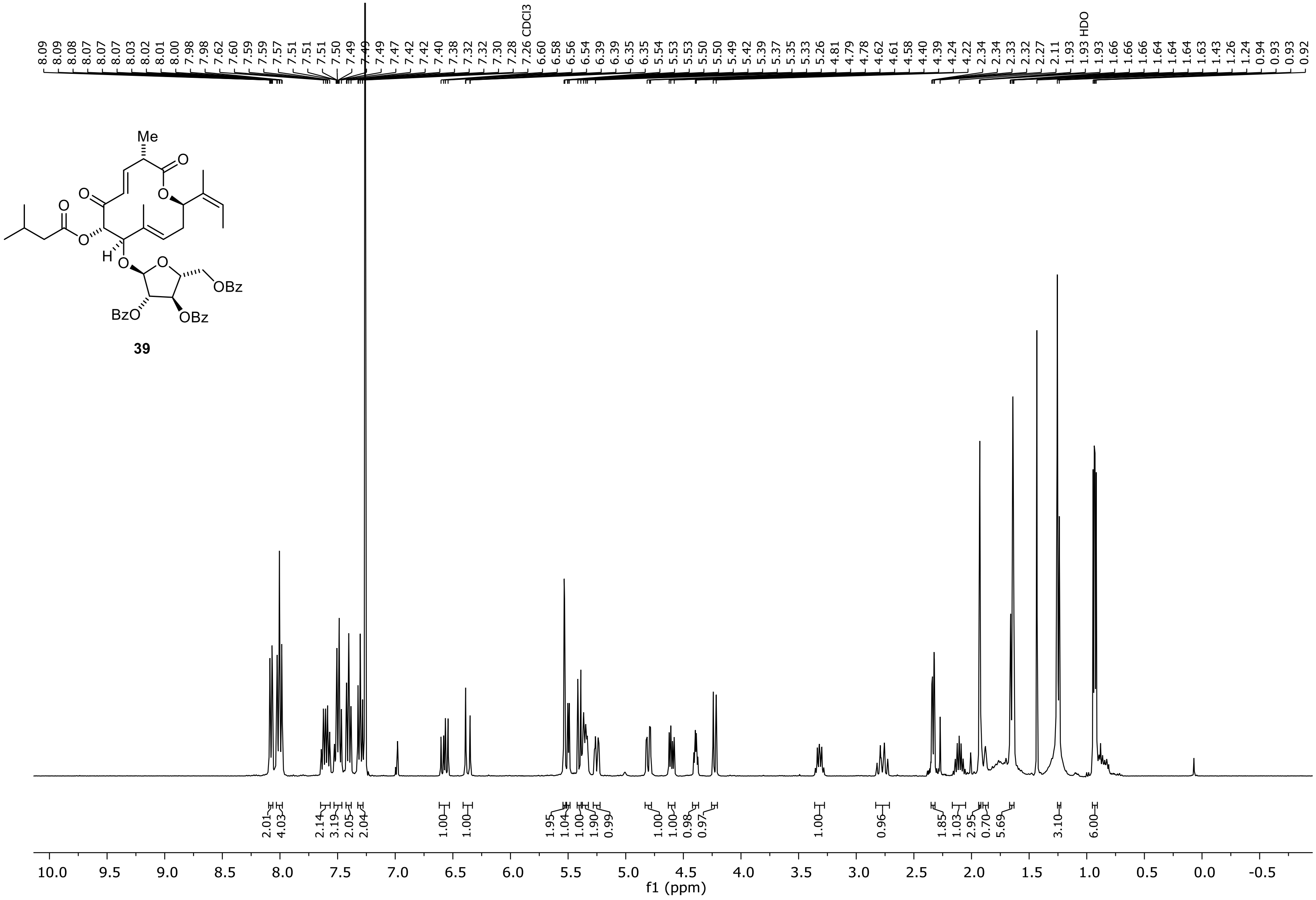

39

13C-NMR (101 MHz, $CDCl_3$):

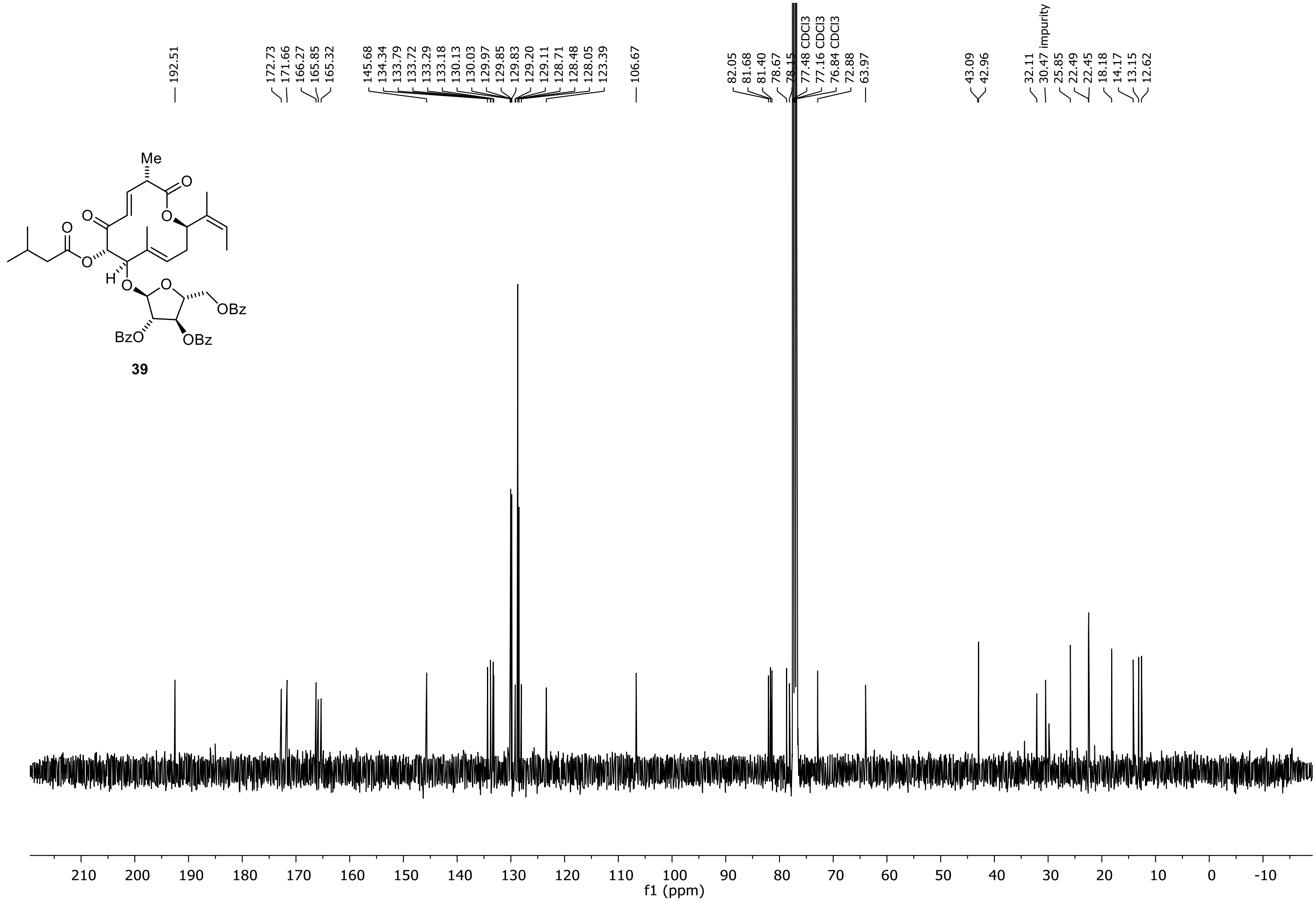

1.3 Crystallographic Data

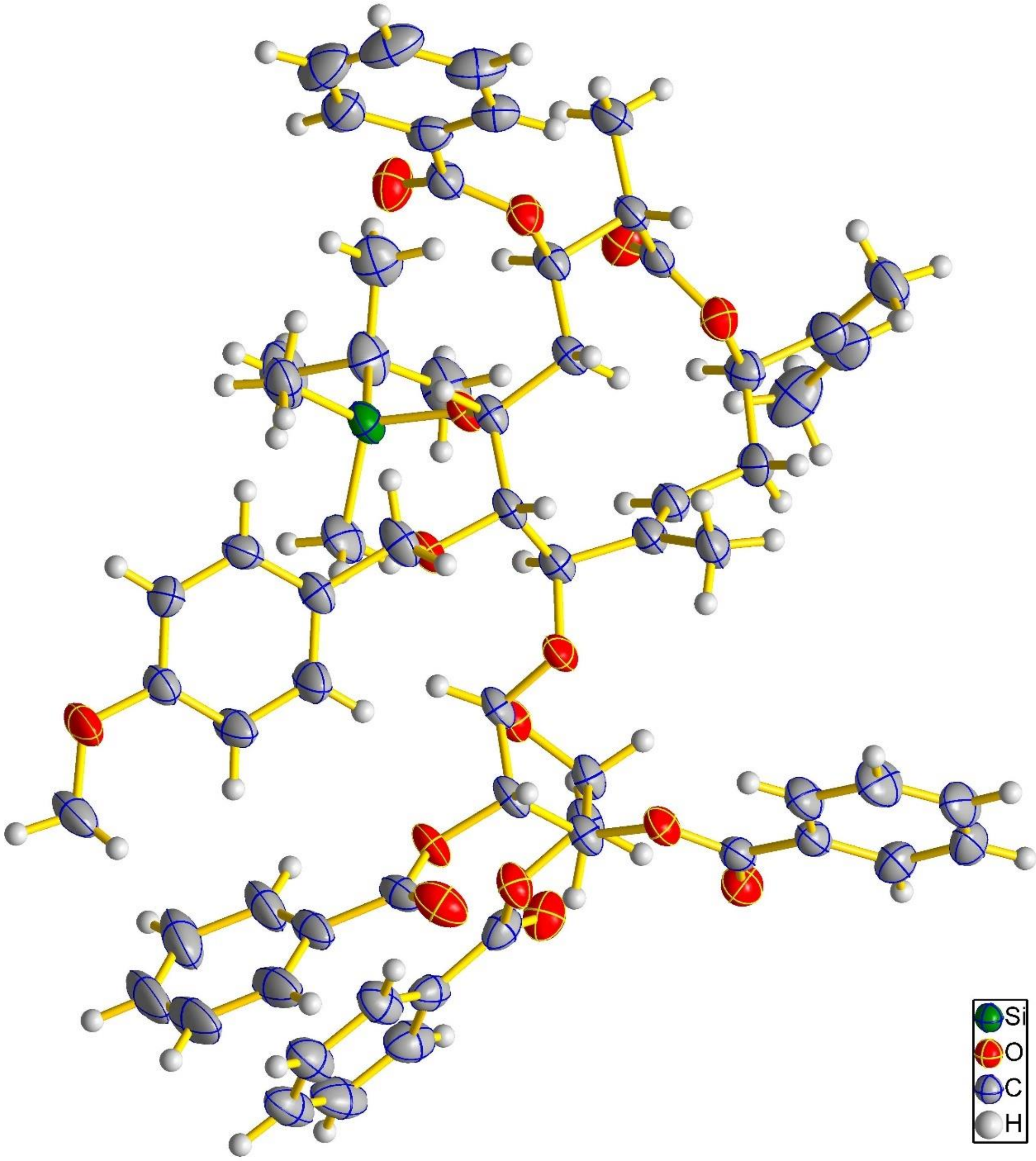

Figure 1.1: ORTEP of the molecular structure of **34.** Ellipsoids are shown at the 50% probability level.

Table 1.1: Crystallographic data for **34.**

net formula	$C_{67}H_{77}O_{15}Si$
M_r/g mol^{-1}	1150.37
crystal size/mm	0.350 x 0.540 x 0.560
T/K	100(2)
radiation	sealed tube, Mo
diffractometer	Bruker APEX-II CCD
crystal system	monoclinic
space group	C 1 2 1
a/Å	32.754(2)
b/Å	18.9382(12)
c/Å	11.2951(7)
α/°	90
β/°	100.6311(10)
γ/°	90
V/Å^3	6886.1(8)
Z	4
calcd. density/g cm^{-3}	1.110
μ/mm^{-1}	0.094
absorption correction	multi-scan
refls. measured	47039
R_{int}	0.0219
θ range	1.25 to 26.39
observed refls.	14131
hydrogen refinement	Full-matrix least-squares on F^2
parameters	758
restraints	10
0.242	0.242
min electron density/e Å^{-3}	-0.246

1.4 References

(23) Kwon, Y.; Schulthoff, S.; Dao, Q. M.; Wirtz, C.; Fürstner, A. Total Synthesis of Disciformycin A and B: Unusually Exigent Targets of Biological Significance. *Chem. – Eur. J.* **2018**, *24* (1), 109–114.

(29) Schreiber, S. L.; Schreiber, T. S.; Smith, D. B. Reactions That Proceed with a Combination of Enantiotopic Group and Diastereotopic Face Selectivity Can Deliver Products with Very High Enantiomeric Excess: Experimental Support of a Mathematical Model. *J. Am. Chem. Soc.* **1987**, *109* (5), 1525–1529.

(30) Jermaks, J.; Tallmadge, E. H.; Keresztes, I.; Collum, D. B. Lithium Amino Alkoxide–Evans Enolate Mixed Aggregates: Aldol Addition with Matched and Mismatched Stereocontrol. *J. Am. Chem. Soc.* **2018**, *140* (8), 3077–3090.

(33) Wang, H.; Ning, J. A One-Pot Strategy for Synthesis of 5-O-(α-D-Arabinofuranosyl)-6-O-(β-D-Galactofuranosyl)-D-Galactofuranose Present in Motif E of the Mycobacterium Wall. *J. Org. Chem.* **2003**, *68* (6), 2521–2524.

(48) Ness, R. K.; Fletcher, H. G. The Anomeric 2,3,5-Tri-O-Benzoyl-D-Arabinosyl Bromides and Other D-Arabinofuranose Derivatives. *J. Am. Chem. Soc.* **1958**, *80* (8), 2007–2010.

(49) Nakatsuka, M.; Ragan, J. A.; Sammakia, T.; Smith, D. B.; Uehling, D. E.; Schreiber, S. L. Total Synthesis of FK506 and an FKBP Probe Reagent, [C(8),C(9)-13C2]-FK506. *J. Am. Chem. Soc.* **1990**, *112* (14), 5583–5601.
